# Chiral macrocyclic terpyridine complexes†
†Electronic supplementary information (ESI) available: Experimental procedures, high-resolution ESI-MS, NMR, UV-Vis spectroscopy and emission decay, cyclic voltammetry measurements, computational methodology, data of the kinetic experiment and crystal refinement details. CCDC 1533847 and 1533848. For ESI and crystallographic data in CIF or other electronic format see DOI: 10.1039/c7sc05285e


**DOI:** 10.1039/c7sc05285e

**Published:** 2018-03-23

**Authors:** Thomas Brandl, Viktor Hoffmann, Andrea Pannwitz, Daniel Häussinger, Markus Neuburger, Olaf Fuhr, Stefan Bernhard, Oliver S. Wenger, Marcel Mayor

**Affiliations:** a Department of Chemistry , University of Basel , St. Johanns-Ring 19 , 4056 Basel , Switzerland . Email: marcel.mayor@unibas.ch; b Institute for Nanotechnology (INT) , Karlsruhe Institute of Technology (KIT) , P. O. Box 3640 , 76021 Karlsruhe , Germany; c Department of Chemistry , Carnegie Mellon University , Pittsburgh , Pennsylvania 15213 , USA; d Lehn Institute of Functional Materials (LFM) , Sun Yat Sen University (SYSU) , XinGangXi Rd. 135 , 510275 Guangzhou , P. R. China

## Abstract

The syntheses of novel chiral M(ii) bis(terpyridine) cage complexes **Fe(L1)_2_-c** and **Ru(L1)_2_-c** are described. The extraordinary design of the precursors **Fe(L1)_2_** and **Ru(L1)_2_** allows perfect preorganization for the final closing step. Due to the rigidity of the spacers between the two terpyridine moieties, the two isolated enantiomers barely racemize at room temperature in solution. The stable and axially chiral bis(terpyridine) Fe(ii) and Ru(ii) complexes were fully characterized by NMR-spectroscopy, UV-Vis spectroscopy, electrochemical measurements, high resolution mass spectrometry, circular dichroism measurements, and X-ray structural analysis.

## Abstract 2

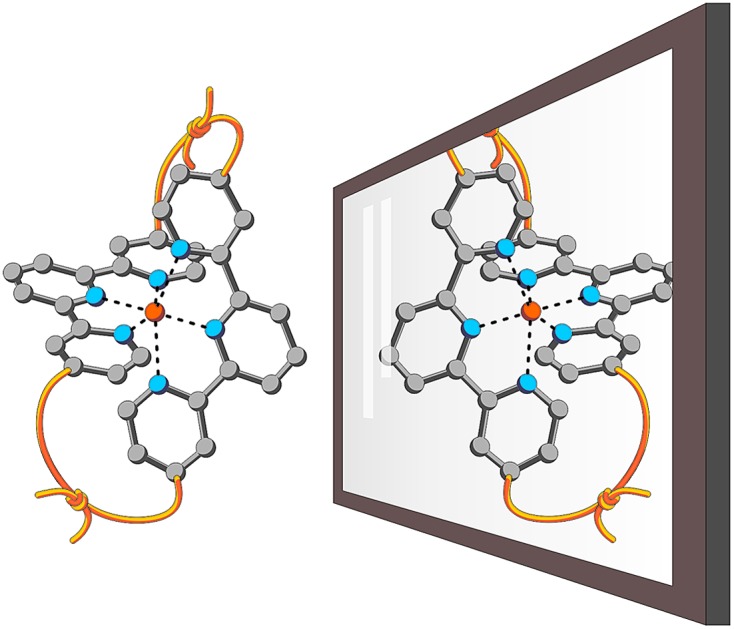
Interlinking of two terpyridine ligands results in mononuclear chiral metal complexes (Fe and Ru) which were separated into their enantiomers.

## Introduction

**Fig. 1:**
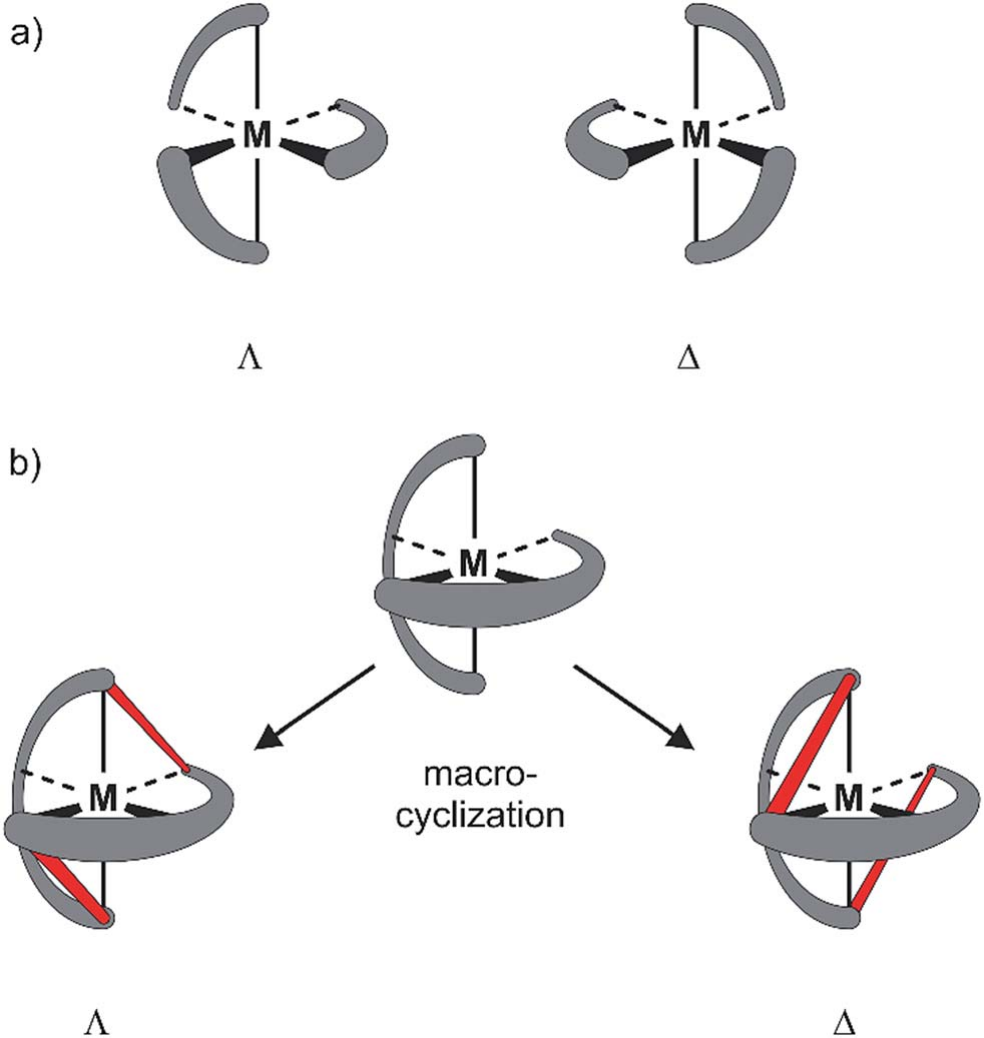
Sketch of axial chirality in metal complexes: (a) axial chirality with bidental ligands (*e.g.* 2,2′-bipyridine). (b) Concept of introducing chirality by macrocyclization. Top: achiral complex of a tridentate ligand (*e.g.* 2,2′:6′,2′′-terpyridine). Bottom: axial chiral metal complexes obtained by interlinking the ends of the two tridentate ligands (interlinking bridges in red).

Since the pioneering work of Louis Pasteur,^1^,^2^ chirality has been a physicochemical property at the center of interest of chemical structures and transformations. Besides chirality induced by stereogenic centers often found in organic compounds, axial chirality emerges from the helical arrangement of the molecule's subunits.^3^ Prominent school book examples for helical chiral metal complexes are tris(2,2′-bipyridine)-M(ii) derivatives. To satisfy the octahedral coordination sphere of the central M(ii) ion, the three ligands wrap around the central metal in a helical arrangement, resulting in a spatial arrangement resembling a ship's propeller. The two enantiomers are distinguished by the helicity of the arrangement between the left-handed (Λ) and the right-handed (Δ) isomer (Fig. 1a).^4^ The helical chiral motif has been studied in detail and often it was complemented by an additional stereogenic center resulting in diastereomeric structures.^5^–^8^ Going from 2,2′-bipyridine to 2,2′:6′,2′′-terpyridine an additional binding site is introduced and thus, only two ligands are required to satisfy the octahedral coordination sphere of a M(ii) ion. The two ligands are arranged perpendicular to each other and the helical chirality is lost. Attempts to maintain chirality in terpyridine complexes range from the attachment of chiral groups^9^–^11^ to their integration into macrocyclic structures.^12^,^13^ Two terpyridines facing each other in the periphery of macrocycles complemented by additional coordination sites result in chiral metal complexes. The most prominent examples are the macrocycle complemented by two additional ethylenediamine units reported by Bazzicalupi *et al.* which forms a dimetallic helical structure upon coordination of two Cu(ii) ions^12^ and the huge macrocycles complemented by two additional phenanthrolines reported by Niess *et al.* which form chiral figure-of-8 shaped Fe(ii) complexes.^13^ While these figure-of-8 complexes are examples of chiral terpyridine complexes, they were only reported as racemic mixtures. Terpyridine complexes have also been used as linkers closing the periphery of macrocycles. In most cases two terpyridines were interlinked at their 4′ positions in order to form macrocyclic structures upon coordination^14^ and in one example, the two enantiomers were identified in the X-ray structure of the compound.^15^ To the best of our knowledge, separated enantiomers of chiral terpyridine complexes have not been reported so far.

Here we present our new strategy to introduce axial chirality into metal terpyridine complexes, namely by twofold interlinking of the ligands surrounding the metal ion. The concept is sketched in Fig. 1b and can also be described as a template supported macrocyclization reaction. The two terpyridines close a macrocycle which has been preorganized (templated) by the formation of the metal-terpyridine complex. The macrocycles in the complex are arranged in an axially chiral arrangement resulting in axially chiral complexes with the M-isomer of the macrocycle surrounding the Λ-complex and the macrocycle's P-isomer around the Δ-complex. For this purpose, the terpyridines are complemented by two *ortho*-ethynylphenyl moieties in 4 and 4′′ positions allowing for macrocyclization by *Glaser-Hay* type oxidative acetylene coupling chemistry. According to simple MM2 based molecular models, we hypothesized that there should exist rotamers of the metal complex having the ethynyl groups of neighboring ligands in close proximity favoring intramolecular coupling over intermolecular oligomerization.

While oxidative acetylene protocols have been used extensively for macrocyclization^16^ and also by ourselves,^17^,^18^ the so far reported examples were usually not preorganized in metal complexes. Preorganization in metal complexes has been pioneered by Jean-Pierre Sauvage and has been used extensively for the assembly of mechanically interlinked superstructures comprising rotaxanes,^19^ catenanes^19^ and even more complex objects like molecular knots.^20^ Surprisingly, in spite of the mild reaction conditions, oxidative acetylene coupling has only been used occasionally for the assembly of these structures.^21^–^24^

## Results and discussion

**Scheme 1:**
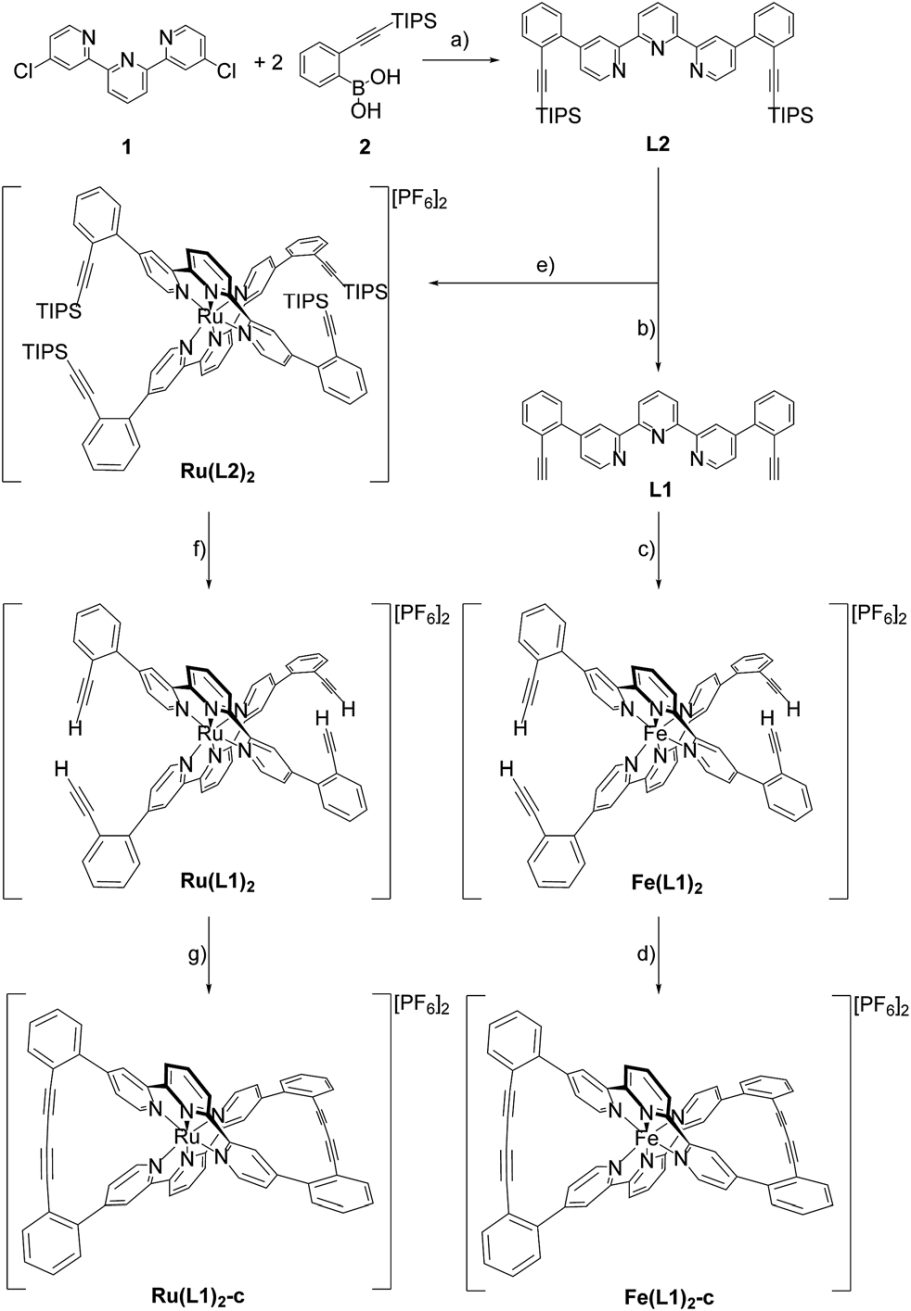
Syntheses of the axial chiral complexes **Fe(L1)_2_-c** and **Ru(L1)_2_-c**. For clarity only the Λ enantiomer is displayed. Reagents and conditions: (a) K_2_CO_3_, Pd(amphos)Cl_2_, toluene/water (5 : 1), 116 °C, 24 h, 87%; (b) TBAF, DCM, RT, 1 h, quant.; (c) (1) FeCl_2_, MeOH/DCM (1 : 5), RT, 3 h; (2) NH_4_PF_6_, water, overnight, 95%; (d) O_2_, CuCl, TMEDA, DCM, RT, 12 h, 89%; (e) (1) RuCl_3_·*x*H_2_O, AgBF_4_, DMF, reflux, 3.5 d; (2) NH_4_PF_6_ 31%; (f) TBAF, DCM, RT, 10 min, quant.; (g) O_2_, CuCl, TMEDA, DCM, RT, 12 h, 96%.

Here we report the syntheses and isolation of both enantiomers of axial chiral iron and ruthenium complexes **Fe(L1)_2_-c** and **Ru(L1)_2_-c** (Scheme 1), and their full characterization complemented by preliminary racemization studies documenting the considerable chiral stability of the macrocyclized ligand system.

The syntheses of the caged complexes **Fe(L1)_2_-c** and **Ru(L1)_2_-c** are displayed in Scheme 1. The ligand precursor **1** was assembled following a published protocol^25^ and also its reactivity in *Suzuki*-type coupling reactions has already been reported.^25^,^26^ With the improved catalyst system reported by Guram and coworkers,^27^ the reaction between terpyridine **1** and boronic acid **2** gave the ligand **L2** exposing two tris-isopropylsilyl (TIPS) protected ethynyl groups. Unfortunately, we were not able to find reaction conditions guaranteeing full conversion and thus, the target ligand **L2** could only be separated from a monosubstituted side-product by automated size exclusion chromatography. For the assembly of the Fe(ii) complex the two ethynyl groups were first liberated by treatment with tetrabutylammoniumfluoride (TBAF) giving the bis-ethynyl functionalized ligand **L1**. In our first attempt the iron complex was synthesized with the TIPS masked ligand **L2**, but this strategy was revised quickly as the **Fe(L2)_2_** complex was unstable when exposed to fluoride ions. The homoleptic **Fe(L1)_2_** complex was obtained by the addition of FeCl_2_ to a solution of **L1** in a mixture of methanol (MeOH) and dichloromethane (DCM). The instantaneous color change to deep purple indicated the complexation and after the removal of the solvents and the addition of water, the complex **Fe(L1)_2_** was isolated in good yield (95%) upon precipitation with an excess of PF_6_^–^ ions.

**Fig. 2:**
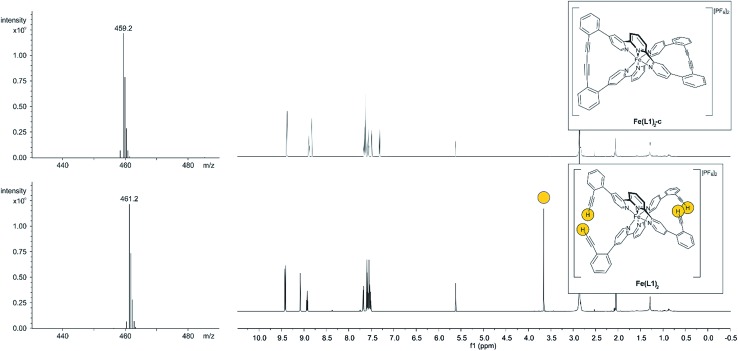
ESI-mass spectrometry signals (left side) and ^1^H-NMR spectra (right side) of the precursor **Fe(L1)_2_** (bottom) and the macrocyclized **Fe(L1)_2_-c** (top). The acetylene H-atoms and their signal are highlighted by dark yellow circles. The reduction of the mass by 2 *m*/*z* units and the disappearance of the acetylene H-atoms in the NMR spectrum indicate the successful macrocyclization.

With the iron complex **Fe(L1)_2_** exposing four ethynyl groups in hand, its behavior under acetylene coupling conditions moved into the focus of interest. Thus the compound was exposed to commonly used *Hay* conditions.^28^ The complex dissolved in DCM was added to an oxygen saturated solution of CuCl and tetramethylethylenediamine (TMEDA) in DCM and the reaction mixture was vigorously stirred to provide oxygen from the oxygen atmosphere. After 12 hours of stirring at room temperature, the reaction was monitored by thin layer chromatography (TLC) and ESI-ToF-mass spectrometry (ESI-MS). While the TLC (reversed phase, in a 9/1 acetonitrile/water mixture) displayed spot-to-spot conversion, exclusively a molecular ion with *m*/*z* = 459.2 was observed by ESI-MS corresponding to the signal of the doubly charged cation of the macrocyclic complex **Fe(L1)_2_-c** and the signal of the cation of the open precursor complex **Fe(L1)_2_** at *m*/*z* = 461.2 was no longer present. The reaction mixture was worked up and the isolated purple solid was repeatedly washed with hexane. The completeness of the macrocyclization by oxidative acetylene coupling was displayed by the comparison of the crude ^1^H-NMR spectra of **Fe(L1)_2_-c** and its precursor **Fe(L1)_2_**. As displayed in Fig. 2, the acetylene H-signal at 3.63 ppm (indicated by dark yellow circles at the bottom of Fig. 2) disappeared completely during the macrocyclization reaction, pointing at the complete transformation of the acetylene groups of **Fe(L1)_2_** into diacetylenes in **Fe(L1)_2_-c**.

A similar synthetic strategy for the ruthenium complex **Ru(L1)_2_** failed, probably due to competing side reactions of the ethynyl groups at the elevated temperature required for the complexation. Using the more stable TIPS-protected ligand **L2** the corresponding ruthenium complex **Ru(L2)_2_** was obtained according to a reported procedure^29^ by treating RuCl_3_ with AgBF_4_ in refluxing dimethylformamide (DMF) before **L2** was added and the reaction mixture was refluxed for 3.5 days. Precipitation from aqueous NH_4_PF_6_ and excessive purification by column chromatography (CC) provided **Ru(L2)_2_** in moderate yield (31%) as a red solid. The increased stability of the ruthenium complex allowed for quantitative deprotection of the ethynyl groups with TBAF in DCM at room temperature providing the complex **Ru(L1)_2_**. Applying similar macrocyclization conditions to those for **Fe(L1)_2_** provided macrocyclized **Ru(L1)_2_-c** in an even better isolated yield of 96% as a red solid.

The importance of the preorganization of both ligands for the macrocyclization became obvious when high-dilution strategies to assemble the macrocycle failed (see the ESI†).

**Fig. 3:**
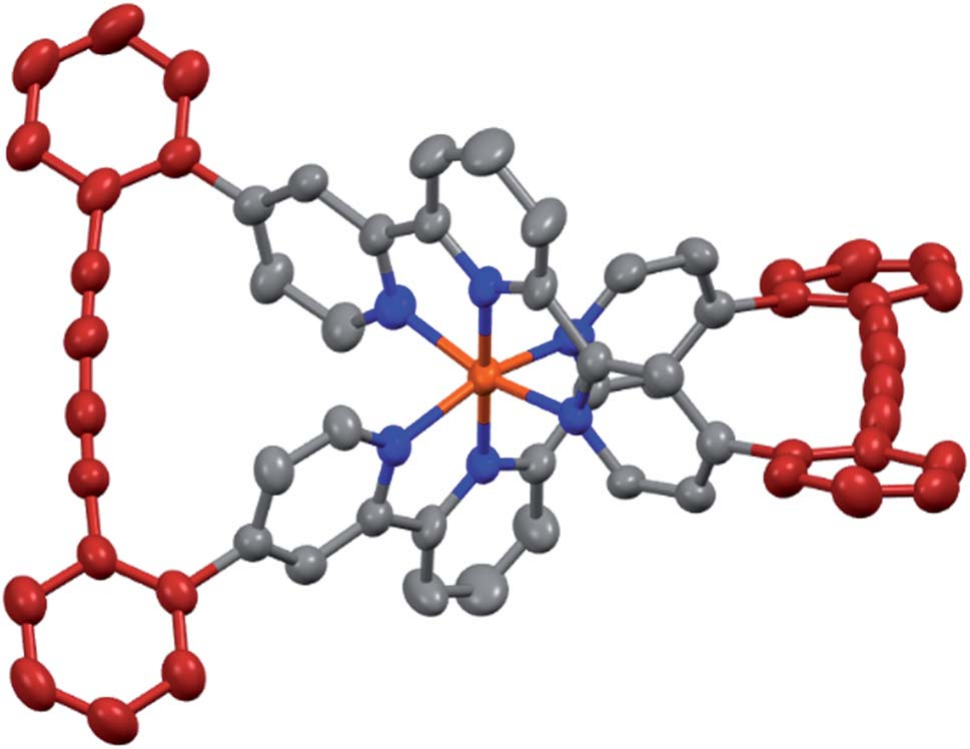
Solid state structure of the Λ enantiomer of **Fe(L1)_2_-c** with rotation ellipsoids at 50% probability. Hydrogen atoms and the PF_6_^–^ counter ions are omitted for clarity.

The target complexes and their precursors were fully characterized by ^1^H NMR and ^13^C NMR spectroscopy, high-resolution mass spectrometry, and UV-Vis spectroscopy. In addition, the identities of the macrocyclized complexes **Fe(L1)_2_-c** and **Ru(L1)_2_-c** were corroborated by their solid state structures. Single crystals of **Fe(L1)_2_-c** and **Ru(L1)_2_-c** suitable for X-ray analysis were obtained by vapor diffusion using diethyl ether/acetone and diethyl ether/acetonitrile, respectively. As a representative example the structure of **Fe(L1)_2_-c** is displayed in Fig. 3 and the structure of **Ru(L1)_2_-c** is displayed in the ESI.†

Both complexes crystallize as a racemate and in spite of their structural similarity, a surprisingly large difference in their solid state packing was observed (Fig. S12 and S13†). While **Fe(L1)_2_-c** displayed a compact packing with π-stacking interactions between alternating enantiomers, there is no similar intermolecular interaction observed for **Ru(L1)_2_-c**. The solid state structures display the compact arrangement of the axially chiral macrocyclic ligand surrounding the central metal ion. In the macrocyclized complexes the octahedral coordination sphere is distorted as the planes defined by both terpyridines have an angle of 76.72° for **Fe(L1)_2_-c** and 75.11° for **Ru(L1)_2_-c** and are no longer perpendicular to each other. In spite of this distortion, the **Fe(L1)_2_-c** remains in its low spin state indicated by its purple color. The diacetylene bridges in both macrocyclized complexes are only slightly bent with angles between both triple bonds of 173.5° and 175.7° for **Fe(L1)_2_-c** and **Ru(L1)_2_-c**, respectively. Their minor deviation from linearity points at both the perfect match of the ethynyl-preorganization in the precursors and the structural integrity and stability of the macrocyclized complexes.

The redox properties of the open and macrocyclized complexes were analyzed by cyclic voltammetry in DCM with 0.1 M tetrabutylammonium hexafluorophosphate (TBAPF_6_) as the supporting electrolyte. The voltammograms were recorded against ferrocene as the internal standard and are displayed in the ESI (Fig. S3†). For all 4 complexes one oxidation wave and two reduction waves were observed and the extracted redox potentials are listed in Table 1.

**Table 1:** Measured oxidation and reduction potentials of the open precursor and the macrocyclized Fe(ii) and Ru(ii) complexes. The potentials are listed in volts against Fc^+^/Fc

Complex/*E*^0^ [V]	*E*_ox_	*E*_red1_	*E*_red2_
**Fe(L1)_2_**	0.72	–1.58	–1.79
**Fe(L1)_2_-c**	0.82	–1.53	–1.76
**Ru(L1)_2_**	0.89	–1.59	–1.88
**Ru(L1)_2_-c**	0.99	–1.54	–1.87

Comparing both open precursors with their macrocyclized complexes, a 100 mV increase of the potential required to oxidize the macrocyclized species was observed in both cases, while only minor shifts (almost within the accuracy of the analysis method) were recorded for the reduction potentials. In analogy to reported Ru(ii) and Fe(ii) terpyridine complexes,^30^,^31^ a metal centered oxidation process was assumed. The increase of the oxidation potentials for the Fe complex upon macrocyclization was rationalized by the increased rigidity of the terpyridine subunits in the macrocycle which reduces its ability to adapt its coordination sphere to the decreased dimensions of the oxidized Fe(iii) ion.

In the case of the Ru complexes the bond lengths do not vary significantly between Ru(ii) and Ru(iii) polypyridyl complexes^32^ and thus, the shift in the oxidation potential upon macrocyclization probably reflects the increased electronic shielding of the central Ru ion.

**Fig. 4:**
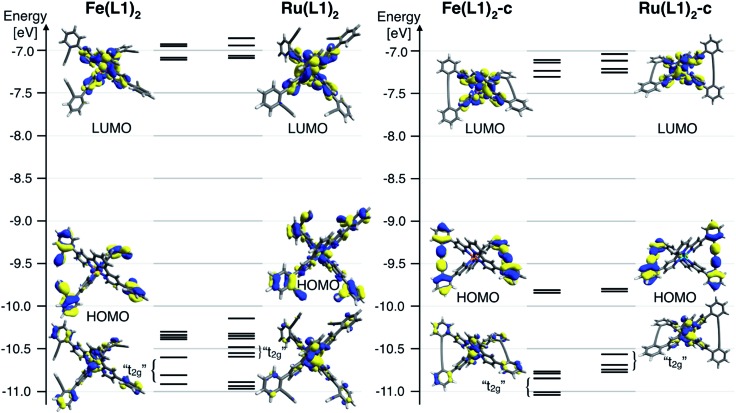
Frontier molecular diagram of the open (left) and the closed (right) form of the Fe(ii) and Ru(ii) complexes generated from DFT calculations (B3LYP/LANL2DZ). The rendered 98% surfaces of selected orbitals are also depicted.

To further rationalize the results obtained by the cyclic voltammetry, DFT calculations were performed. Surprisingly, the highest occupied molecular orbital (HOMO) is according to these calculations not centered on the metal, as it would be expected for Ru(ii) and Fe(ii) terpyridine complexes.^30^,^31^ The HOMO of the open system is predominately localized on the ligand and possesses a small metal-character. The coupling of the ligands localizes the HOMO entirely on the phenyl acetylene moieties with even less metal character. The metal-centered orbitals reminiscent of the t_2g_ orbitals in an *O*_h_ symmetric environment can be found below the HOMO for all four complexes (Fig. 4). For these metal-centered orbitals the trend to higher oxidation potentials upon macrocyclization is also found in the DFT calculation. The metal center and the phenyl alkyne moieties are according to DFT electronically isolated. It is unsurprising that the metal-based oxidation processes proceed at potentials identical to those observed for the [M(tpy)_2_]^2+^ parent complex while the hard to oxidize phenyl alkyne units remain unaffected. For comparison, the measured oxidation potential of 1,4-diphenyl-1-3-butadiyne is 0.6 V higher than that of [Ru(tpy)_2_]^2+^ and the difference is even more substantial when considering its iron analogue.^33^ Electrochemical oxidation processes can involve considerable structural and solvation changes and it is therefore problematic to use orbital energies for predicting trends in structurally diverse molecules and molecular subunits. The DFT modelling indicates that the lowest unoccupied molecular orbital (LUMO) for all closed and open complexes is localized on the terpyridine moiety, as expected and in line with the CV data. The character of the LUMOs matches that of the π* orbitals in the bis-terpyridine parent complexes. The enlarged π system does not substantially shift the reduction potentials in both the closed and open derivatives. It seems that the almost orthogonal arrangement of the phenyl acetylene moieties hinders the conjugation throughout the whole ligand system, limiting the extent of the LUMO to the terpyridine section of the ligand.

The optical absorption spectra of the complexes are displayed in the ESI (Fig. S2†). For both metal ions comparing the UV-Vis spectra of the open and the macrocyclized complexes, the common metal-to-ligand charge transfer (MLCT) and the ligand centered (LC) bands do not shift, but increase in intensity. The increase is more pronounced in the LC band and probably arises from the somewhat increased π system upon diacetylene formation in the macrocyclization reaction.

Of particular interest was the influence of the macrocyclization on the photophysical properties of the complexes. In analogy to the Ru(tpy)_2_^2+^ parent complex, **Ru(L1)** and **Ru(L1)_2_-c** are non-emissive at room temperature, but photoluminescence is readily detectable in frozen matrices. In frozen butyronitrile at 77 K, the luminescence lifetimes are 10.5 μs for **Ru(L1)** and 8.6 μs for **Ru(L1)_2_-c**. For Ru(tpy)_2_^2+^ a luminescence lifetime of 8.0 μs was determined under similar conditions.^34^ Evidently, the macrocyclization has very limited influence on the photoluminescence properties, and it seems that ^3^MLCT deactivation *via* energetically close-by metal centered (MC) excited states remains efficient in **Ru(L1)** and **Ru(L1)_2_-c**.

**Fig. 5:**
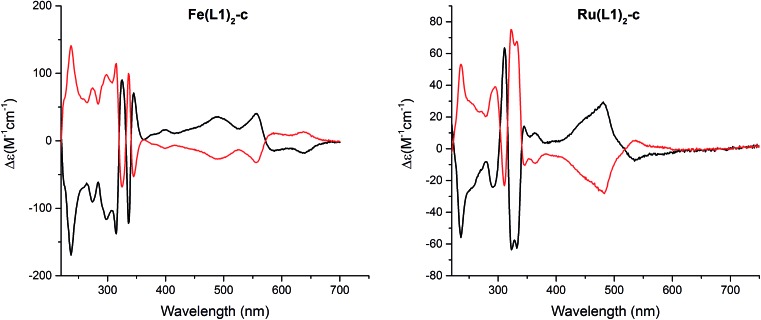
Circular dichroism spectra of both enantiomers of [**Fe(L1)_2_-c**]^2+^[PF_6_^–^]_2_ (left) and [**Ru(L1)_2_-c**]^2+^[PF_6_^–^]_2_ (right) recorded in acetonitrile at room temperature.

The macrocyclization of the complexes provides racemic mixtures of both helical chiral enantiomers. Interestingly, the stiffening of the terpyridine ligand arrangement obtained by macrocyclization results in a considerable increase in the stability of the enantiomers. Racemates of both the Fe(ii) and the Ru(ii) complexes could be separated into their enantiomers by high-performance liquid chromatography (HPLC) using a chiral stationary phase (Chiralpak IB, eluent EtOH/MeOH/TEA/TFA, 50 : 50 : 0.5 : 0.3, 1 mL min^–1^, and *T* = 40 °C). The circular dichroism (CD) spectra of both enantiomers are displayed in Fig. 5 and for both complexes, **Fe(L1)_2_-c** and **Ru(L1)_2_-c**, Cotton effects were observed in the entire detectable spectral range with opposite signs for both enantiomers. Only small samples of pure enantiomers were obtained due to the analytical character of the column. In spite of intense efforts, attempts to simulate the CD spectra have not been conclusive so far and thus, the assignment of the CD spectra to a particular enantiomer is currently not possible.

However, the availability of enantiomerically pure samples allowed for preliminary racemization studies investigating the stability of the macrocyclized complexes. The initial attempts to trigger the racemization optically or electrochemically failed, pointing at the stability of the macrocyclized architectures. Also heating of the **Ru(L1)_2_-c** complex in various solvents at elevated temperature (*e.g.* butyronitrile at 200 °C oil bath temperature in a pressure tube or tetrachloroethane at 140 °C oil bath temperature) for prolonged periods did not show any racemization, pointing at the stability of the helical chiral species. Even more surprising was the stability of **Fe(L1)_2_-c** as to the best of our knowledge, Fe(ii) terpyridine complexes that do not racemize in solution have not been reported so far. And indeed, after screening of various solvents (see the ESI†) only 1,1,2,2-tetrachloroethane had a high enough boiling point allowing us to observe the temperature induced racemization of **Fe(L1)_2_-c**. To get a first idea of the thermodynamics of the racemization, the process was monitored by HPLC at temperatures between 60 °C and 90 °C (see the ESI†). With this rudimentary analysis an inversion barrier of 117 kJ mol^–1^ at 25 °C was estimated, corresponding to a half-lifetime at room temperature in the order of 163 days.

## Conclusion

In summary, a new type of compact helical chiral terpyridine metal complex is presented based on the metal templated macrocyclization of suitably functionalized terpyridine ligands. The macrocyclization based on oxidative acetylene coupling within the complex proceeds in excellent yields and the isolated Fe(ii) and Ru(ii) complexes are fully characterized by their solid-state structures. The obtained racemates of helical chiral complexes can be separated into the corresponding enantiomers by chromatography with chiral stationary phases. The reinforcement of the terpyridine ligand sphere upon macro-cyclization considerably increases the stability of the enantiomers and its influence on physical properties like redox behavior and optical features is discussed.

We are currently investigating alternative macrocyclization reactions and are interested in approaches enabling an even tighter fixation of the surrounding ligand sphere.

## Conflicts of interest

There are no conflicts to declare.

## Supplementary Material

Supplementary information

Crystal structure data
