# Supplementary material for: Chiral macrocyclic terpyridine complexes
Source: Chem Sci. 2018 Mar 23;9(15):3837–43. doi: 10.1039/c7sc05285e (PMC5941204; doi:10.1039/c7sc05285e)
Supplement: Supplementary file 1 [file SC-009-C7SC05285E-s001.pdf]

Electronic Supplementary Material (ESI) for Chemical Science.
This journal is © The Royal Society of Chemistry 2018

Supporting Information

Chiral Macrocyclic Terpyridine Complexes

Thomas Brandl[a], Viktor Hoffmann[a], Andrea Pannwitz[a], Daniel Häussinger[a], Markus Neuburger[a], Olaf Fuhr[b], Stefan Bernhard[c], Oliver S. Wenger[a], Marcel Mayor*[a,b,d]

a Department of Chemistry
University of Basel
St. Johanns-Ring 19
4056 Basel, Switzerland
E-mail: marcel.mayor@unibas.ch

b Institute for Nanotechnology (INT)
Karlsruhe Institute of Technology (KIT)
P. O. Box 3640
76021 Karlsruhe, Germany

c Department of Chemistry
Carnegie Mellon University
Pittsburgh
Pennsylvania 15213, USA

d Lehn Institute of Functional Materials (LFM)
Sun Yat Sen University (SYSU), XinGangXi Rd. 135
510275 Guangzhou (P. R. China)

Table of Contents:

Table of Contents S2

Synthetic procedures S3

^{1}H-, ^{13}C-NMR ($CDCl_3$, 400/101 MHz, 22 °C) and HR-ESI spectra of **L2** S13

^{1}H-, ^{13}C-NMR ($CDCl_3$, 600/151 MHz, 25 °C) and HR-ESI spectra of **L1** S15

^{1}H-, ^{13}C-NMR ($CDCl_3$, 600/151 MHz, 25 °C) and HR-ESI spectra of **Fe(L1)$_2$** S17

^{1}H-, ^{13}C-NMR, COSY, NOESY, HMBC, HMQC ($CDCl_3$, 600/101/150 MHz, 25 °C) and HR-ESI spectra of **Fe(L1)$_2$-c** with full assignment S19

^{1}H-, ^{13}C-NMR ($CDCl_3$, 400/101 MHz, 22 °C) and HR-ESI spectra of **Ru(L2)$_2$** S24

1H-, 13C-NMR (CDCl3, 600/151 MHz, 25 °C) and HR-ESI spectra of **Ru(L1)$_2$** S26

^{1}H-, ^{13}C-NMR, COSY, NOESY, HMBC, HMQC ($CDCl_3$, 600/151 MHz, 25 °C) and HR-ESI spectra of **Ru(L1)$_2$-c** with full assignment S28

Decay of emission signal of the complexes **Ru(L1)$_2$-c** and **Ru(L1)$_2$** at 77 K S33

UV-Vis spectroscopy S34

Cyclic Voltammetry measurements S35

Computational Methodology S36

Racemization experiment and determination of the inversion barrier of **Fe(L1)$_2$-c**: S37

Crystal data for **Ru(L1)$_2$-c** and **Fe(L1)$_2$-c**: S46

HPLC Chromatograms S53

High dilution macrocyclization experiment of the free ligand **L1** S54

Synthetic procedures:

General Procedures: All chemicals were directly used for the synthesis without further purification, unless stated differently. Solvents for electrochemical and photophysical measurements were HPLC grade. Reaction vessels used for acetylene deprotection were washed with concentrated sulfuric acid to prevent copper promoted oxidative acetylene homocoupling. Dry solvents were used as crown cap and purchased from *Acros Organics* and *Sigma-Aldrich*. NMR solvents were obtained from *Cambridge Isotope Laboratories, Inc.* (Andover, MA, USA). ^{1}H-NMR and ^{13}C-NMR were recorded on a *Bruker DPX-NMR* (400 MHz) instrument or on a Bruker Ascend 600 MHz Avance III HD. Chemical shifts (δ) are reported in parts per million (ppm) relative to the residual solvent peak. Coupling constants (*J*) are given in hertz (Hz). Gas Chromatography (GC-MS) was performed on a *Shimadzu* GCMS-QP2010 SE gas chromatograph system, with a ZB-5HT inferno column (30 m x 0.25 mm x 0.25 mm), at 1 mL/min He-flow rate (split = 20:1) with a *Shimadzu* mass detector (EI 70 eV) was used. For high resolution mass spectra (HRMS) a HR-ESI-ToF-MS measurement on a *maXis^{TM} 4G* instrument from *Bruker* was performed. MALDI-ToF mass spectra were performed on a *Bruker microflex*™ mass spectrometer, calibrated with CsI_3, and α-cyano-4-hydroxycinnamic acid (unless stated differently) was used as matrix Column chromatography was performed with SiliaFlash® P60 from *SILICYCLE* with a particle size of 40-63 μm (230-400 mesh) and for TLC *Silica gel 60 F_{254}* glass plates with a thickness of 0.25 mm from *Merck* were used. The detection was observed with a UV-lamp at 254 or 366 nm. Gel Permeation Chromatography (GPC) was performed on a *Shimadzu Prominence* System with *PSS SDV* preparative columns from *PSS* (2 columns in series: 600 mm x 20.0 mm, 5 μm particles, linear porosity “S“, operating ranges: 100 – 100 000 g.mol^{-1}) using chloroform as eluent. For HPLC a Shimadzu LC-20AD and a LC-20AT HPLC, respectively, was used equipped with a diodearray UV/Vis detector (SPD-M10A VP from Shimadzu, λ = 200-600 nm) and a column oven Shimadzu CTO-20AC. The used column for reverse phase was a Reprosil 100 C18, 5 μm, 250 x 16 mm; Dr. Maisch GmbH and for chiral separation a Chiralpak IB, 5 μm, 4.6 x 250 mm; Daicel Chemical Industries Ltd. CD measurements were performed with a Chirascan CD Spectrometer in acetonitrile at room temperature in 1 cm quartz glass cuvettes.

((2-Bromophenyl)ethynyl)triisopropylsilane (4):

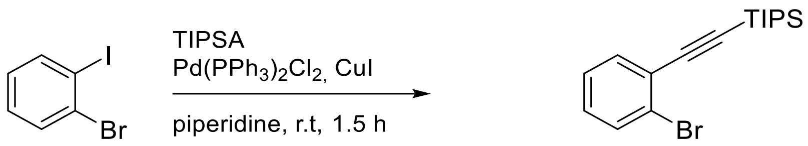

4

An oven-dried 25 mL Schlenk tube was purged with argon and charged with 1-bromo-2-iodobenzene (3.30 g, 11.7 mmol, 1.0 eq.), bis(triphenylphosphine) palladium(II) chloride (207 mg, 293 μmol, 2 mol-%), copper(I) iodide (89.6 mg, 468 μmol, 4 mol-%) and piperidine (30 mL). The mixture was degassed with argon for 15 minutes and then (triisopropylsilyl) acetylene (2.79 mL, 12.1 mmol, 1.03 eq.) was added. The reaction mixture was stirred at room temperature overnight. After TLC confirmed full consumption of the starting material, the reaction was stopped by removing the solvent under reduced pressure. The remains were eluted with CH_2Cl_2 and again concentrated on Celite to purify the crude product via flash column chromatography (cyclohexane/ethyl acetate = 20:1) to obtain a pale orange liquid (4.00 g, 11.7 mmol, 100%).

Analytical data for 4:

^{1}H NMR (400 MHz, $CDCl_3$, 22 °C) δ = 7.57 (dd, $^{3}J_{H,H}$ = 7.9, $^{4}J_{H,H}$ = 1.3 Hz, 1H), 7.52 – 7.49 (m, 1H), 7.23 (dd, $^{3}J_{H,H}$ = 7.6, $^{4}J_{H,H}$ = 1.3 Hz, 1H), 7.15 (ddd, $^{3}J_{H,H}$ = 8.0, $^{3}J_{H,H}$ = 7.4, $^{4}J_{H,H}$ = 1.7 Hz, 1H), 1.15 (m, 21H) ppm.

^{13}C NMR (101 MHz, $CDCl_3$, 22 °C) δ = 134.00, 132.50, 129.50, 126.97, 125.90, 125.81, 104.91, 96.34, 18.82, 11.46 ppm.

GC-MS (EI +, 70 eV): m/z (%) = 336 (4) [M+], 293 (100) [M+-C_3H_7], 267 (32), 251 (23), 237 (32), 223 (41), 129 (33).

The analytical data are in agreement with the ones reported in reference [1].

[1] T. Ide, S. Sakamoto, D. Takeuchi, K. Osakada, S. Machida, *J. Org. Chem.* **2012**, *77*, 4837–4841.

(2-((Triisopropylsilyl)ethynyl)phenyl)boronic acid (2):

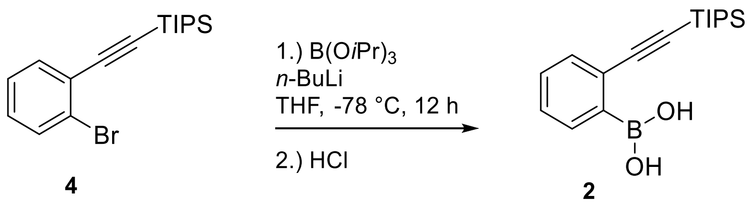

An oven-dried two-necked round-bottomed flask was charged with 1-bromo-2-(triisopropyl-silyl-1-ynyl)benzene (**4**, 2.00 g, 5.93 mmol, 1.0 eq.) and dry THF (30 mL). The reaction mixture was cooled to -78 °C, before *n*-BuLi (1.6 M in hexane, 7.41 mL, 11.9 mmol, 2.0 eq.) was added drop-wise. After 30 minutes, B(O*i*Pr)$_3$ (3.43 mL, 14.8 mmol, 2.0 eq.) was added and the reaction mixture was stirred at -78 °C gradually warming up to room temperature overnight. The reaction was quenched with aqueous HCl solution (1 M, 20 mL) and then extracted with Et_2O, dried over $MgSO_4$, filtered and concentrated under reduced pressure. The residue was purified by flash column chromatography (cyclohexane/ethyl acetate = 10:1) to give the title compound as a white solid (1.66 g, 5.48 mmol, 92%).

Analytical data for 2:

^{1}H NMR (400 MHz, $CDCl_3$, 22 °C) δ = 8.01 – 7.96 (m, 1H), 7.56 – 7.52 (m, 1H), 7.40 (m, 2H), 5.81 (s, 2H), 1.16 (m, 21H) ppm.

ESI-MS (MeCN, positive mode): *m/z* 609.4 [2M - H_2O + Na^+].

The analytical data are in agreement with the ones reported in reference [1].

[1] T. Ide, S. Sakamoto, D. Takeuchi, K. Osakada, S. Machida, *J. Org. Chem.* **2012**, *77*, 4837–4841.

4,4''-Bis(2-((triisopropylsilyl)ethynyl)phenyl)-2,2':6',2''-terpyridine (L2):

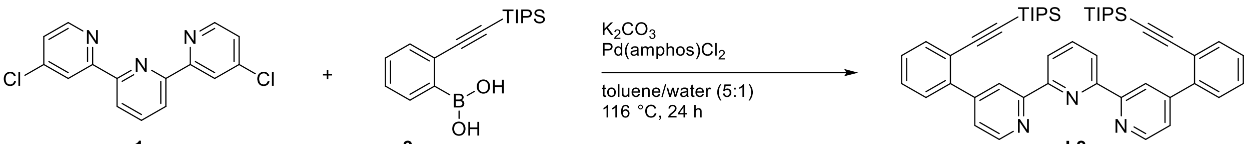

1 **2** **L2**

A 50 mL round bottom flask was set under argon atmosphere and was charged with 4,4''-dichloro-2,2':6',2''-terpyridine (**1**, 187 mg, 619 μmol, 1.0 eq.), (2-((triisopropylsilyl)ethynyl)phenyl)boronic acid (**2**, 748 mg, 2.47 mmol, 4.0 eq.), K_2CO_3 (173 mg, 1.24 mmol, 2.0 eq.), bis(di-tert-butyl(4-dimethylaminophenyl)phosphine)dichlorpalladium(II) (21.9 mg, 31.0 μmol, 5 mol-%) and the solvents toluene/water (30/6). The reaction mixture was degassed before it was heated to 116 °C for 18 hours. After TLC confirmed full conversion of the starting material, the reaction was stopped, the aqueous phase was extracted with CH_2Cl_2. The combined organic layers were washed with brine, dried over $MgSO_4$, filtered and concentrated under reduced pressure. The crude product was purified by column chromatography (cyclohexane:ethyl acetate = 10:1 + 2% NH_4OH) and GPC to yield the desired product as colorless oil (401 mg, 619 μmol, 87%).

Analytical data for L2:

^{1}H NMR (600 MHz, $CDCl_3$, 25 °C): δ = 8.70 (dd, $^3J_{H,H}$ = 5.0, $^4J_{H,H}$ = 0.8 Hz, 2H), 8.67 (dd, $^4J_{H,H}$ = 1.8, $^4J_{H,H}$ = 0.8 Hz, 2H), 8.47 (d, $^3J_{H,H}$ = 7.8 Hz, 2H), 7.98 (t, $^3J_{H,H}$ = 7.8 Hz, 1H), 7.63 (dd, $^3J_{H,H}$ = 7.7, $^4J_{H,H}$ = 1.4 Hz, 2H), 7.60 (dd, $^3J_{H,H}$ = 5.0, $^4J_{H,H}$ = 1.8 Hz, 2H), 7.46 (dd, $^3J_{H,H}$ = 7.6, $^4J_{H,H}$ = 1.3 Hz, 2H), 7.40 (td, $^3J_{H,H}$ = 7.6, $^4J_{H,H}$ = 1.5 Hz, 2H), 7.35 (td, $^3J_{H,H}$ = 7.5, $^4J_{H,H}$ = 1.4 Hz, 2H), 0.89 (s, 42H) ppm.

^{13}C NMR (151 MHz, $CDCl_3$, 25 °C) δ = 156.4, 155.9, 149.3, 148.8, 141.9, 137.8, 134.1, 129.3, 128.8, 128.2, 124.5, 122.2, 121.8, 121.4, 105.4, 95.4, 18.6, 11.3.

HRMS (ESI-ToF): calc. for $[C_{49}H_{59}N_3Si_2 + H]^+$ 746.4319; found 746.4320.

4,4''-Bis(2-ethynylphenyl)-2,2':6',2''-terpyridine (L1):

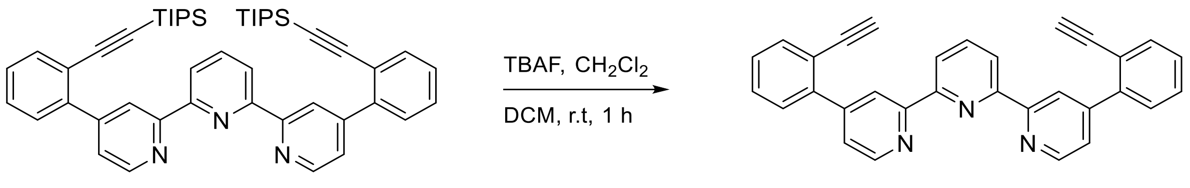

L2 **L1**

A 100 mL round-bottomed flask was set under argon atmosphere and was charged with 4,4''-bis(2-((triisopropylsilyl)ethynyl)phenyl)-2,2':6',2''-terpyridine (**L2**, 276 mg, 370 μmol, 1.0 eq). The compound was dissolved in CH_2Cl_2 (80 mL), and the solution was degassed for 15 min with an argon stream. TBAF (1 M in THF, 1.70 ml, 1.70 mmol, 4.6 eq.) was added and the mixture was stirred overnight at room temperature. After TLC showed complete conversion of the starting material, the reaction was quenched with water. CH_2Cl_2 was removed under reduced pressure and the aqueous suspension was filtered. The residue was washed with methanol and hexane several times to get rid of remaining silyl side products and other impurities. After drying under high vacuum, the product was obtained as a white solid (161 mg, 364 μmol, 100%).

Analytic data for L1:

^{1}H NMR (600 MHz, DMSO-d_6, 85 °C) δ = 8.84 (d, $^4J_{H,H}$ = 1.6 Hz, 2H), 8.79 (d, $^3J_{H,H}$ = 5.0 Hz, 2H), 8.50 (d, $^3J_{H,H}$ = 7.8 Hz, 2H), 8.14 (t, $^3J_{H,H}$ = 7.8 Hz, 1H), 7.68 – 7.64 (m, 4H), 7.61 (dd, $^3J_{H,H}$ = 7.8, $^4J_{H,H}$ = 1.4 Hz, 2H), 7.56 (td, $^3J_{H,H}$ = 7.6, $^4J_{H,H}$ = 1.4 Hz, 2H), 7.49 (td, $^3J_{H,H}$ = 7.5, $^4J_{H,H}$ = 1.4 Hz, 2H), 3.94 (s, 2H) ppm.

^{13}C NMR (151 MHz, DMSO-d_6, 85 °C): 154.6, 154.3, 148.5, 147.4, 140.2, 137.5, 133.0, 128.7, 128.5, 128.0, 123.2, 120.2, 120.1, 119.3, 83.1, 81.5 ppm.

HRMS (ESI-ToF): calc. for $[C_{31}H_{19}N_3 + H]^+$ 434.1644; found 434.1652.

Bis(4,4''-bis(2-ethynylphenyl)-2,2':6',2''-terpyridine) iron hexafluorophosphate (Fe(L1)$_2$)

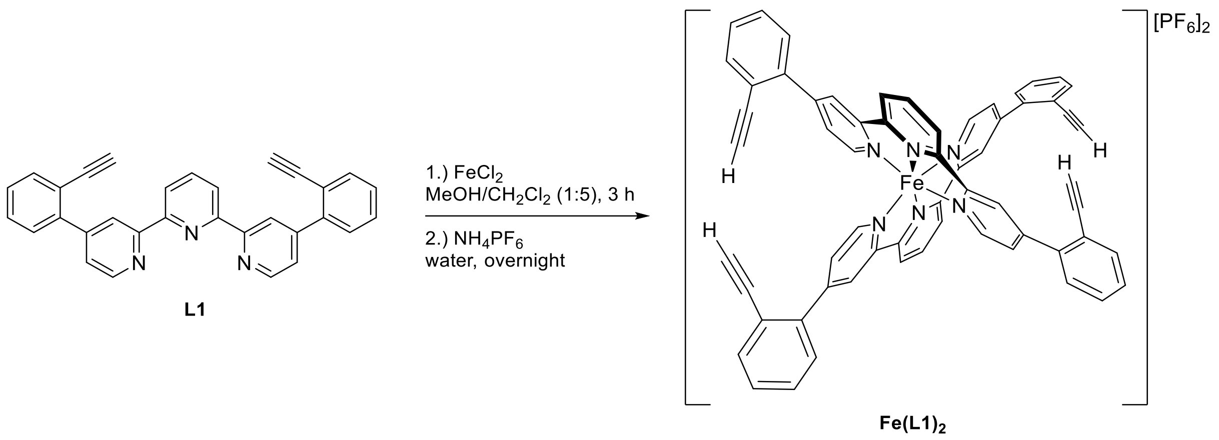

A 250 mL round-bottomed flask was charged with 4,4''-bis(2-ethynylphenyl)-2,2':6',2''-terpyridine (**L1**, 30.0 mg, 69.2 μmol, 2.0 eq.), $FeCl_2$ (8.77 mg, 69.2 μmol, 2.0 eq.), MeOH (50 mL) and CH_2Cl_2 (50 mL). The reaction mixture was stirred for 24 hours. The solvent was removed under reduced pressure. Water was added and the aqueous phase was extracted with *t*BME to remove organic impurities. Then, ammonium hexafluorophosphate (564 mg, 3.46 mmol, 100 eq.) was added in order to exchange the counter ion and make it soluble in organic compounds. The aqueous phase was extracted with CH_2Cl_2. The combined organic layers were dried over $MgSO_4$, filtered and concentrated under reduced pressure to yield the product as purple solid (39.9 mg, 33.0 μmol, 95%).

Analytic data for Fe(L1)$_2$:

^{1}H NMR (600 MHz, Acetone-*d*$_6$, 25 °C) δ = 9.42 (d, $^3J_{H,H}$ = 8.1 Hz, 4H), 9.08 (d, $^4J_{H,H}$ = 1.7 Hz, 4H), 8.93 (t, $^3J_{H,H}$ = 8.1 Hz, 2H), 7.70 – 7.65 (m, 4H), 7.62 – 7.49 (m, 20H), 3.66 (s, 4H) ppm.

^{13}C NMR (151 MHz, Acetone- *d*$_6$, 25 °C) δ = 161.4, 159.0, 153.6, 151.2, 139.5, 139.4, 135.2, 130.7, 130.7, 130.5, 128.3, 125.1, 125.0, 121.2, 84.3, 82.2 ppm.

HRMS (ESI-ToF): calc. for $[C_{62}H_{38}FeN_6]^{2+}$ 461.1249; found 461.1249.

Homocoupling of the previous formed iron complex ($Fe(L1)_2$-c):

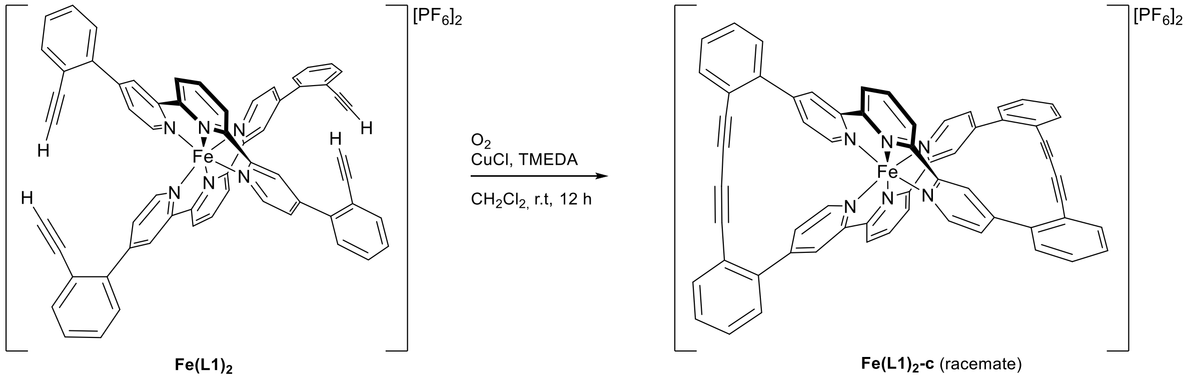

A 250 mL round-bottomed flask was charged with CH_2Cl_2 (100 mL), CuCl (11.9 mg, 116 μmol, 4.1 eq.) and TMEDA (17.7 μL, 116 μmol, 4.1 eq.). The reaction mixture was saturated with oxygen. The previously formed complex (**$Fe(L1)_2$**, 34.0 mg, 28.0 μmol, 1.0 eq.) was dissolved in CH_2Cl_2 (10 mL) and added slowly to the reaction mixture. The solution was stirred overnight under an oxygen atmosphere. After TLC control (RP, MeCN:H_2O = 9:1) indicated full conversion, the reaction was diluted with CH_2Cl_2, washed with water, dried over $MgSO_4$ and concentrated under reduced pressure. The remaining solid was sonicated with hexane and centrifuged twice to remove organic impurities to yield the desired product as purple solid (25.0 mg, 21.0 μmol, 88%).

Analytical data for $Fe(L1)_2$-c:

^{1}H NMR (600 MHz, Acetone-*d*$_6$, 25 °C) δ = 9.38 (d, $^3J_{H,H}$ = 8.1 Hz, 4H), 8.89 (t, $^3J_{H,H}$ = 8.0 Hz, 2H), 8.82 (d, $^4J_{H,H}$ = 1.7 Hz, 4H), 7.66 (dd, $^3J_{H,H}$ = 7.6, $^4J_{H,H}$ = 1.3 Hz, 4H), 7.65 – 7.61 (m, 8H), 7.57 (td, $^3J_{H,H}$ = 7.7, $^4J_{H,H}$ = 1.3 Hz, 4H), 7.49 (dd, $^3J_{H,H}$ = 7.4, $^4J_{H,H}$ = 1.2 Hz, 4H), 7.31 (dd, $^3J_{H,H}$ = 5.8, $^4J_{H,H}$ = 1.8 Hz, 4H) ppm.

^{13}C NMR (151 MHz, Acetone-d$_6$, 25 °C): δ = 161.6, 160.3, 159.6, 153.3, 152.7, 143.4, 139.7, 133.2, 131.1, 130.5, 129.3, 129.1, 125.4, 125.0, 121.1, 81.6 ppm.

HRMS (ESI-ToF): calc. for $[C_{62}H_{34}FeN_6]^{2+}$ 459.1095; found 459.1092.

Bis(4,4''-bis(2-((triisopropylsilyl)ethynyl)phenyl)-2,2':6',2''-terpyridine) ruthenium hexafluorophosphate (Ru(L2)$_2$):

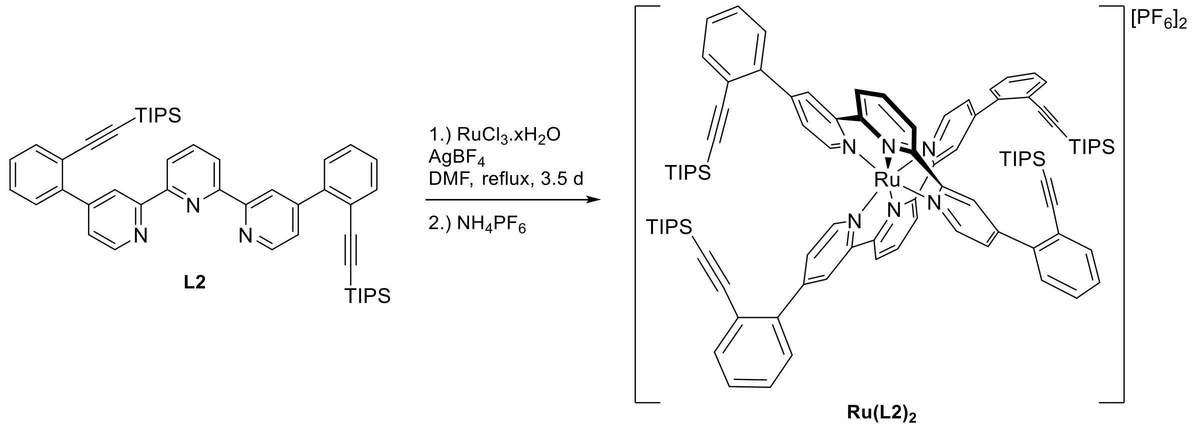

Adapted to a known literature procedure,[4] a 25 mL two-necked flask was charged with $RuCl_3.H_2O$ (14.5 mg, 7.00 μmol, 1.0 eq.), $AgBF_4$ (41.7 mg, 210 μmol, 3.0 eq.) and DMF (10 mL). The reaction mixture was heated to reflux for 3 hours, before a pale red colored precipitation appeared. Then, 4,4''-bis(2-((triisopropylsilyl)ethynyl)phenyl)-2,2':6',2''-terpyridine (**L2**, 110 mg, 147 μmol, 2.1 eq.) dissolved in DMF (5 mL) was added. The reaction mixture was refluxed for 3.5 days. Then, the reaction mixture was filtered through Celite (rinsed with DMF). The filtrate was treated with 0.5 M aqueous NH_4PF_6 (30 mL) and allowed to settle overnight. The formed precipitate was filtered over Celite, was washed several times with water and Et_2O and collected with MeCN. The crude product was purified several times by flash column chromatography (acetone to acetone/water = 10:1 to acetone/water/aq. sat. KNO_3 = 10:1:0.1) to yield the product as red solid (41.0 mg, 22.0 μmol, 31%).

Analytic data for Ru(L2)$_2$:

^{1}H NMR (400 MHz, Acetone-*d$_6$*, 22 °C) δ = 9.39 (d, $^3J_{H,H}$ = 8.2 Hz, 4H), 9.18 (d, $^4J_{H,H}$ = 1.8 Hz, 4H), 8.73 (t, $^3J_{H,H}$ = 8.1 Hz, 2H), 7.85 (dd, $^3J_{H,H}$ = 5.8, $^4J_{H,H}$ = 1.8 Hz, 4H), 7.81 (d, $^3J_{H,H}$ = 5.8 Hz, 4H), 7.74 (dd, $^3J_{H,H}$ = 7.2, $^4J_{H,H}$ = 1.5 Hz, 4H), 7.65 – 7.55 (m, 12H), 0.87 (d, $^3J_{H,H}$ = 6.0 Hz, 84H) ppm.

^{13}C NMR (101 MHz, Acetone- *d$_6$*, 22 °C) δ = 159.3, 156.6, 152.6, 151.0, 139.0, 137.3, 135.5, 130.9, 130.5, 130.5, 129.0, 125.8, 125.5, 122.3, 106.0, 96.5, 18.9, 11.8 ppm.

HRMS (ESI-ToF): calc. for $[C_{98}H_{118}N_6RuSi_4]^{2+}$ 796.3779; found 796.3766.

Bis(4,4''-bis(2-ethynylphenyl)-2,2':6',2''-terpyridine) ruthenium hexafluorophosphate ($Ru(L1)_2$):

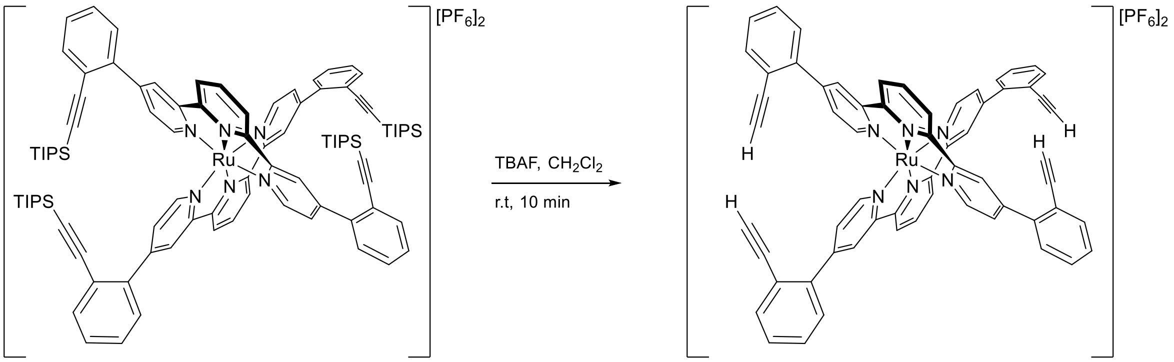

Ru(L2)2 Ru(L1)2

Ruthenium complex (**Ru(L2)₂** → **$Ru(L2)_2$**, 40.9 mg, 21.9 μmol, 1.0 eq.) was placed in a dry 100 mL round-bottomed flask and was dissolved in CH_2Cl_2 (30 mL). The solution was degassed for 15 minutes before TBAF (1 M in THF, 110 μL, 110 μmol, 5.0 eq.) was added. After 10 minutes, complete deprotection of the acetylenes was observed by MALDI-ToF analysis. The reaction was stopped by the addition of aq. sat NH_4Cl, before the organic phase was separated. The aqueous phase was extracted with CH_2Cl_2 and the combined organic layers were dried over $MgSO_4$, filtered and concentrated under reduced pressure. The residue was purified by flash column chromatography (acetone to acetone/water = 10:1 to acetone/water/aq. sat. KNO_3 = 10:1:0.1) to yield the product as red solid (27.7 mg, 22.0 μmol, 100%).

Analytic data for $Ru(L1)_2$:

^{1}H NMR (600 MHz, Acetone-*d₆*, 25 °C) δ = 9.32 (d, $^3J_{H,H}$ = 8.1 Hz, 2H), 8.91 (d, $^4J_{H,H}$ = 1.8 Hz, 2H), 8.61 (t, $^3J_{H,H}$ = 8.1 Hz, 1H), 7.88 (d, $^3J_{H,H}$ = 5.7 Hz, 2H), 7.70 – 7.54 (m, 8H), 7.42 (dd, $^3J_{H,H}$ = 5.8, $^4J_{H,H}$ = 1.8 Hz, 2H) ppm.

^{13}C NMR (151 MHz, Acetone-d_6, 25 °C) δ 159.8, 156.8, 152.0, 151.6, 143.3, 137.3, 133.2, 131.0, 130.4, 129.5, 129.30, 125.6, 125.5, 121.0, 81.5, 77.7 ppm.

HRMS (ESI-ToF): calc. for $[C_{62}H_{38}N_6Ru]^{2+}$ 484.1110; found 484.1103.

Homocoupling of the previous formed ruthenium complex (Ru(L1)$_2$-c):

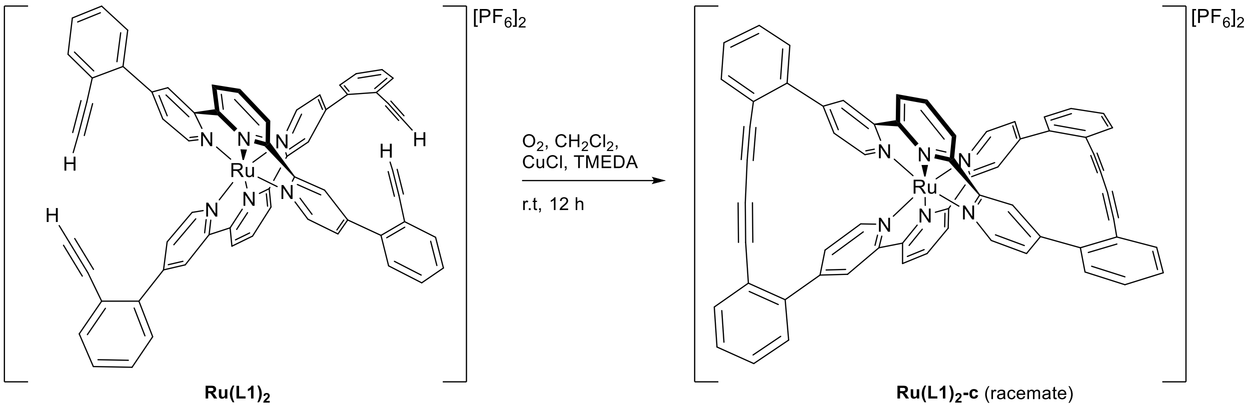

A 100 mL round-bottomed flask was filled with CH_2Cl_2 (40 mL). The solvent was saturated with oxygen and charged with CuCl (11.2 mg, 110 μmol, 5.0 eq.) and TMEDA (16.7 μL, 110 μmol, 5.0 eq.). The ruthenium complex (**Ru(L1)$_2$**, 27.7 mg, 22.0 μmol, 1.0 eq.) was dissolved in CH_2Cl_2 (15 mL) and added slowly to the reaction mixture via a dropping funnel. The reaction mixture was stirred overnight. Then the organic phase was washed with water (3 x 50 mL), dried over $MgSO_4$, filtered and concentrated under reduced pressure. The residue was purified by flash column chromatography (acetone to acetone/water = 10:1 to acetone/water/aq. sat. KNO_3 = 10:1:0.1) to yield the product as red solid (26.5 mg, 21.0 μmol, 96%).

Analytical data for Ru(L1)$_2$-c:

^{1}H NMR (600 MHz, Acetone-*d*$_6$, 25 °C) δ = 9.26 (d, $^3J_{H,H}$ = 8.2 Hz, 2H), 8.87 (dd, $^4J_{H,H}$ = 1.9, $^5J_{H,H}$ = 0.7 Hz, 2H), 8.63 (t, $^3J_{H,H}$ = 8.1 Hz, 1H), 7.89 (dd, $^3J_{H,H}$ = 5.8, $^5J_{H,H}$ = 0.7 Hz, 2H), 7.70 – 7.68 (m, 2H), 7.66 (td, $^3J_{H,H}$ = 7.6, $^4J_{H,H}$ = 1.3 Hz, 2H), 7.59 (td, $^3J_{H,H}$ = 7.7, $^4J_{H,H}$ = 1.3 Hz, 2H), 7.54 (dd, $^3J_{H,H}$ = 7.8, $^4J_{H,H}$ = 1.2 Hz, 2H), 7.44 (dd, $^3J_{H,H}$ = 5.7, $^4J_{H,H}$ = 1.8 Hz, 2H) ppm.

^{13}C NMR (151 MHz, Acetone-d$_6$, 25 °C): δ = 159.8, 156.8, 152.0, 151.6, 143.3, 137.3, 133.2, 131.0, 130.4, 129.5, 129.3, 125.6, 125.5, 121.0, 81.5, 77.7 ppm.

HRMS (ESI-ToF): calc. for $[C_{62}H_{34}N_6Ru]^{2+}$ 482.0954; found 482.0947.

^{1}H-, ^{13}C-NMR ($CDCl_3$, 400/101 MHz, 22 °C) and HR-ESI spectra of L2:

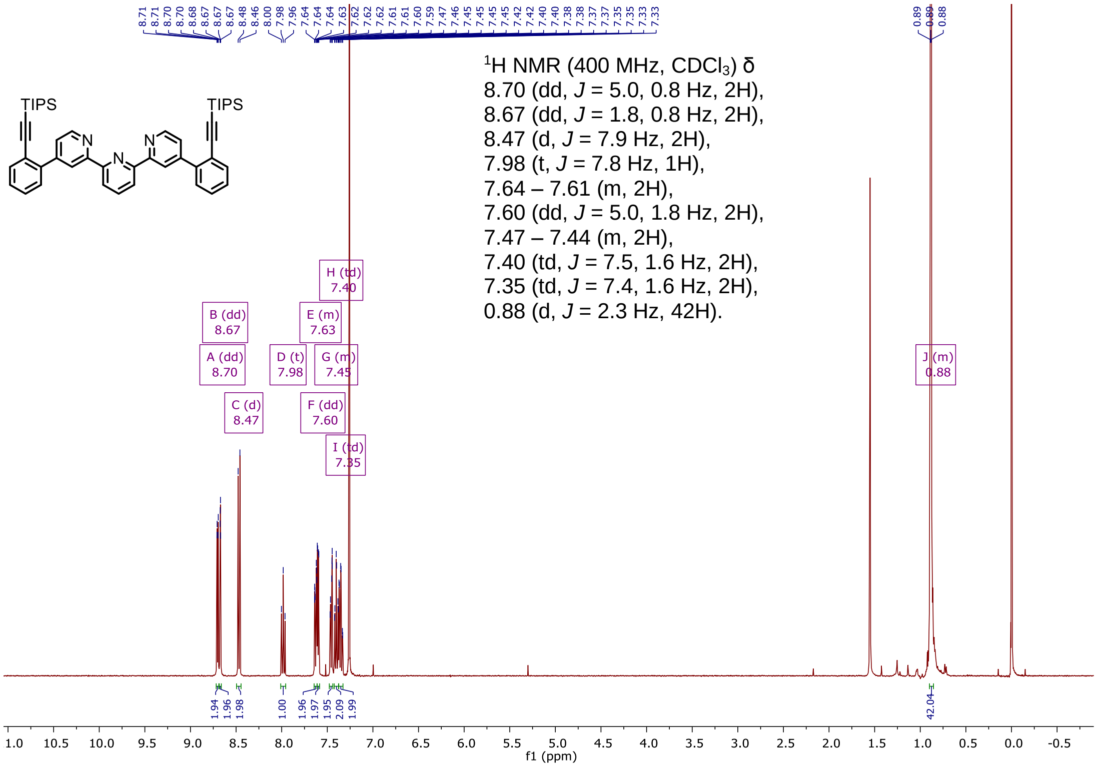

^{1}H NMR (400 MHz, $CDCl_3$) δ
8.70 (dd, J = 5.0, 0.8 Hz, 2H),
8.67 (dd, J = 1.8, 0.8 Hz, 2H),
8.47 (d, J = 7.9 Hz, 2H),
7.98 (t, J = 7.8 Hz, 1H),
7.64 – 7.61 (m, 2H),
7.60 (dd, J = 5.0, 1.8 Hz, 2H),
7.47 – 7.44 (m, 2H),
7.40 (td, J = 7.5, 1.6 Hz, 2H),
7.35 (td, J = 7.4, 1.6 Hz, 2H),
0.88 (d, J = 2.3 Hz, 42H).

^{13}C NMR (101 MHz, $CDCl_3$) δ
156.40, 155.85, 149.32, 148.83, 141.87,
137.82, 134.05, 129.25, 128.81, 128.24,
124.53, 122.16, 121.85, 121.40, 105.43,
95.42, 18.58, 11.31.

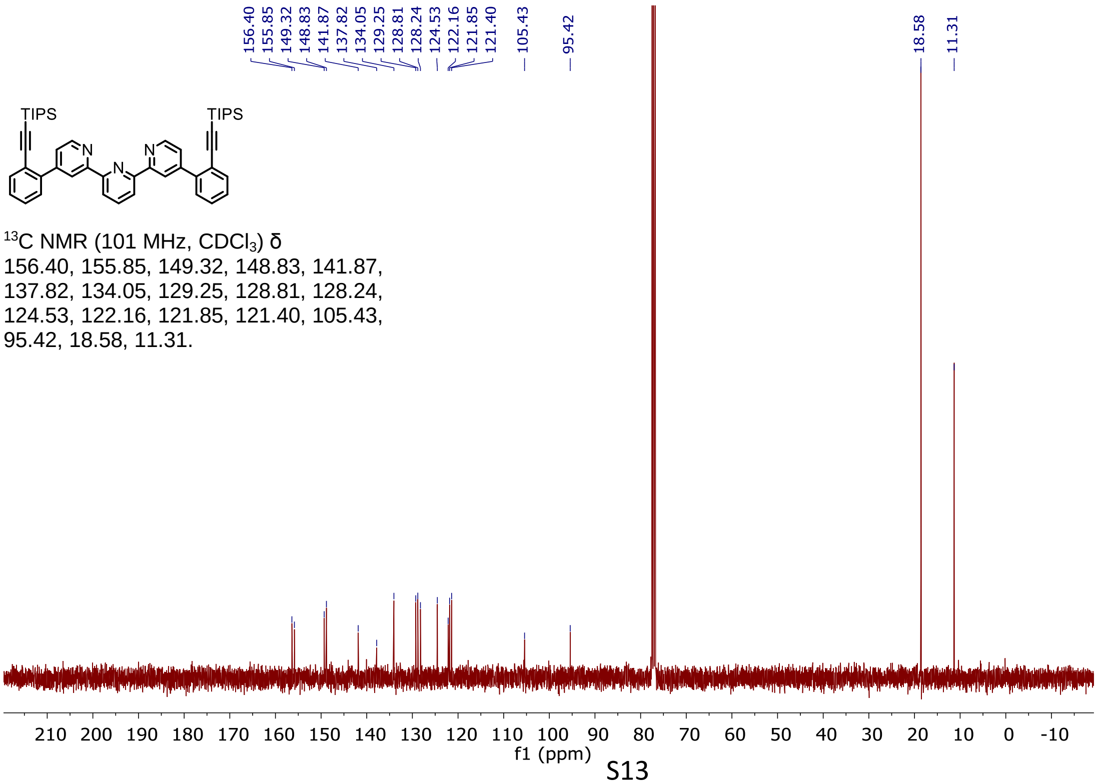

Mass Spectrum SmartFormula Report

Analysis Info

Analysis Name	N:\new acq data\VH306 ac Probe 4 001.d	Acquisition Date	16.10.2014 12:05:24
Method	hn Direct_Infusion_pos mode_75-1700 mid 4eV.m	Operator	hn
Sample Name	Viktor Hoffmann, VH306 ac Probe 4	Instrument / Ser#	maXis 4G 21243
Comment	VH 306, ca. 10 ug/ml MeOH		

Acquisition Parameter

Source Type	ESI	Ion Polarity	Positive	Set Nebulizer	0.4 Bar
Focus	Not active	Set Capillary	3600 V	Set Dry Heater	180 °C
Scan Begin	75 m/z	Set End Plate Offset	-500 V	Set Dry Gas	4.0 l/min
Scan End	1700 m/z	Set Collision Cell RF	500.0 Vpp	Set Ion Energy (MS only)	4.0 eV

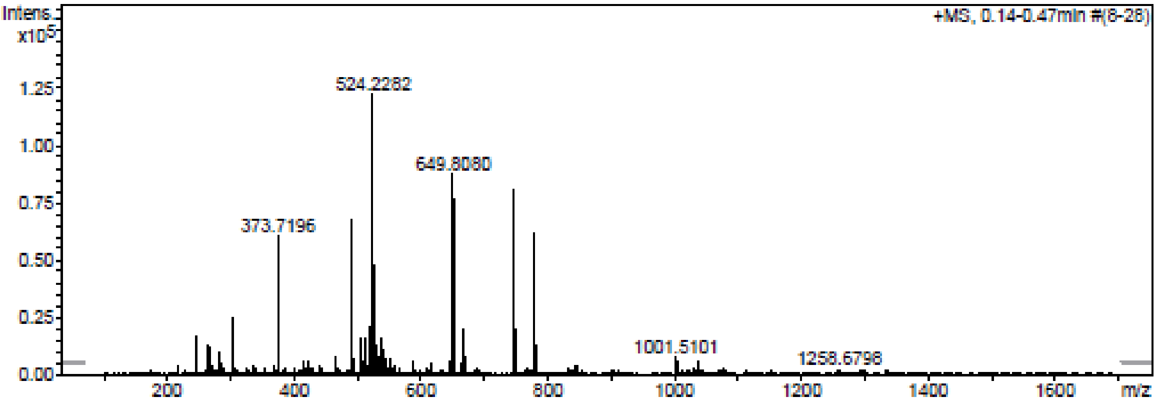

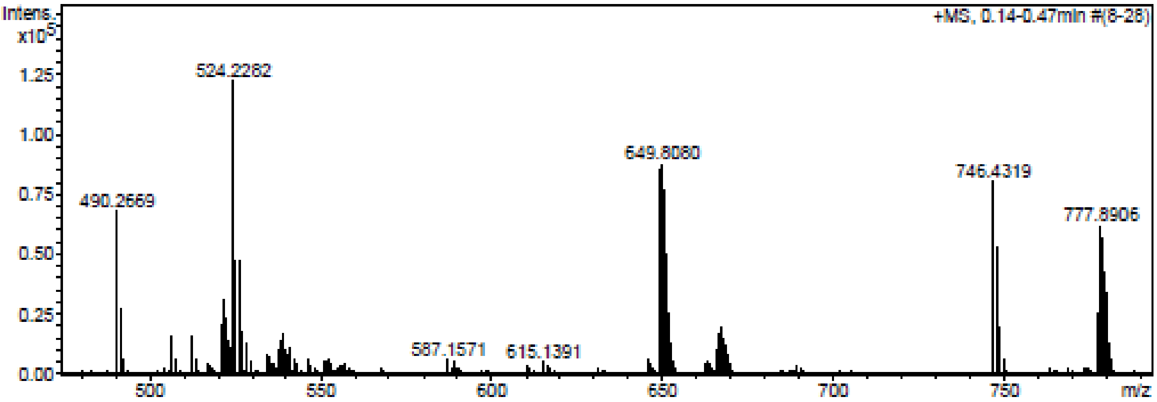

Meas. m/z	#	Formula	Score	m/z	err [mDa]	err [ppm]	mSigma	rdb	e⁻ Conf	N-Rule	z
524.2282	1	C 32 H 35 Cl N 3 Si	100.00	524.2283	0.2	0.3	25.0	17.5	even	ok	1+
746.4319	1	C 49 H 60 N 3 Si 2	100.00	746.4320	0.1	0.1	14.0	23.5	even	ok	

^{1}H-, ^{13}C-NMR ($CDCl_3$, 600/151 MHz, 25 °C) and HR-ESI spectra of L1:

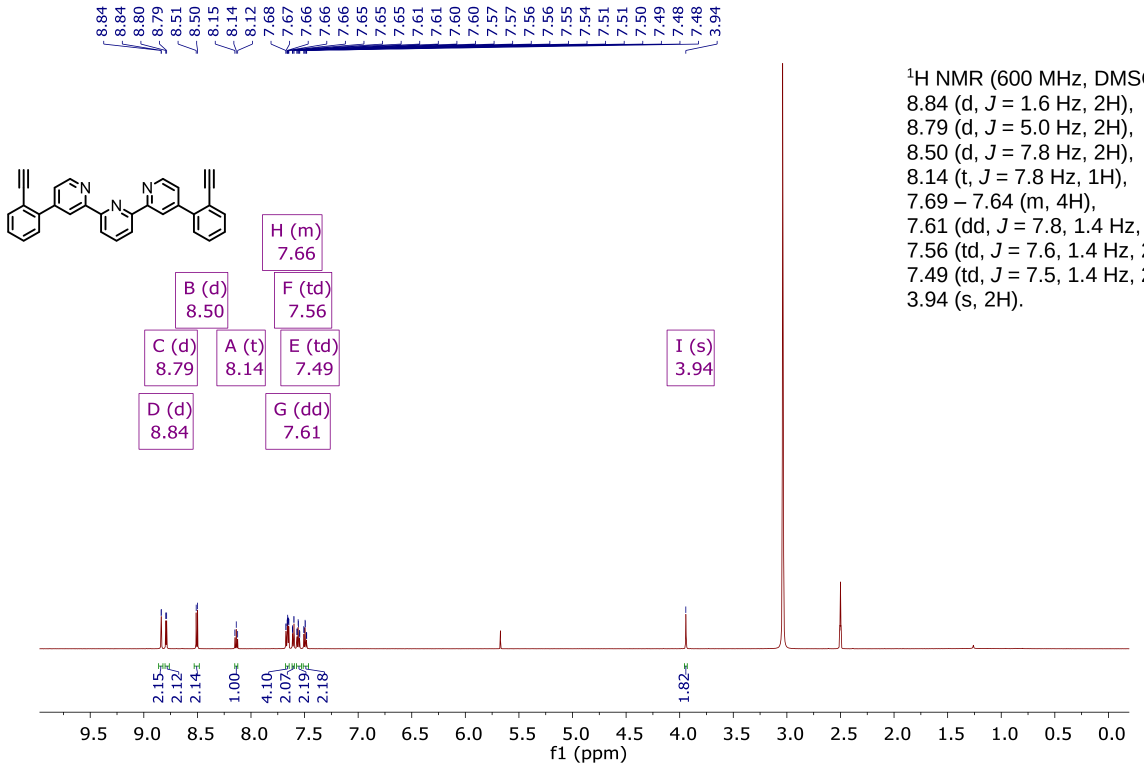

^{1}H NMR (600 MHz, DMSO-d_6) δ 8.84 (d, J = 1.6 Hz, 2H), 8.79 (d, J = 5.0 Hz, 2H), 8.50 (d, J = 7.8 Hz, 2H), 8.14 (t, J = 7.8 Hz, 1H), 7.69 – 7.64 (m, 4H), 7.61 (dd, J = 7.8, 1.4 Hz, 2H), 7.56 (td, J = 7.6, 1.4 Hz, 2H), 7.49 (td, J = 7.5, 1.4 Hz, 2H), 3.94 (s, 2H).

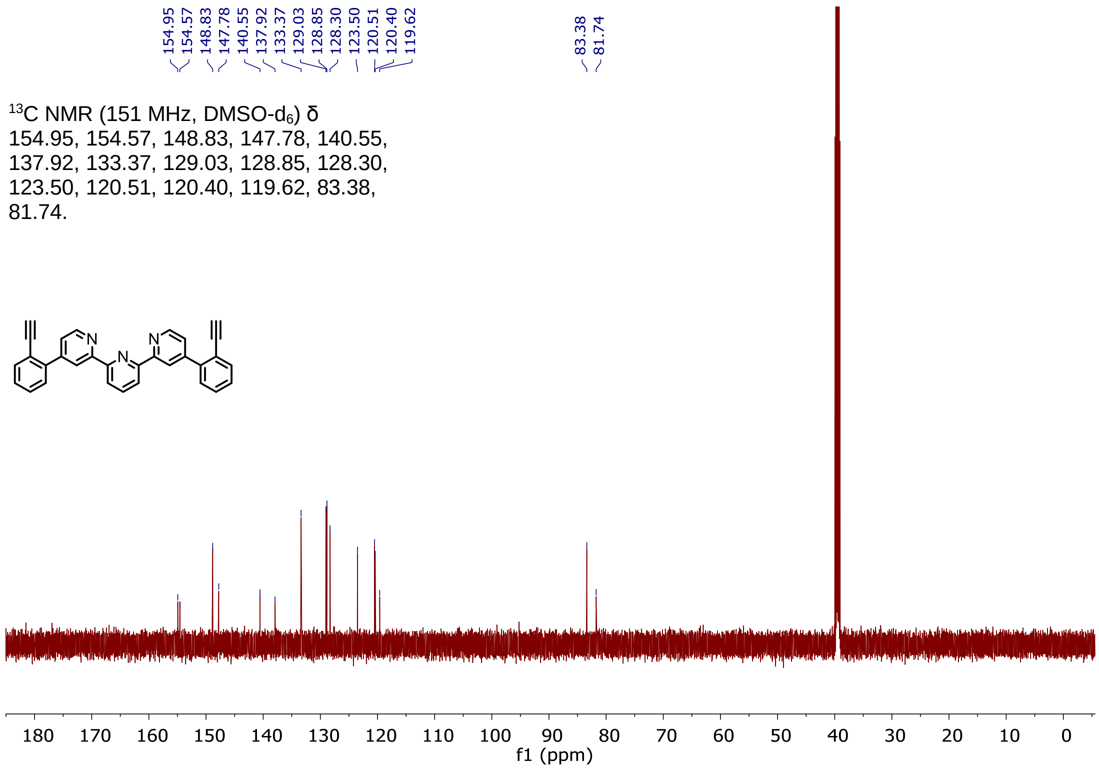

^{13}C NMR (151 MHz, DMSO-d$_6$) δ 154.95, 154.57, 148.83, 147.78, 140.55, 137.92, 133.37, 129.03, 128.85, 128.30, 123.50, 120.51, 120.40, 119.62, 83.38, 81.74.

Mass Spectrum SmartFormula Report

Analysis Info

Analysis Name	N:\new acq data\VH314 cr 001.d	Acquisition Date	16.10.2014 14:19:35
Method	hn Direct_Infusion_pos mode_75-1700 mid 4eV.m	Operator	hn
Sample Name	Viktor Hoffmann, VH314 cr	Instrument / Ser#	maXis 4G 21243
Comment	VH 314 cr, ca. 10 ug/ml MeCN		

Acquisition Parameter

Source Type	ESI	Ion Polarity	Positive	Set Nebulizer	0.4 Bar
Focus	Not active	Set Capillary	3600 V	Set Dry Heater	180 °C
Scan Begin	75 m/z	Set End Plate Offset	-500 V	Set Dry Gas	4.0 l/min
Scan End	1700 m/z	Set Collision Cell RF	500.0 Vpp	Set Ion Energy (MS only)	4.0 eV

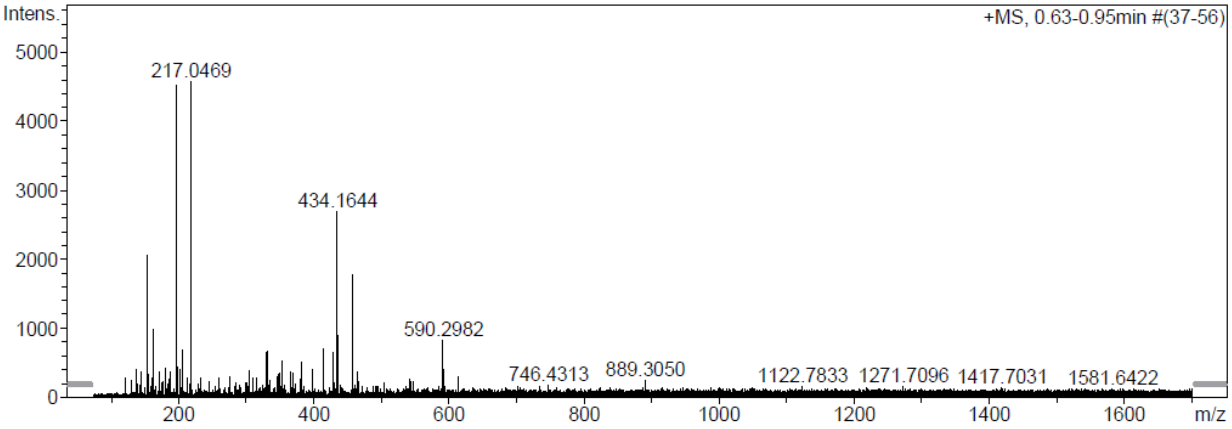

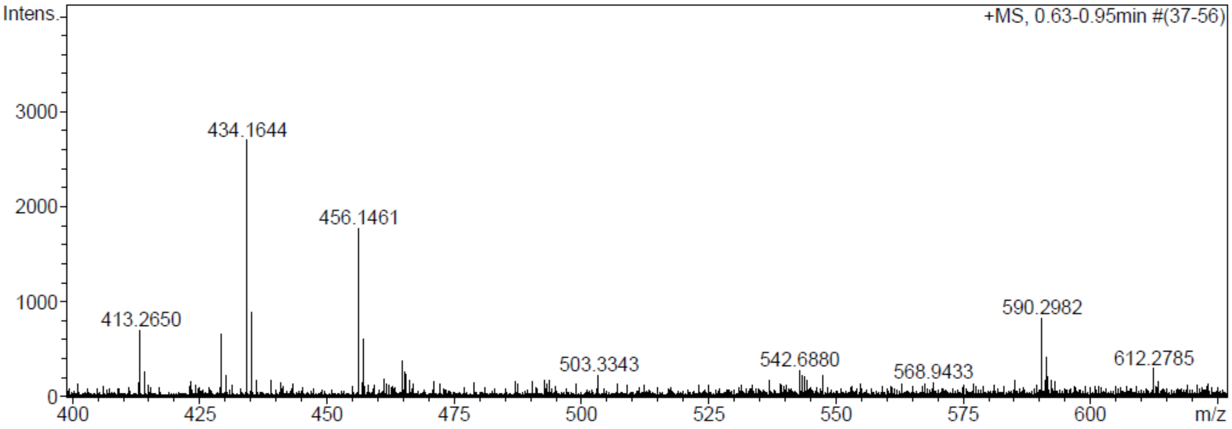

Meas. m/z	#	Formula	Score	m/z	err [mDa]	err [ppm]	mSigma	rdb	e⁻ Conf	N-Rule	z
434.1644	1	C 31 H 20 N 3	100.00	434.1652	0.7	1.7	9.9	23.5	even	ok	1+
590.2982	1	C 40 H 40 N 3 Si	100.00	590.2986	0.4	0.6	32.0	23.5	even	ok	

^{1}H-, ^{13}C-NMR ($CDCl_3$, 600/151 MHz, 25 °C) and HR-ESI spectra of $Fe(L1)_2$:

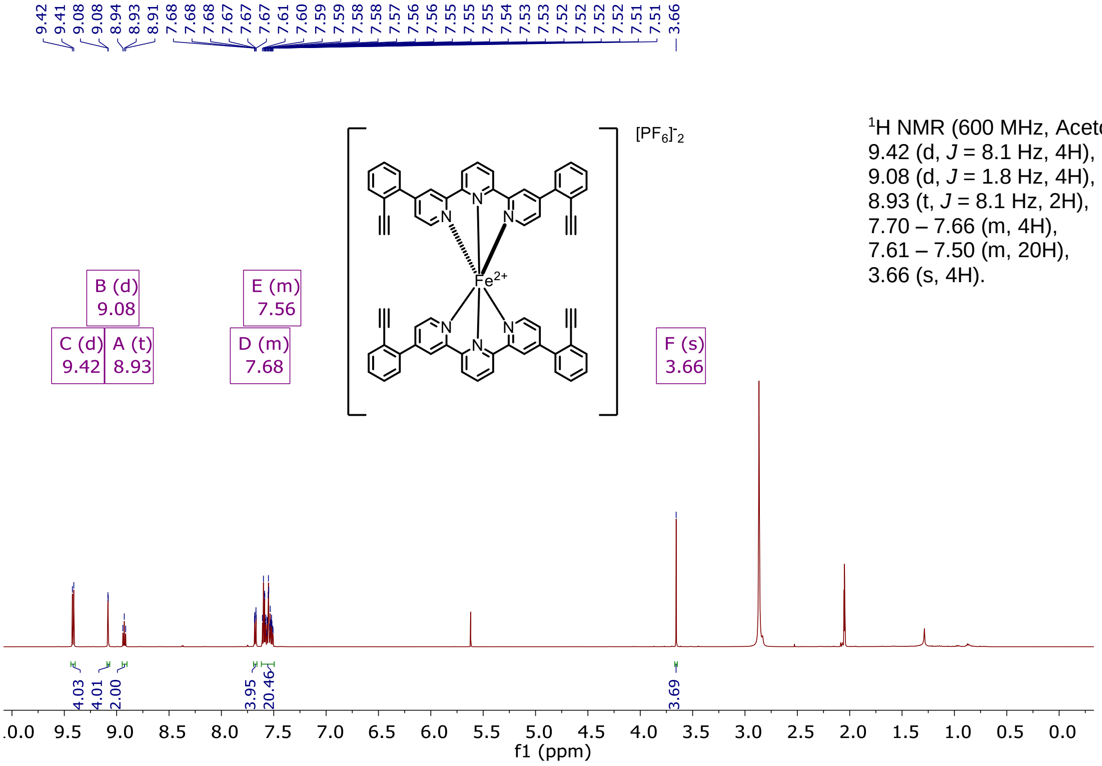

^{1}H NMR (600 MHz, Acetone-d_6) δ
9.42 (d, J = 8.1 Hz, 4H),
9.08 (d, J = 1.8 Hz, 4H),
8.93 (t, J = 8.1 Hz, 2H),
7.70 – 7.66 (m, 4H),
7.61 – 7.50 (m, 20H),
3.66 (s, 4H).

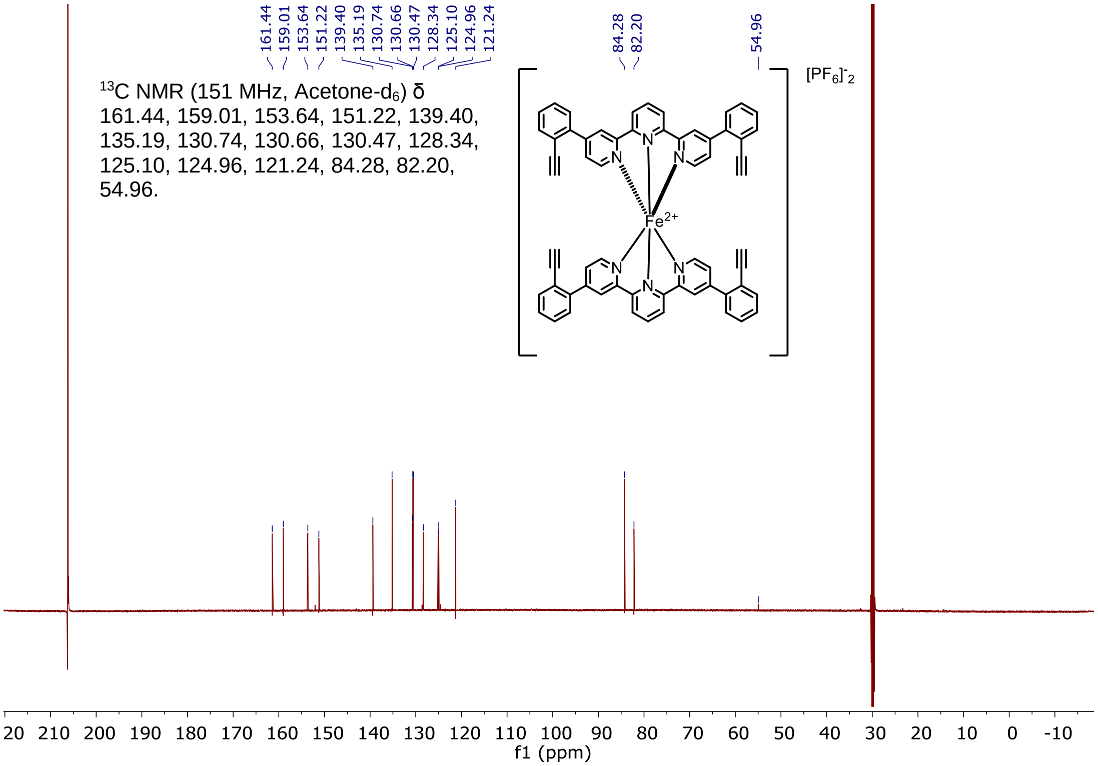

^{13}C NMR (151 MHz, Acetone-d_6) δ
161.44, 159.01, 153.64, 151.22, 139.40,
135.19, 130.74, 130.66, 130.47, 128.34,
125.10, 124.96, 121.24, 84.28, 82.20,
54.96.

Mass Spectrum SmartFormula Report

Analysis Info

Analysis Name	N:\new acq data\VH-323 003.d	Acquisition Date	23.10.2014 15:49:29
Method	hn Direct_Infusion_pos mode_75-1700 mid 4eV.m	Operator	hn
Sample Name	Viktor Hoffmann, VH-323	Instrument / Ser#	maXis 4G 21243
Comment	VH-323, ca. 10 ug/ml MeOH		

Acquisition Parameter

Source Type	ESI	Ion Polarity	Positive	Set Nebulizer	0.4 Bar
Focus	Not active	Set Capillary	3600 V	Set Dry Heater	180 °C
Scan Begin	75 m/z	Set End Plate Offset	-500 V	Set Dry Gas	4.0 l/min
Scan End	1700 m/z	Set Collision Cell RF	500.0 Vpp	Set Ion Energy (MS only)	4.0 eV

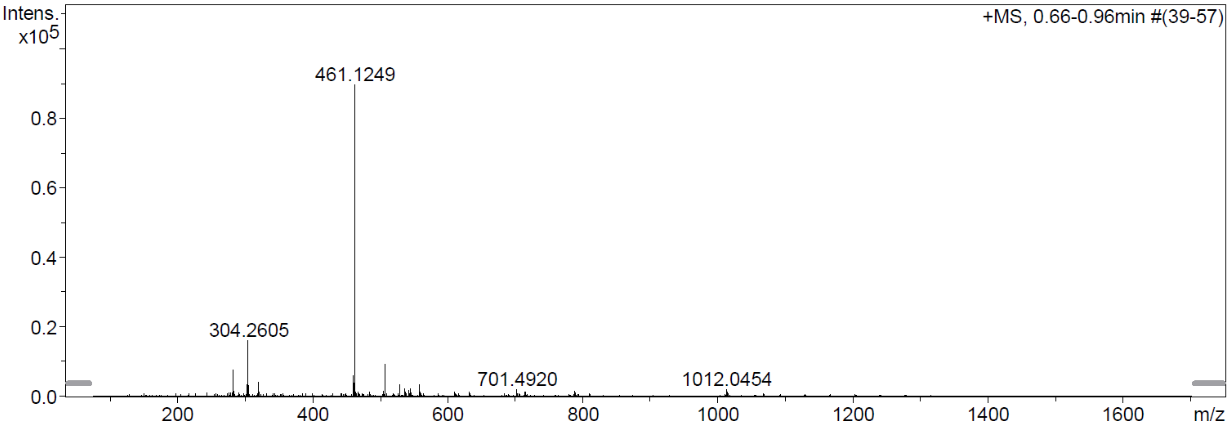

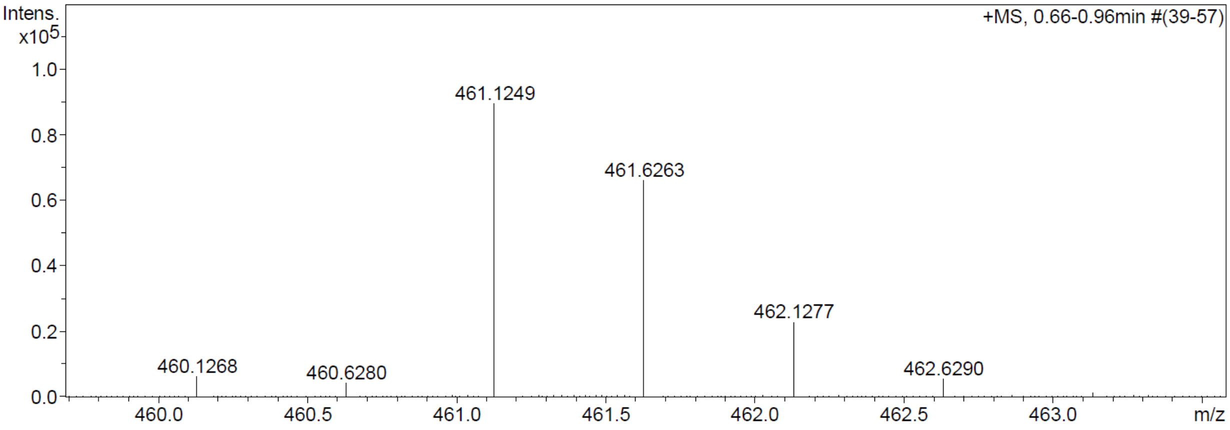

Meas. m/z	#	Formula	Score	m/z	err [mDa]	err [ppm]	mSigma	rdb	e⁻ Conf	N-Rule	z
461.1249	1	C 62 H 38 Fe N 6	100.00	461.1249	-0.0	-0.1	9.6	47.0	even	ok	2+

^{1}H-, ^{13}C-NMR, COSY, NOESY, HMBC, HMQC ($CDCl_3$, 600/101/150 MHz, 25 °C) and HR-ESI spectra of $Fe(L1)_2$-c with full assignment:

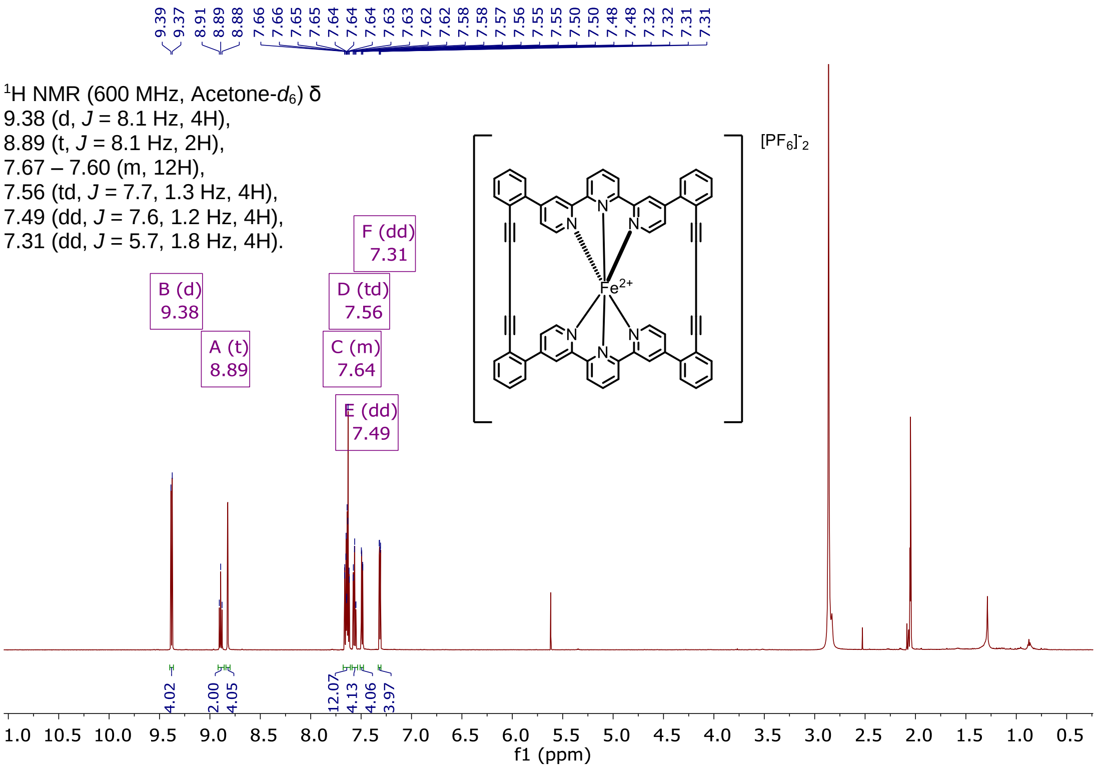

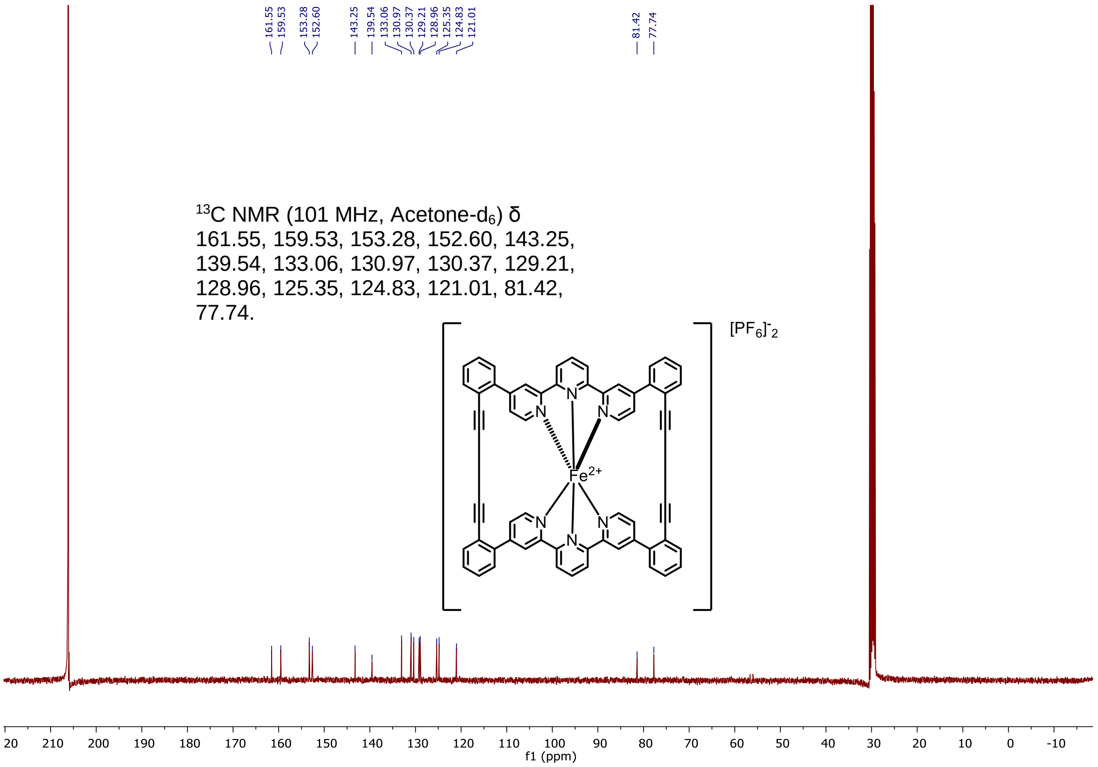

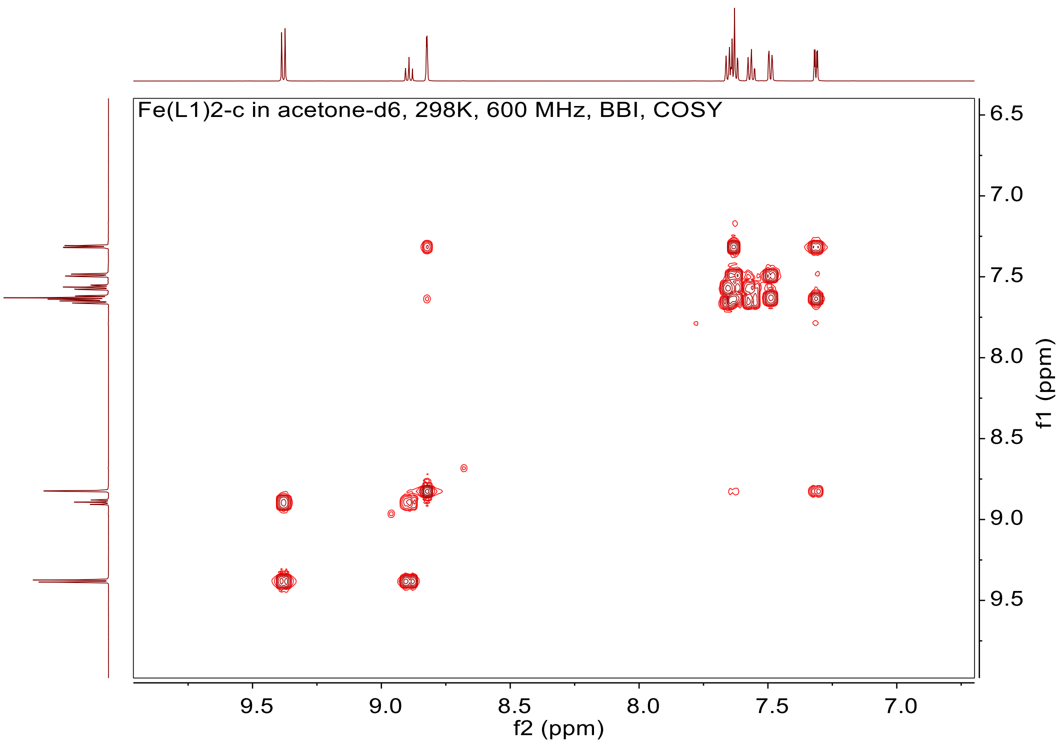
Fe(L1)2-c in acetone-d6, 298K, 600 MHz, BBI, COSY
f1 (ppm)
6.5
7.0
7.5
8.0
8.5
9.0
9.5
f2 (ppm)
9.5
9.0
8.5
8.0
7.5
7.0

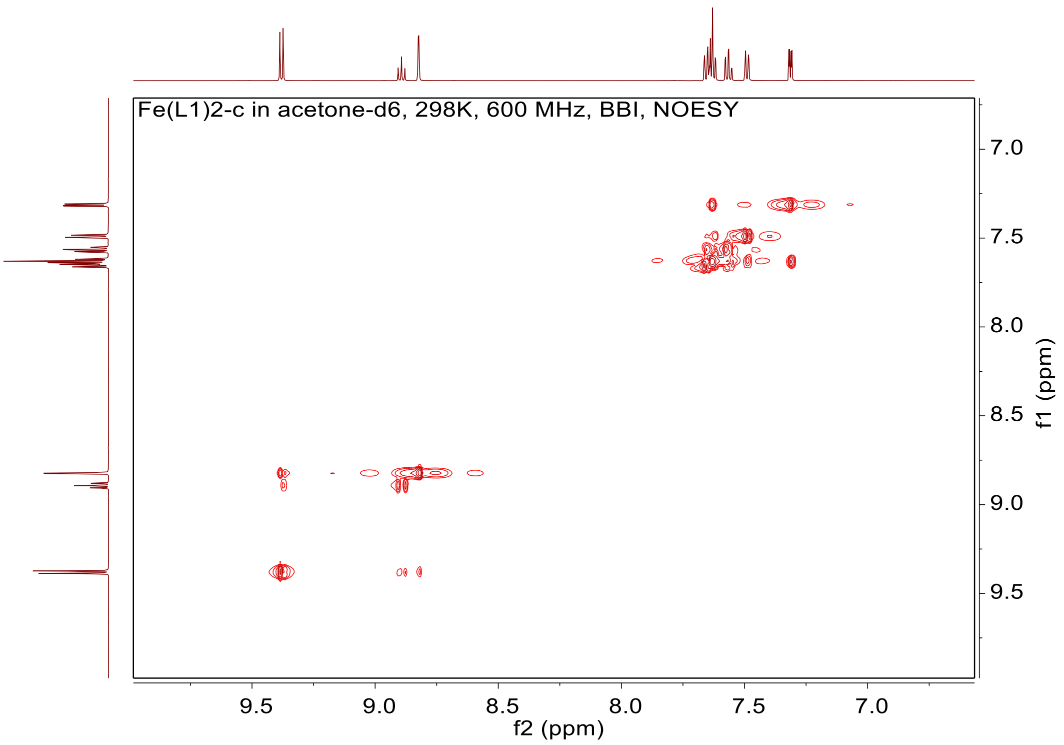
Fe(L1)2-c in acetone-d6, 298K, 600 MHz, BBI, NOESY
f1 (ppm)
7.0
7.5
8.0
8.5
9.0
9.5
f2 (ppm)
9.5
9.0
8.5
8.0
7.5
7.0

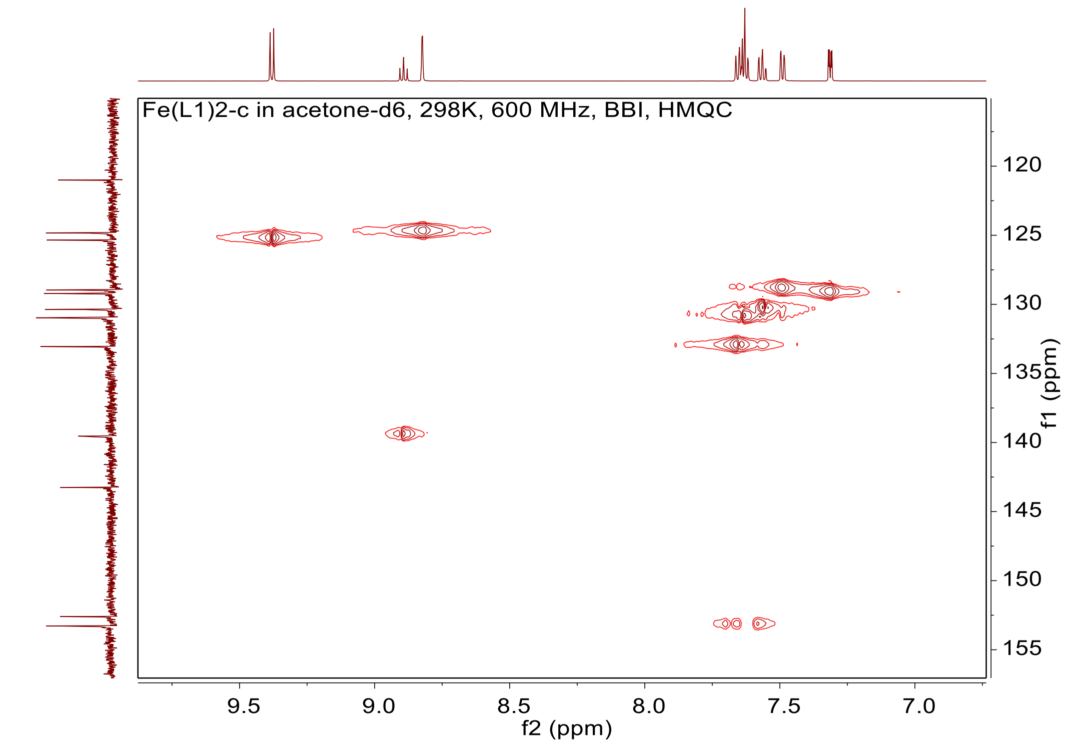
Fe(L1)2-c in acetone-d6, 298K, 600 MHz, BBI, HMQC
120
125
130
135
140
145
150
155
f1 (ppm)
9.5
9.0
8.5
8.0
7.5
7.0
f2 (ppm)

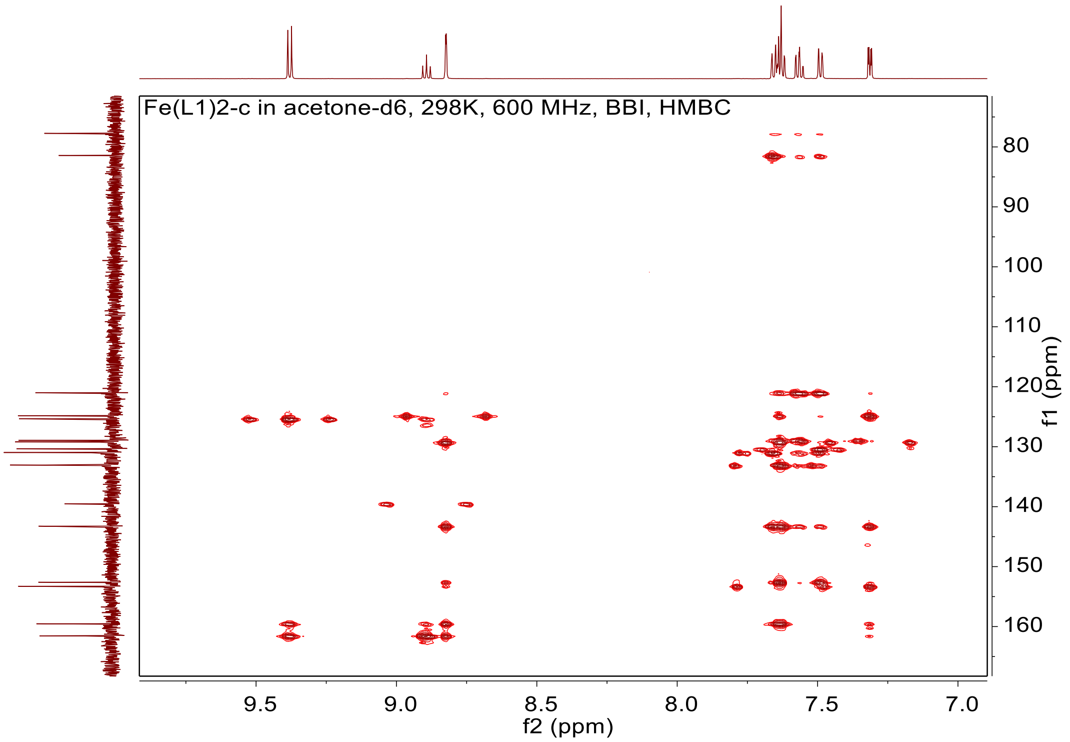
Fe(L1)2-c in acetone-d6, 298K, 600 MHz, BBI, HMBC
80
90
100
110
120
130
140
150
160
f1 (ppm)
9.5
9.0
8.5
8.0
7.5
7.0
f2 (ppm)

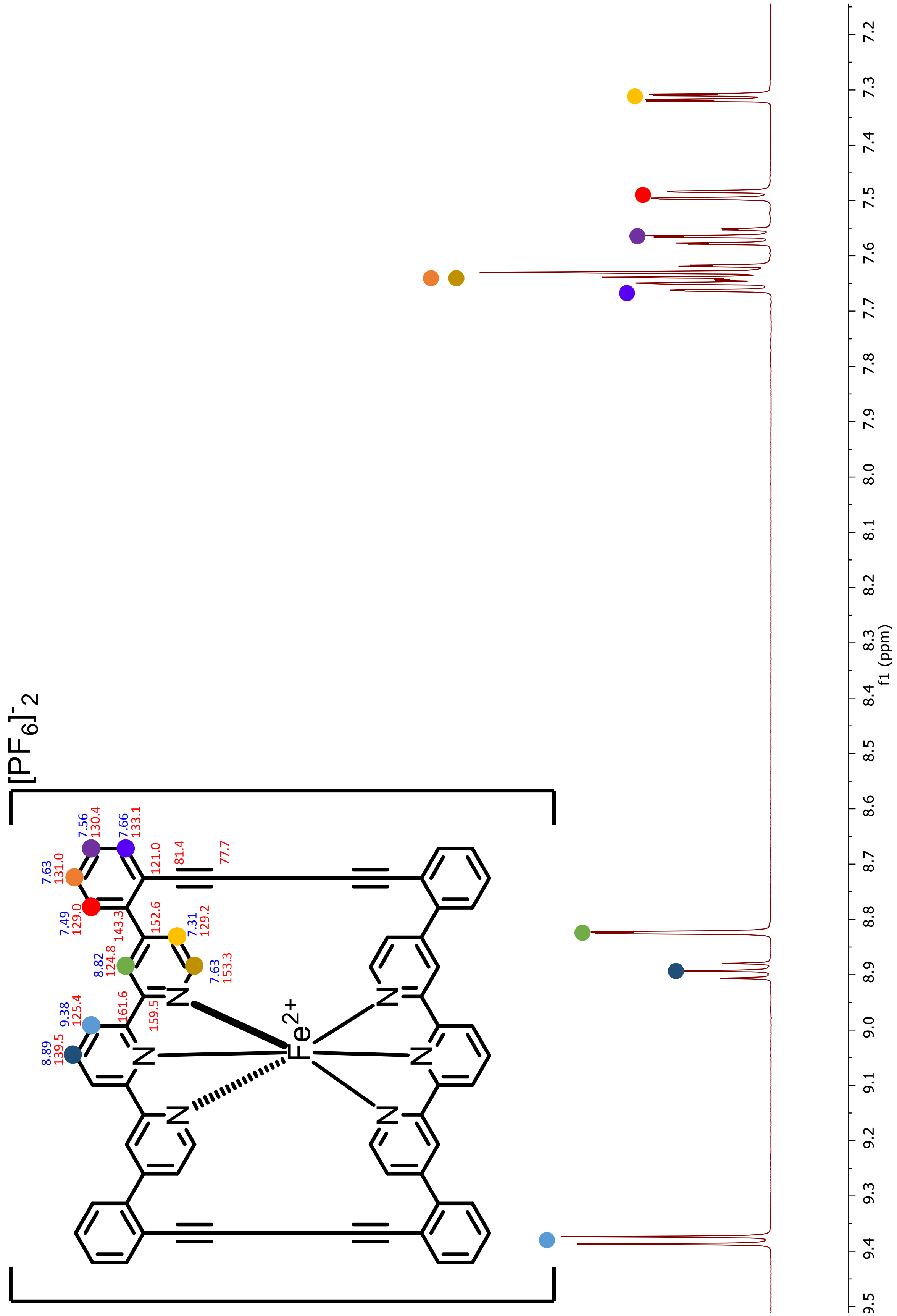
[PF6]-2
Fe2+
8.89
139.5
9.38
125.4
161.6
159.5
8.82
124.8
143.3
152.6
7.31
129.2
7.63
153.3
7.49
129.0
7.63
131.0
7.56
130.4
7.66
133.1
121.0
81.4
77.7
9.5
9.4
9.3
9.2
9.1
9.0
8.9
8.8
8.7
8.6
8.5
8.4
8.3
8.2
8.1
8.0
7.9
7.8
7.7
7.6
7.5
7.4
7.3
7.2
f1 (ppm)

Mass Spectrum SmartFormula Report

Analysis Info

Analysis Name	N:\new acq data\VH326 001.d	Acquisition Date	24.10.2014 18:15:19
Method	hn Direct_Infusion_pos mode_75-1700 mid 4eV.m	Operator	hn
Sample Name	Viktor Hoffmann, VH326	Instrument / Ser#	maXis 4G 21243
Comment	VH326, ca 10 ug/ml MeOH		

Acquisition Parameter

Source Type	ESI	Ion Polarity	Positive	Set Nebulizer	0.4 Bar
Focus	Not active	Set Capillary	3600 V	Set Dry Heater	180 °C
Scan Begin	75 m/z	Set End Plate Offset	-500 V	Set Dry Gas	4.0 l/min
Scan End	1700 m/z	Set Collision Cell RF	500.0 Vpp	Set Ion Energy (MS only)	4.0 eV

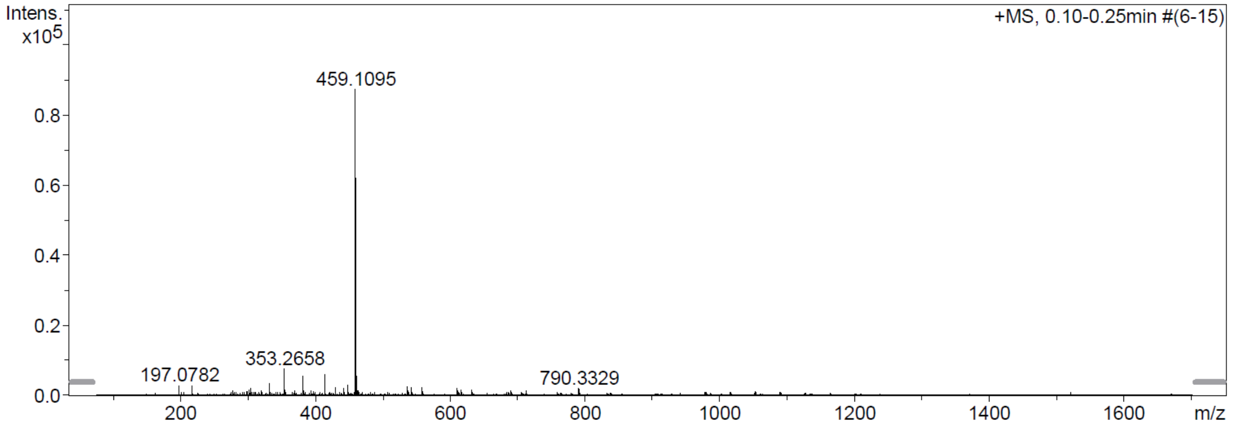

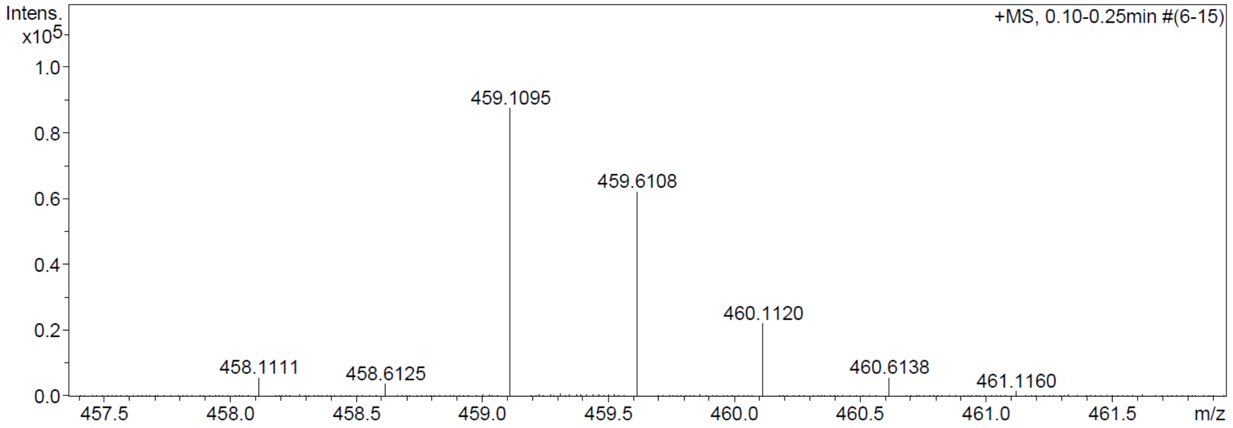

Meas. m/z	#	Formula	Score	m/z	err [mDa]	err [ppm]	mSigma	rdb	e⁻ Conf	N-Rule	z
459.1095	1	C 62 H 34 Fe N 6	100.00	459.1092	-0.2	-0.5	2.5	49.0	even	ok	2+

^{1}H-, ^{13}C-NMR ($CDCl_3$, 400/101 MHz, 22 °C) and HR-ESI spectra of Ru(L2)$_2$:

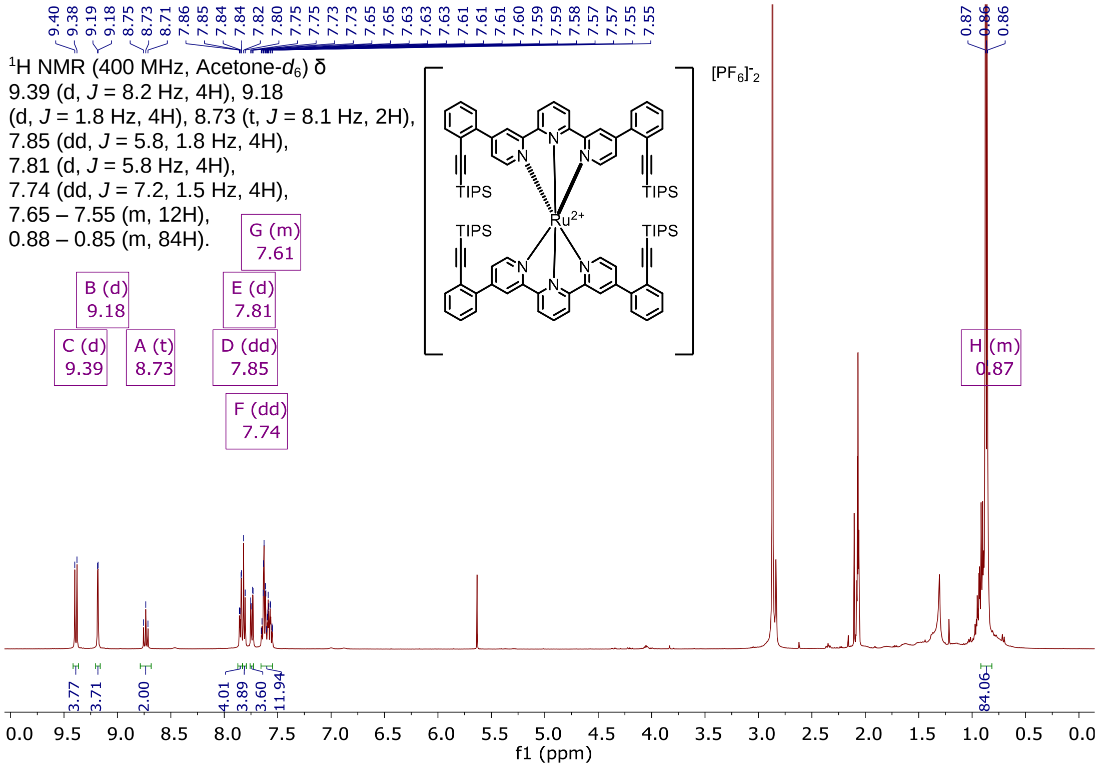

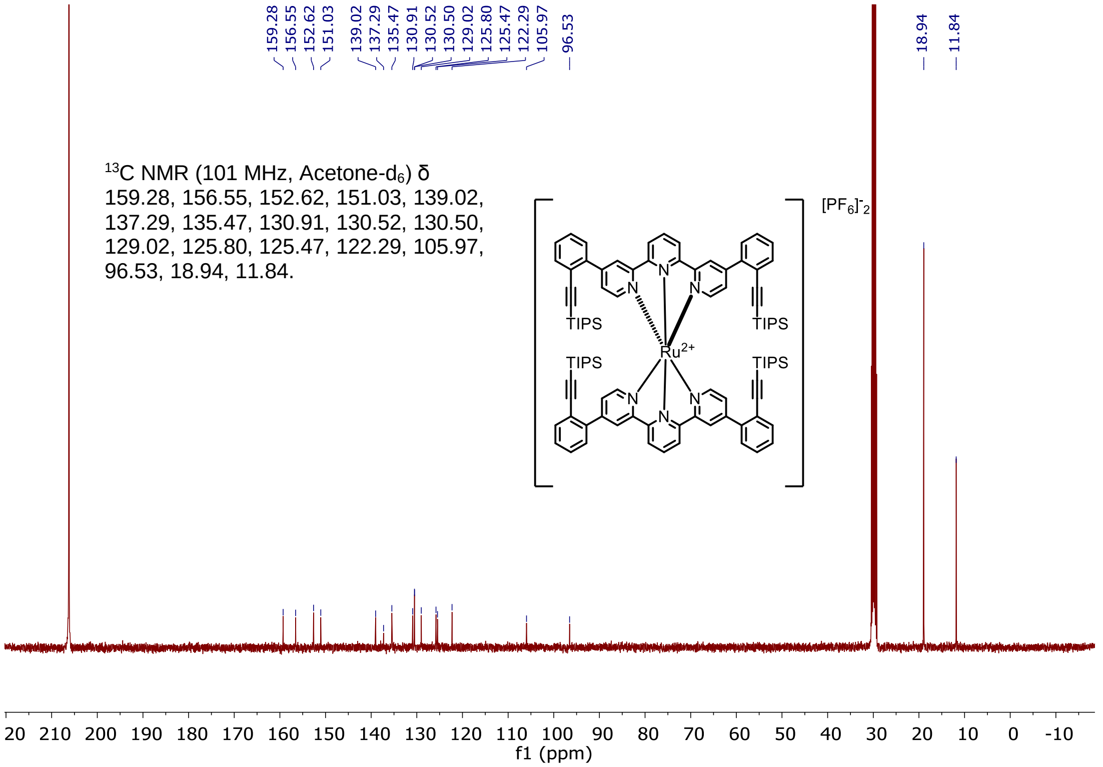

Mass Spectrum SmartFormula Report

Analysis Info

Analysis Name	N:\new acq data\BRT132 001.d	Acquisition Date	08.12.2015 15:49:07
Method	hn Direct_Infusion_pos mode_75-2000 high 4eV.m	Operator	hn
Sample Name	Thomas Brandl, BRT132	Instrument / Ser#	maXis 4G 21243
Comment	ca. 20 ug/mL MeCN mit DCM		

Acquisition Parameter

Source Type	ESI	Ion Polarity	Positive	Set Nebulizer	0.4 Bar
Focus	Not active	Set Capillary	3600 V	Set Dry Heater	180 °C
Scan Begin	75 m/z	Set End Plate Offset	-500 V	Set Dry Gas	4.0 l/min
Scan End	2000 m/z	Set Collision Cell RF	1000.0 Vpp	Set Ion Energy (MS only)	4.0 eV

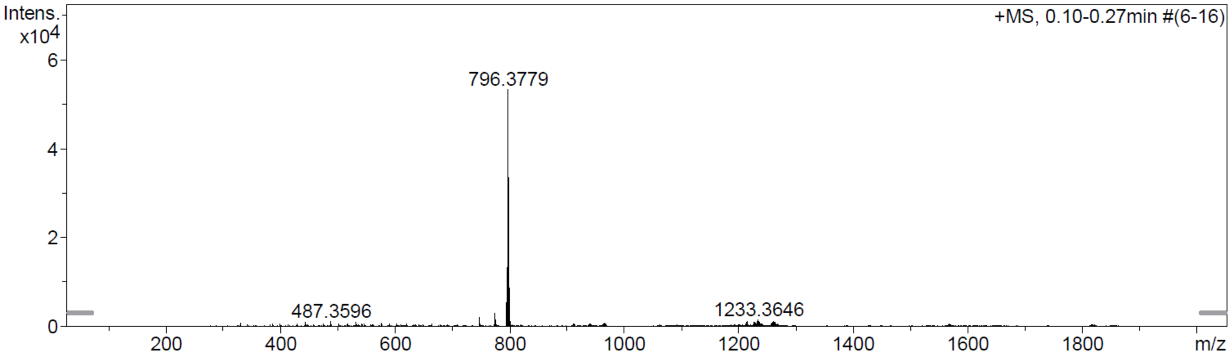

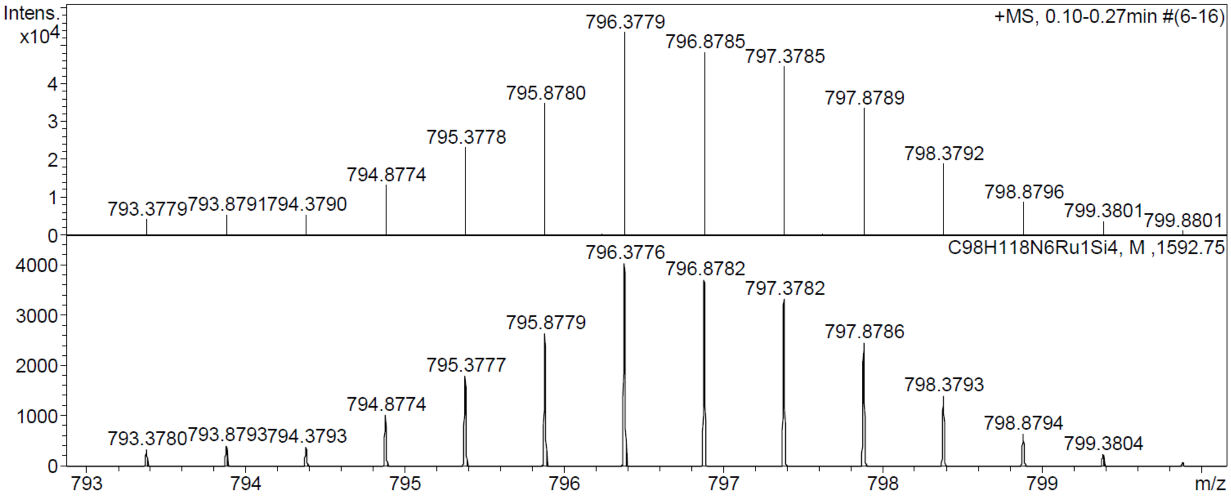

Meas. m/z	#	Formula	Score	m/z	err [mDa]	err [ppm]	mSigma	rdb	e⁻ Conf	N-Rule	z
796.3779	1	C 98 H 118 N 6 Ru Si 4	100.00	796.3766	-1.3	-1.7	7.4	47.5	odd	-	2+

1H-, 13C-NMR (CDCl3, 600/151 MHz, 25 °C) and HR-ESI spectra of Ru(L1)2:

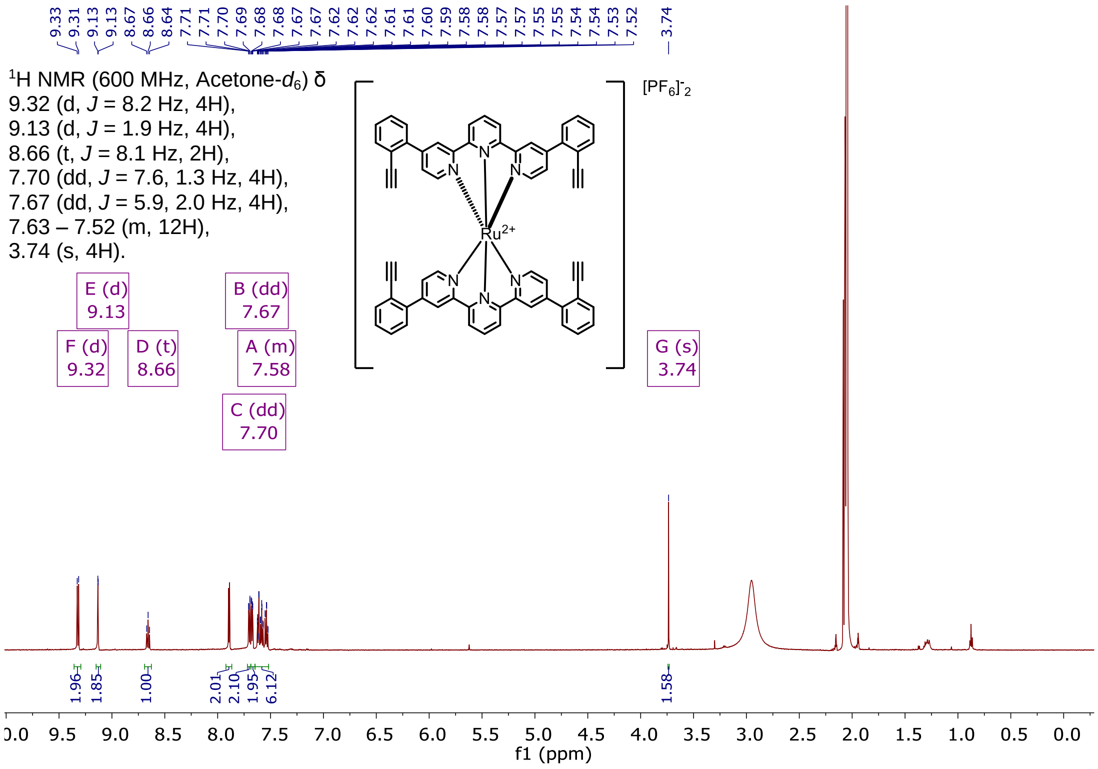

^{1}H NMR (600 MHz, Acetone-d_6) δ 9.32 (d, J = 8.2 Hz, 4H), 9.13 (d, J = 1.9 Hz, 4H), 8.66 (t, J = 8.1 Hz, 2H), 7.70 (dd, J = 7.6, 1.3 Hz, 4H), 7.67 (dd, J = 5.9, 2.0 Hz, 4H), 7.63 – 7.52 (m, 12H), 3.74 (s, 4H).

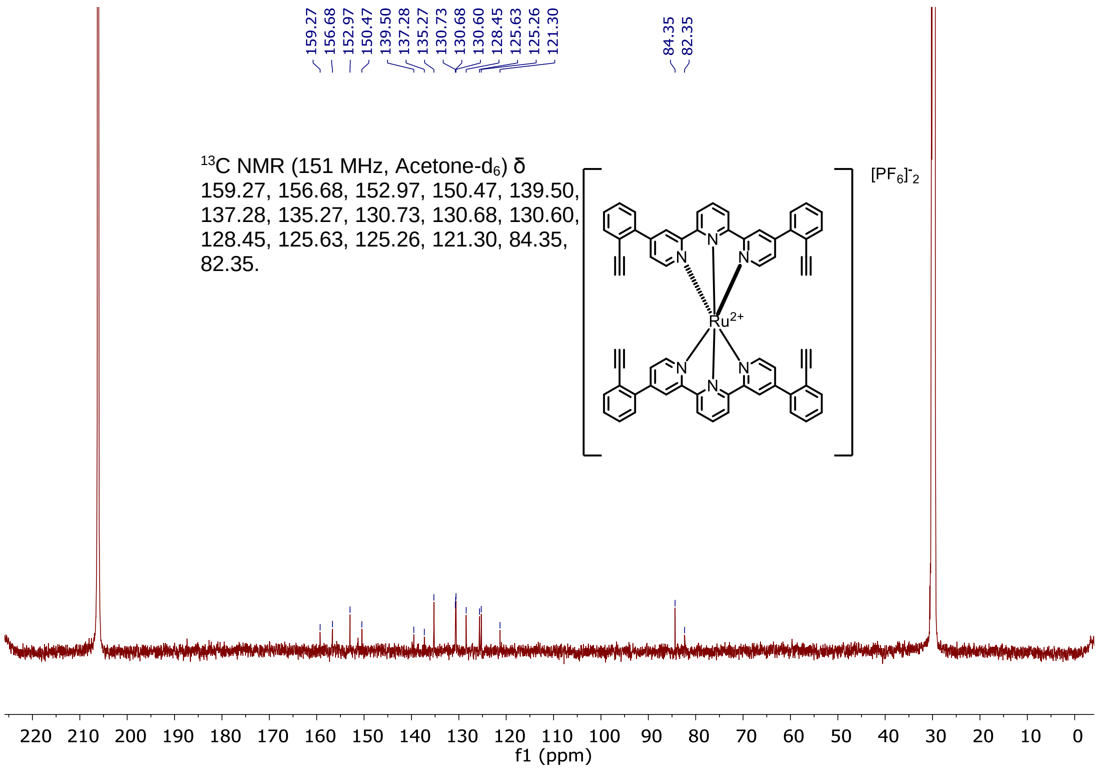

^{13}C NMR (151 MHz, Acetone-d_6) δ 159.27, 156.68, 152.97, 150.47, 139.50, 137.28, 135.27, 130.73, 130.68, 130.60, 128.45, 125.63, 125.26, 121.30, 84.35, 82.35.

Mass Spectrum SmartFormula Report

Analysis Info

Analysis Name	N:\new acq data\BRT119 002.d	Acquisition Date	13.11.2015 15:34:44
Method	hn Direct_Infusion_pos mode_75-1700 mid 4eV.m	Operator	hn
Sample Name	Thomas Brandl	Instrument / Ser#	maXis 4G 21243
Comment	BRT119, ca. 10 ug/ml MeCN, 1:3 verdünnt		

Acquisition Parameter

Source Type	ESI	Ion Polarity	Positive	Set Nebulizer	0.4 Bar
Focus	Not active	Set Capillary	3600 V	Set Dry Heater	180 °C
Scan Begin	75 m/z	Set End Plate Offset	-500 V	Set Dry Gas	4.0 l/min
Scan End	1700 m/z	Set Collision Cell RF	350.0 Vpp	Set Ion Energy (MS only)	4.0 eV

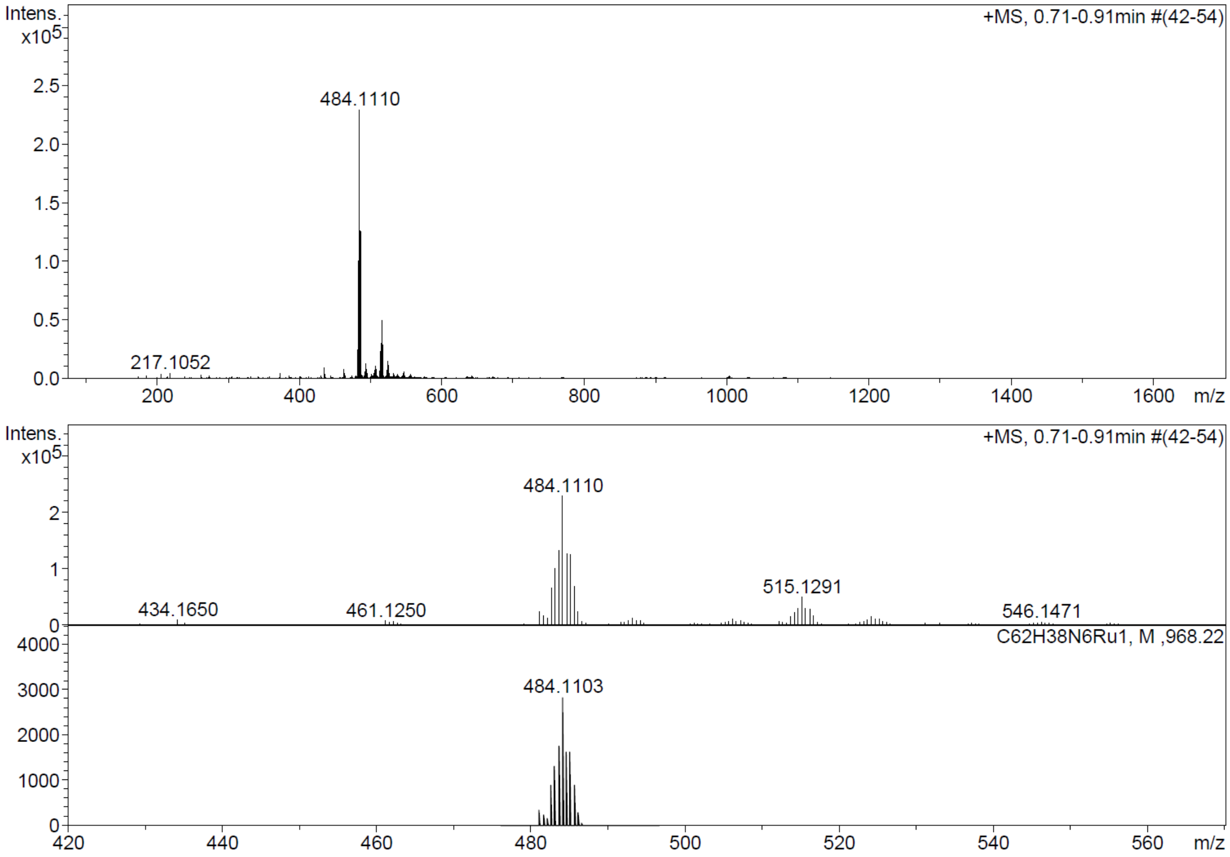

Meas. m/z	#	Formula	Score	m/z	err [mDa]	err [ppm]	mSigma	rdb	e⁻ Conf	N-Rule	z
484.1110	1	C 62 H 38 N 6 Ru	100.00	484.1103	-0.7	-1.4	18.6	47.5	odd	-	2+
515.1291	1	C 64 H 44 N 6 O 2 Ru	100.00	515.1288	-0.4	-0.7	7.1	46.5	odd	-	

^{1}H-, ^{13}C-NMR, COSY, NOESY, HMBC, HMQC ($CDCl_3$, 600/151 MHz, 25 °C) and HR-ESI spectra of Ru(L1)$_2$-c with full assignment:

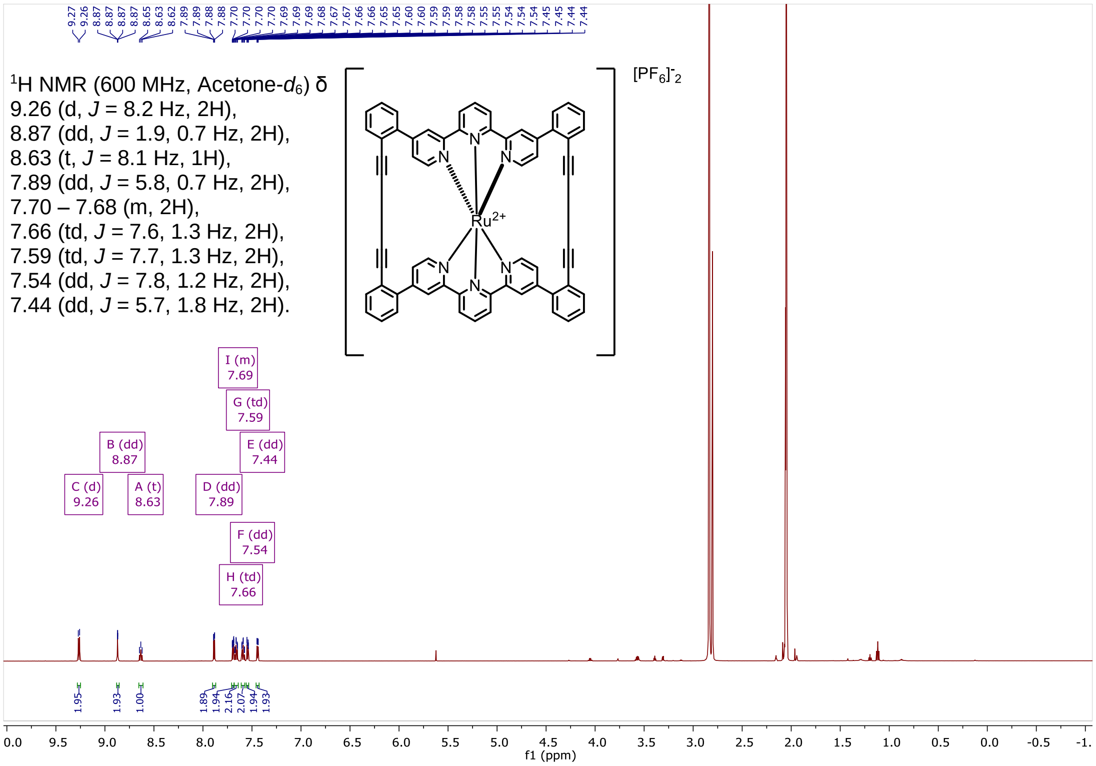

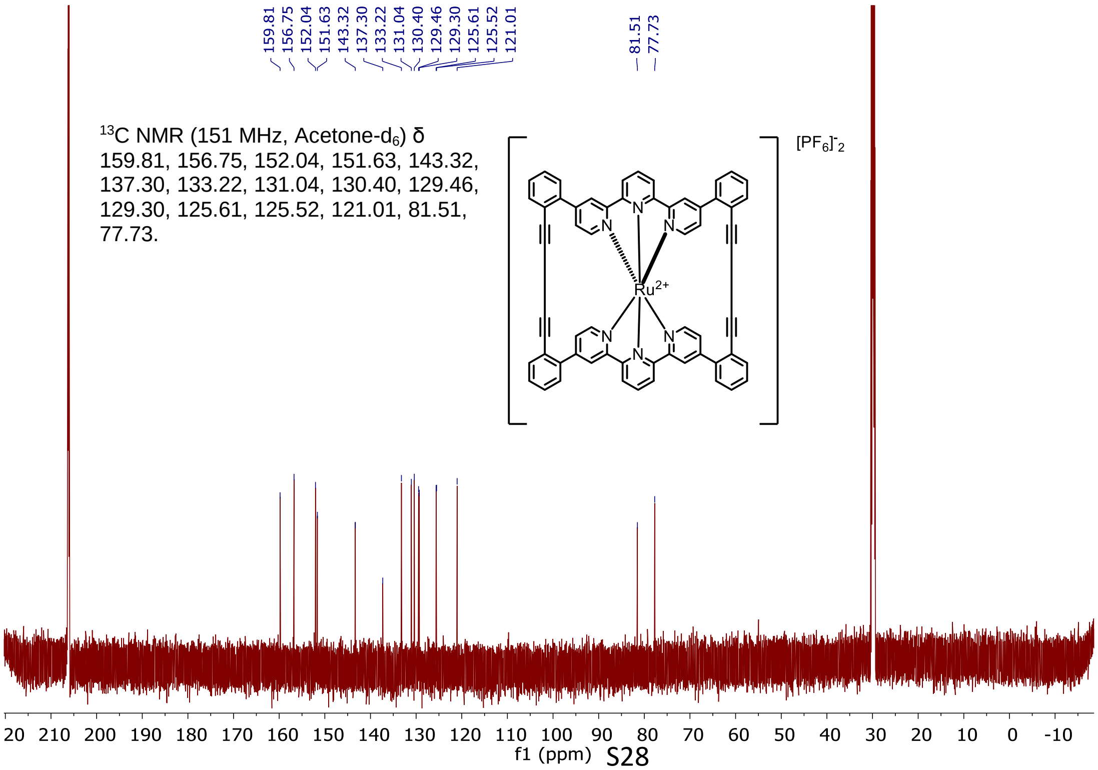

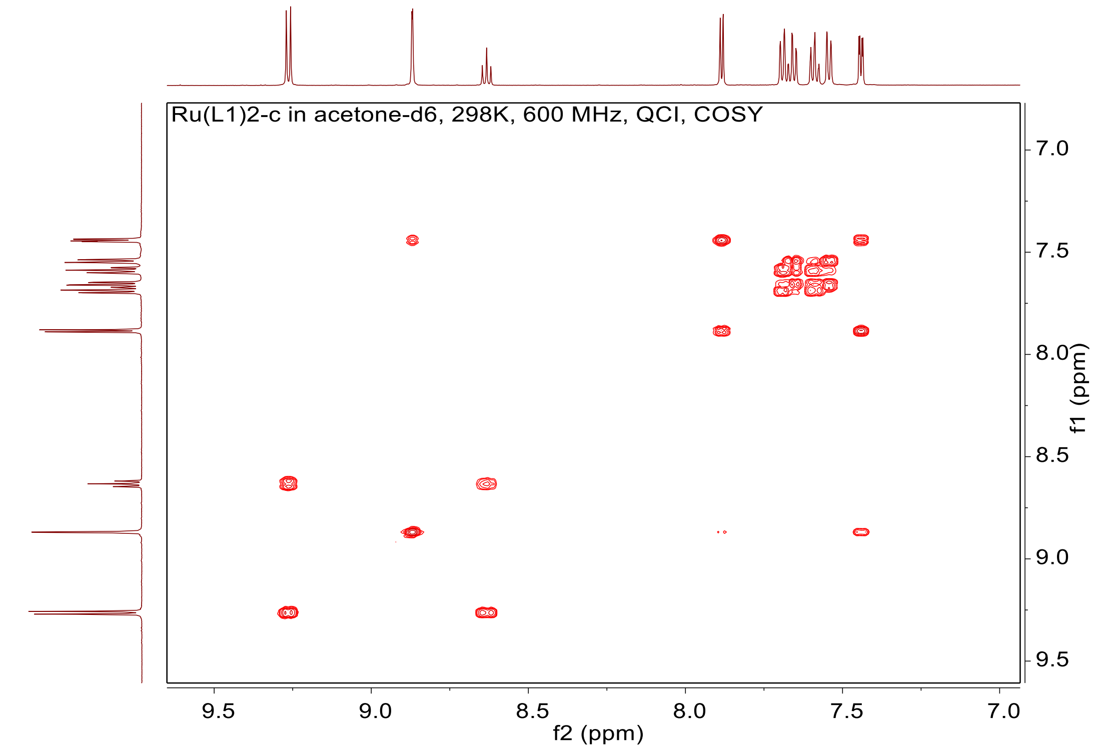
Ru(L1)2-c in acetone-d6, 298K, 600 MHz, QCI, COSY
f1 (ppm)
7.0
7.5
8.0
8.5
9.0
9.5
9.5
9.0
8.5
8.0
7.5
7.0
f2 (ppm)

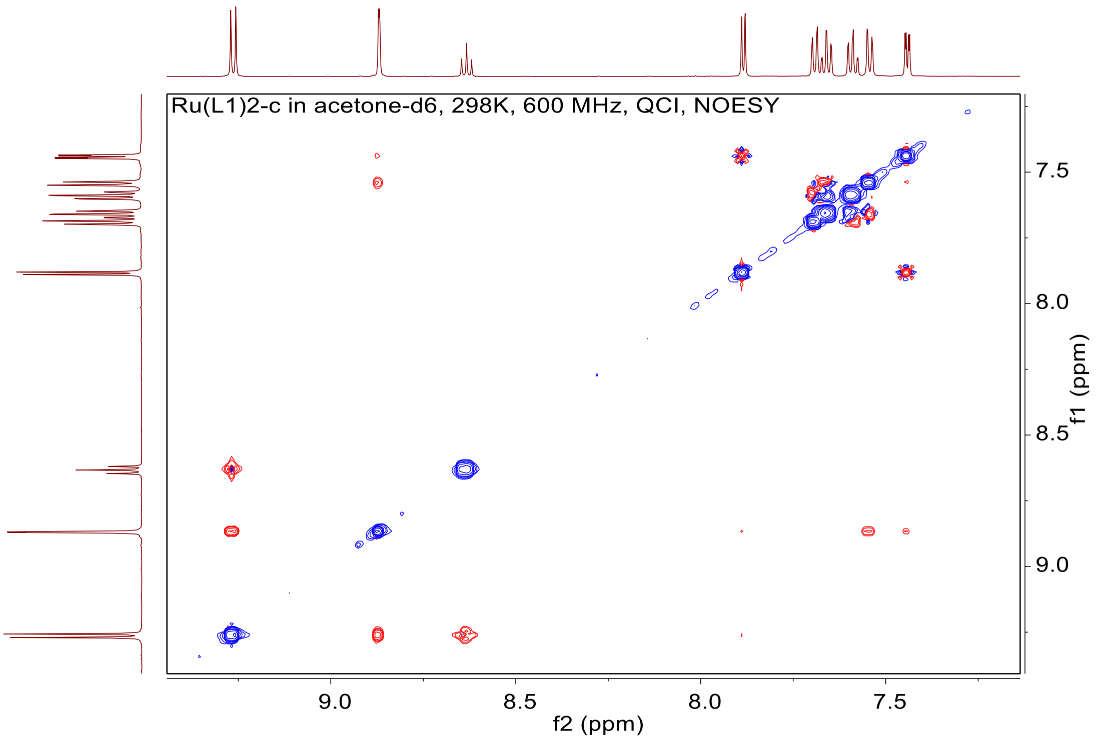
Ru(L1)2-c in acetone-d6, 298K, 600 MHz, QCI, NOESY
f1 (ppm)
7.5
8.0
8.5
9.0
9.0
8.5
8.0
7.5
f2 (ppm)

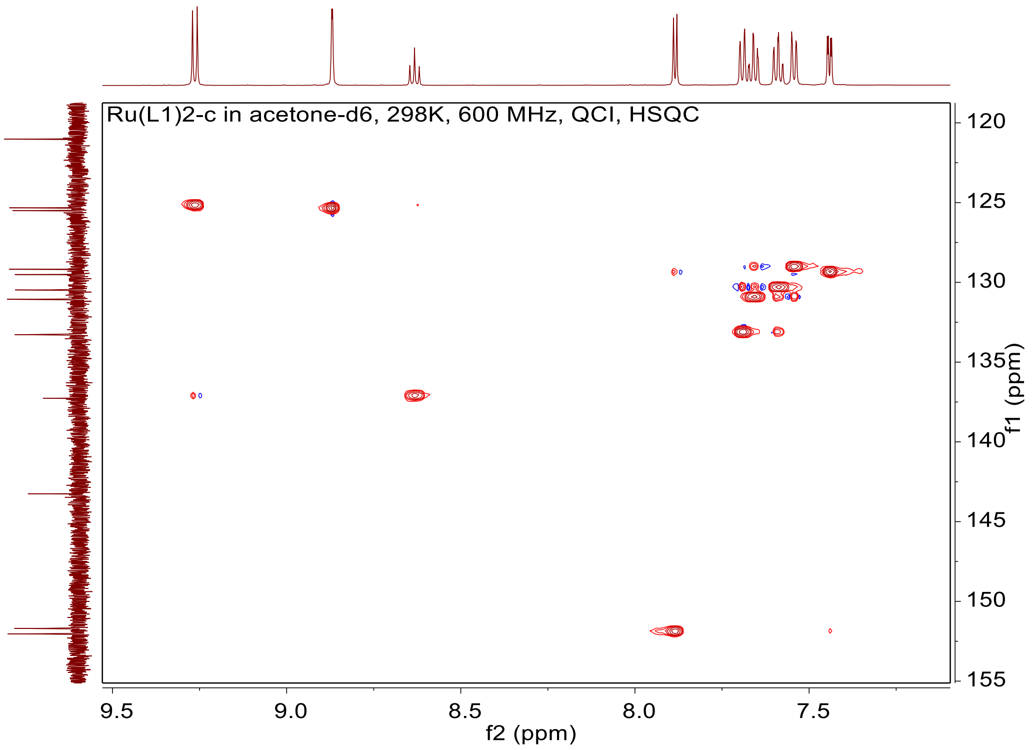
Ru(L1)2-c in acetone-d6, 298K, 600 MHz, QCI, HSQC
f1 (ppm)
f2 (ppm)

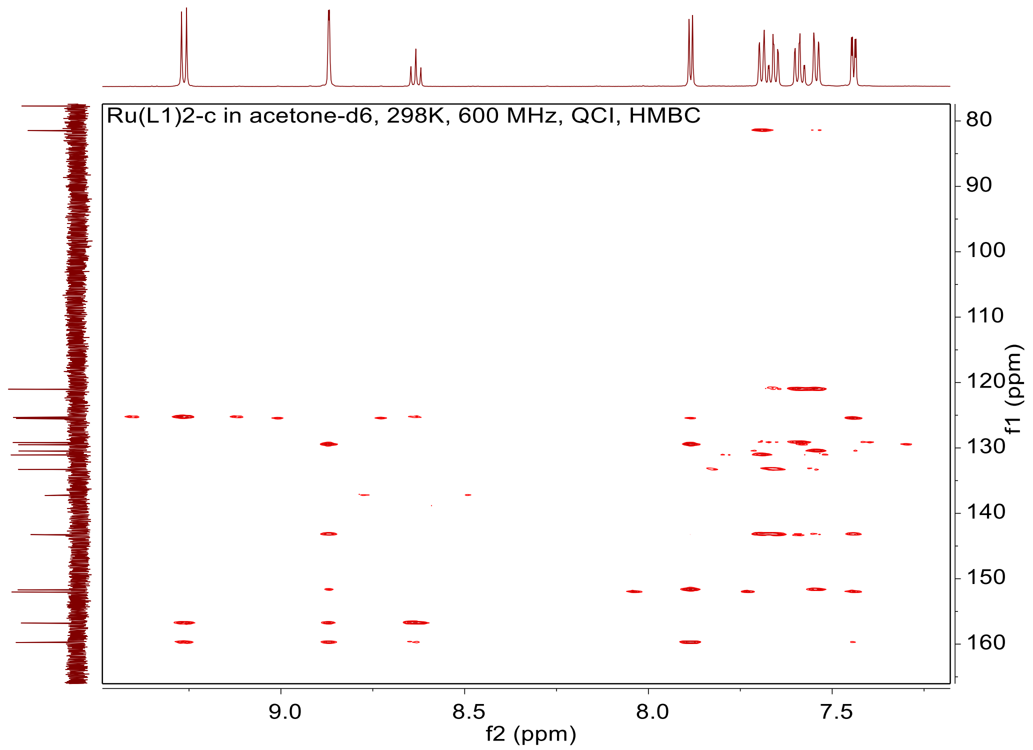
Ru(L1)2-c in acetone-d6, 298K, 600 MHz, QCI, HMBC
f1 (ppm)
f2 (ppm)

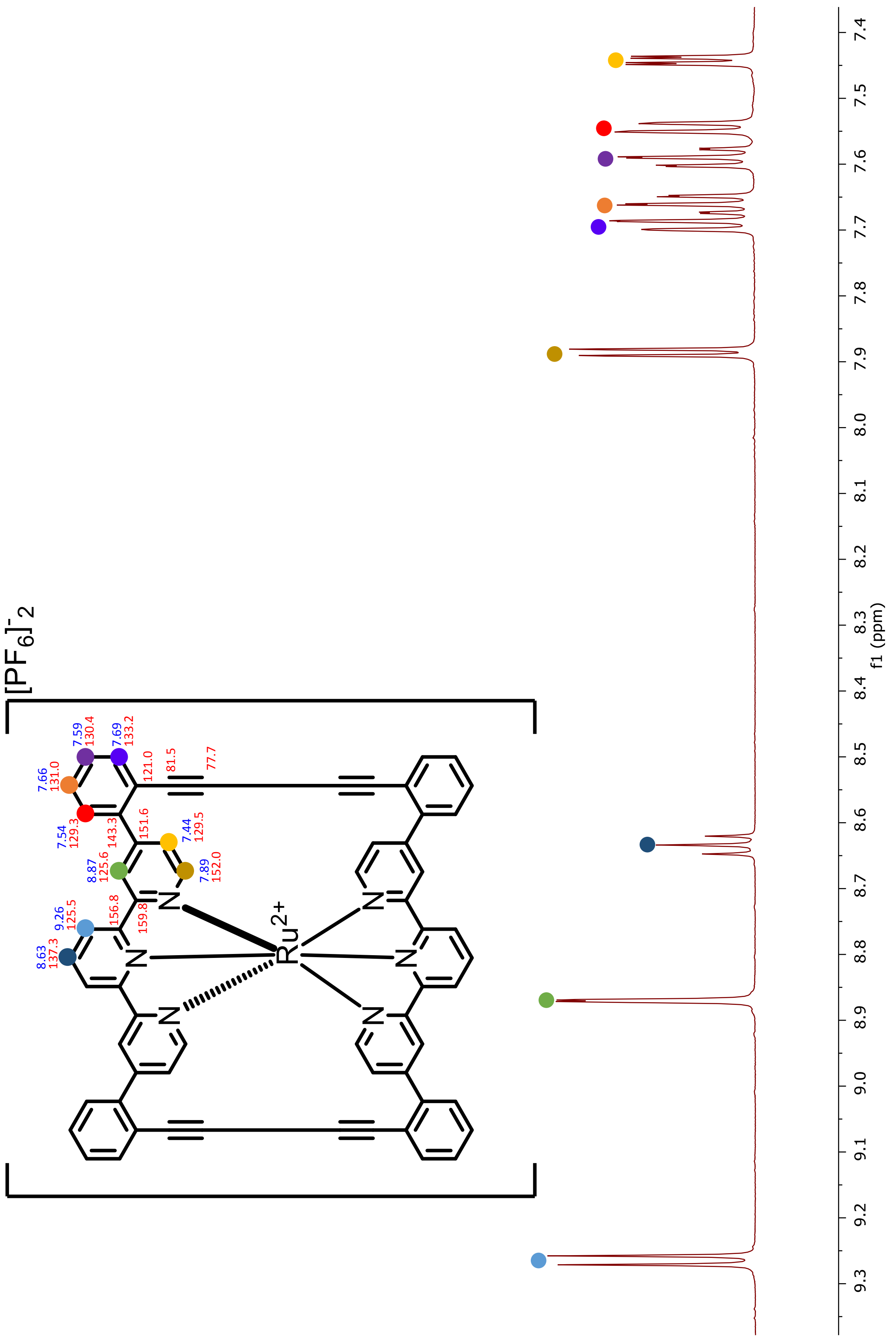

[PF6]-2
Ru2+
f1 (ppm)

Mass Spectrum SmartFormula Report

Analysis Info

Analysis Name	N:\new acq data\BRT129 002.d	Acquisition Date	30.11.2015 15:03:29
Method	hn Direct_Infusion_pos mode_sensitive Komplex.m	Operator	hn
Sample Name	Thomas Brandl, BRT129	Instrument / Ser#	maXis 4G 21243
Comment	ca. 20 ug/mL MeCN + DCM		

Acquisition Parameter

Source Type	ESI	Ion Polarity	Positive	Set Nebulizer	0.4 Bar
Focus	Not active	Set Capillary	4500 V	Set Dry Heater	180 °C
Scan Begin	75 m/z	Set End Plate Offset	-500 V	Set Dry Gas	4.0 l/min
Scan End	1600 m/z	Set Collision Cell RF	300.0 Vpp	Set Ion Energy (MS only)	30.0 eV

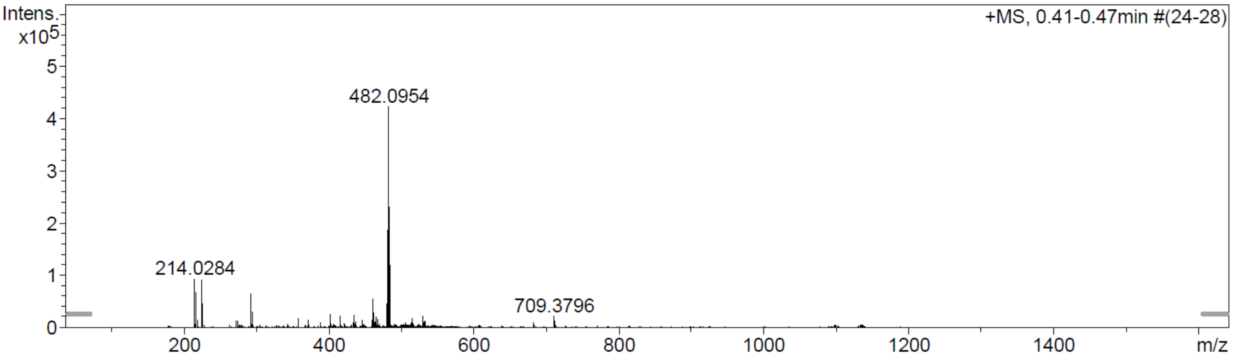

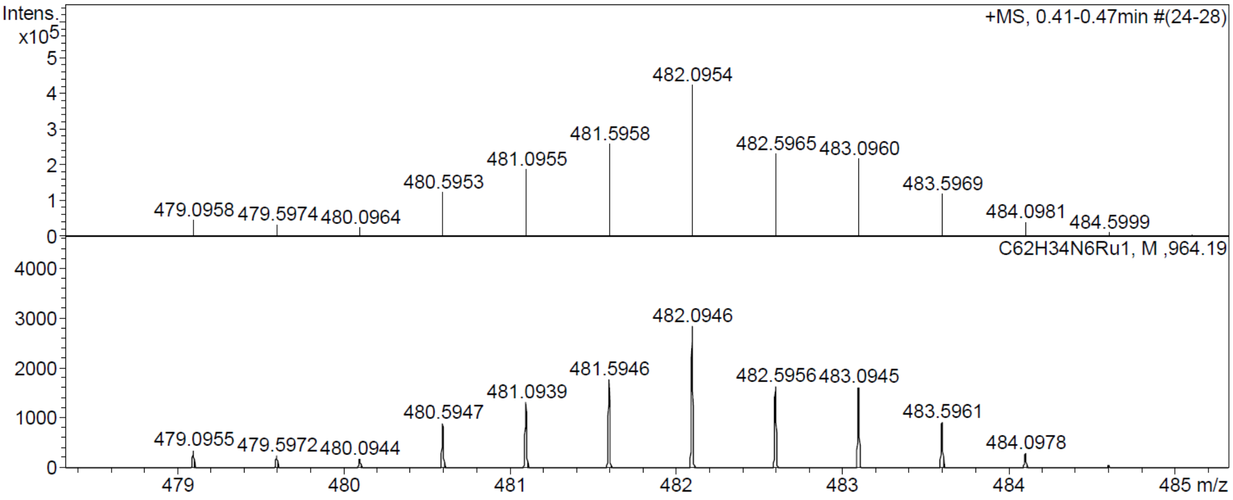

Meas. m/z	#	Formula	Score	m/z	err [mDa]	err [ppm]	mSigma	rdb	e⁻ Conf	N-Rule	z
482.0954	1	C 62 H 34 N 6 Ru	100.00	482.0947	-0.7	-1.5	23.6	49.5	odd	-	2+

Decay of emission signal of the complexes Ru(L1)$_2$-c and Ru(L1)$_2$ at 77 K:

Luminescence lifetimes and transient absorption spectra were recorded on an LP920-KS spectrometer from Edinburgh Instruments equipped with an iCCD detector from Andor. The excitation source was the frequency-doubled output from a Quantel Brilliant b laser.Decay of the emission signal at 630 nm of the open and the closed Ru(II) complexes in butyronitrile at 77K. The initial intense signal is due to laser stray light. Excitation occurred at 532 nm with laser pulses of ca. 10 ns duration.

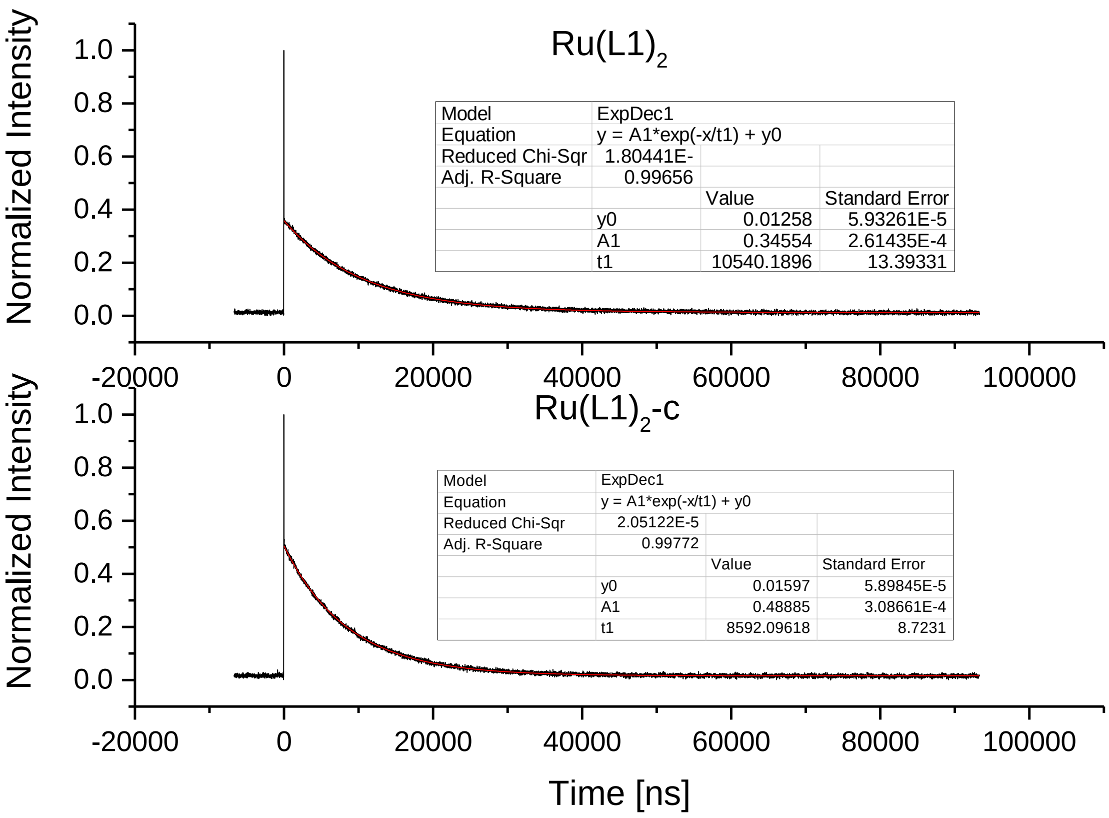

Figure SI 1: Decay of the emission signal at 630 nm of the open and the closed Ru(II) complexes in butyronitrile at 77K. The initial intense signal is due to laser stray light. Excitation occurred at 532 nm with laser pulses of ca. 10 ns duration.

UV-Vis spectroscopy:

The UV-Vis spectra were recorded on a *Shimadzu* UV spectrometer UV-1800. λ_{max} was measured in nm. All solutions were prepared and measured under air saturated conditions in acetonitrile. UV/Vis spectra were recorded on a Shimadzu UV spectrometer UV-1800 using optical 1115F-QS Hellma cuvettes (10 mm light path). The wavelength of maxima absorption maxima (λmax) are reported in nm.

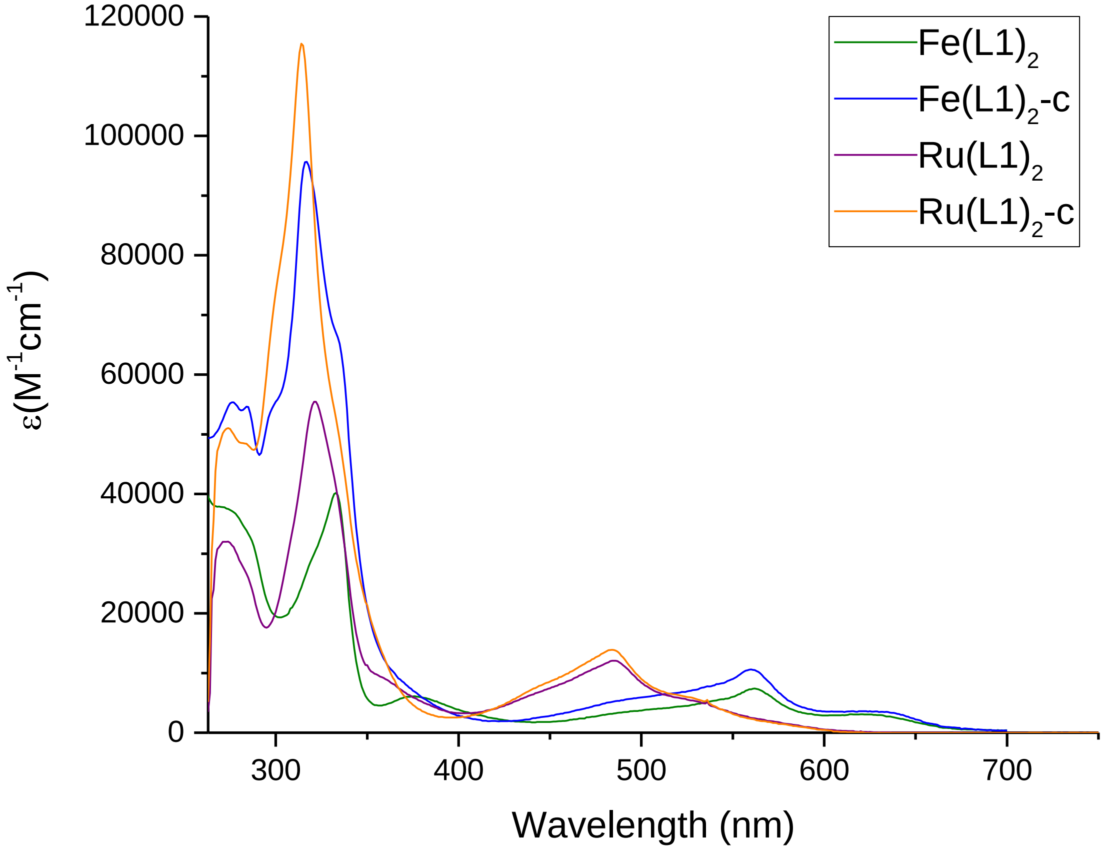

Figure SI 2: UV-Vis spectra of the open Fe(II) complex **Fe(L1)$_2$**, the closed Fe(II) complex **Fe(L1)$_2$-c**, the open Ru(II) complex **Ru(L1)$_2$** and the closed Ru(II) complex **Ru(L1)$_2$-c**.

Cyclic Voltammetry measurements:

Cyclic voltammetry was performed on a Versastat3- 200 potentiostat from Princeton Applied Research using a glassy carbon disk as working electrode, a silver wire as pseudo reference electrode, and a silver wire as counter electrode, 0.1 M solution of $TBAPF_6$ in dichloromethane served as supporting electrolyte. Small amounts of ferrocene were added for internal voltage calibration. Prior to voltage sweeps at rates of 0.1 V s^{-1}, the solutions were flushed with argon. Half-wave potentials ($E_{1/2}$) were calculated from the average of reductive and oxidative peak potential. Peak separations (ΔE) are reported in Table SI 1.

Table SI 1: Half wave potential $E_{1/2}$ in V vs. ferrocene and peak separations of the oxidative and the reductive potential ΔE.

Complex	Oxidation		Reduction 1		Reduction 2	
	$E_{1/2}$ [V]	ΔE [V]	$E_{1/2}$ [V]	ΔE [V]	$E_{1/2}$ [V]	ΔE [V]
**$Fe(L1)_2$**	0.72	0.121	-1.58	0.123	-1.79	0.130
**$Fe(L1)_2$-c**	0.82	0.082	-1.53	0.078	-1.76	0.097
**$Ru(L1)_2$**	0.89	0.087	-1.59	0.112	-1.88	0.152
**$Ru(L1)_2$-c**	0.99	0.095	-1.54	0.079	-1.87	0.131

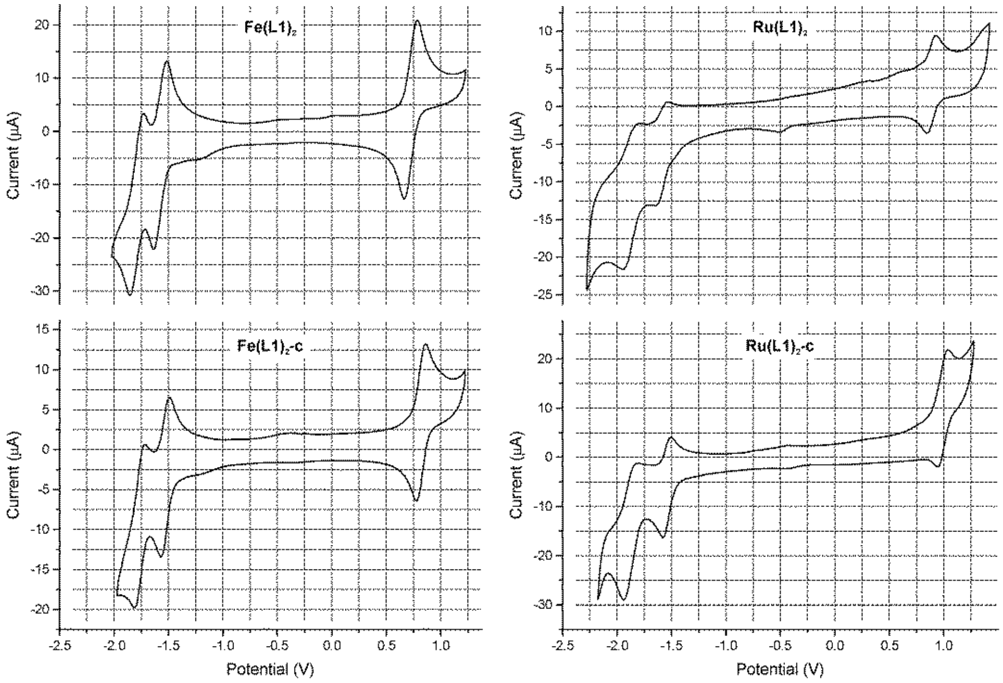

Figure SI 3. Cyclic voltammograms of the open and macrocyclized complexes (recorded in 0.1 m $TBAPF_6$ in DCM).

Computational Methodology:

All density functional theory (DFT) calculations were carried out by using the GAUSSIAN 09 suite. Geometries for all complexes were optimized computationally using Becke's three- parameter exchange functional (B3)[2] in conjunction with the Lee, Yang, and Parr (LYP)[3] nonlocal functional. No geometry restrictions were used during the optimization and the LANL2DZ basis set was employed for all molecules.[4] Additional calculations on the Fe complexes were performed with the 6-31G(d) basis set.[5]

[2] Becke, A. D*. J. Chem. Phys.* **1993**, 98, 5648.
[3] (a) Lee, C.; Yang, W.; Parr, R. G. *Phys. Rev. B* **1988**, 37, 785. (b) Miehlich, B.; Savin, A.; Stoll, H.; Preuss, H. *Chem. Phys. Lett.* **1989**, 157, 200.
[4] (a) Hay, P. J.; Wadt, W. R. *J Chem. Phys.* **1985**, 82, 299. (b) Hay, P. J.; Wadt, W. R. *J Chem. Phys.* **1985**, 82, 270. (c) Hay, P. J.; Wadt, W. R. *J. Chem. Phys.* **1985**, 82, 284.
[5] Hariharan, P. C.; Pople, J. A. *Theor. Chim. Acta.* **1973**, 28, 213.

Racemization experiments and determination of the inversion barrier of $Fe(L1)_2$-c:

The complexes were irradiated with a blue LED at 450 nm (3 Watt) for 12 hours. Therefore one enantiomer of the complexes was dissolved in acetonitrile and placed in a thin test tube. The glass was placed in a shaker to move the solution and the LED was placed in close proximity (2 cm). Even after irradiating for 12 hours no racemization of the enantiomer was observed. For the next racemization experiment a “Polychrome V monochromator” from Till Photonics monochromator equipped with a 150 W Xenon high stability lamp and an optical fiber was used. The output power is specified as >10 mW at 470 nm. Irradiation in solution at 560 nm with a spectral transmission band of 2 nm of one enantiomer in acetonitrile for 12 hours did not result in racemization as monitored by HPLC.

Racemization experiments by oxidation and successive reduction were performed on a Versastat3- 200 potentiostat from Princeton Applied Research using a glassy carbon disk as working electrode, a silver wire as pseudo reference electrode, and a silver wire as counter electrode, 0.1 M solution of $TBAPF_6$ in dichloromethane served as supporting electrolyte. Even applying an oxidation potential of 1.5 V for 30 minutes and successive reductive potential of 0 V for 30 minutes did not lead to racemization.

The racemization experiments were performed in a high boiling solvent. Therefore several solvents like ethylene glycol, *o*-xylene, DMSO, 1-hexanol and *p*-cymene were tested. Unfortunately none of these solvents dissolved the $Fe(L1)_2$-c complex. Suitable solvents that dissolves the complex like EtOH, acetonitrile, acetone, dichloromethane or 1,2-dichloroethane have a too low boiling point. The only possible solvents found were 1,1,2,2-tetrachlorethane and DMF. Due to possible side-reaction that sometimes arise in DMF close to refluxing temperatures, 1,1,2,2-tetrachlorethane was used for the racemization experiment. In order to be consistent in the experiment, always the first obtained enantiomer from the chiral HPLC was used.

The rate of racemization between the two enantiomers of $Fe(L1)_2$-c can be described as a first order process with

$$ln\left(\frac{[A]_t}{[A]_0}\right) = -k_{rac}t \qquad \textbf{(1)}$$

with $[A]_0$ being the enantiomeric excess at t = 0, $[A]_t$ the enantiomeric excess at the observed time t and k_{rac} the rate constant for the racemization.

The decay of the enantiomeric excess was determined by HPLC. Each temperature was measured three times and each sample was injected three times to HPLC in order to take the average enantiomeric excess values:

Table SI 2: Racemization measurement at 60 °C

**60 °C 1st measurement**						
time [s]	1st Injection	2nd Injection	3rd Injection	Average	ee	$ln\left(\frac{[A]_t}{[A]_0}\right)$
0	75.3	75.4	76.2	75.63333	51.26667	0
9000	73.7	73.4	73.9	73.66667	47.33333	-0.07983
14400	73.3	72.4	72.2	72.63333	45.26667	-0.12447
19800	72.5	71.1	71.8	71.8	43.6	-0.16198
25200	71.1	71.3	70.8	71.06667	42.13333	-0.1962
32400	70.1	70.4	70.8	70.43333	40.86667	-0.22673
39600	69.5	68.9	68.7	69.03333	38.06667	-0.2977
63900	65.1	64.8	65.7	65.2	30.4	-0.5226
76500	63.7	64.2	64.1	64	28	-0.60484
86400	62.8	62.2	63.4	62.8	25.6	-0.69445

**60 °C 2nd measurement**						
time [s]	1st Injection	2nd Injection	3rd Injection	Average	ee	$ln\left(\frac{[A]_t}{[A]_0}\right)$
0	77	76.4	75.9	76.43333	52.86667	0
9000	74.4	74.8	74.2	74.46667	48.93333	-0.07731
14400	74.6	74.1	74.2	74.3	48.6	-0.08415
19800	72.6	73.6	73	73.06667	46.13333	-0.13624
25200	72	72	69.2	71.06667	42.13333	-0.22693
32400	71.5	71.1	71.9	71.5	43	-0.20657
39600	69.5	70	69.9	69.8	39.6	-0.28894
63900	65.3	65.7	66.3	65.76667	31.53333	-0.51673
76500	64	63.5	64	63.83333	27.66667	-0.64754
86400	63.5	62.5	62.5	62.83333	25.66667	-0.72258

**60 °C 3rd measurement**						
time [s]	1st Injection	2nd Injection	3rd Injection	Average	ee	$ln\left(\frac{[A]_t}{[A]_0}\right)$
0	76.5	76.3	76.3	76.36667	52.73333	0
9000	75.5	75.3	75.1	75.3	50.6	-0.0413
14400	74.5	74.2	74.7	74.46667	48.93333	-0.07479
19800	72.7	73.6	73.1	73.13333	46.26667	-0.13083
25200	72.1	71.6	73.7	72.46667	44.93333	-0.16007
32400	69.5	70.4	71.5	70.46667	40.93333	-0.2533

39600	69.9	69	70.1	69.66667	39.33333	-0.29318
63900	65.9	65.4	65.8	65.7	31.4	-0.51844
76500	64.4	64.1	63.8	64.1	28.2	-0.62593
86400	62.6	61.9	62.7	62.4	24.8	-0.7544

Table SI 3: Racemization measurement at 70 °C

**70 °C 1st measurement**						
time [s]	1st Injection	2nd Injection	3rd Injection	Average	ee	$ln\left(\frac{[A]_t}{[A]_0}\right)$
0	98.9	98.6	98.6	98.7	97.4	0
750	97.5	97.8	97.6	97.63333	95.26667	-0.02215
3450	94.5	94.4	94.3	94.4	88.8	-0.09244
11550	86	86	86.1	86.03333	72.06667	-0.30123
21450	78	77.8	77.8	77.86667	55.73333	-0.55825
26850	74.6	74.6	75	74.73333	49.46667	-0.67753
30450	72.8	72.6	72.4	72.6	45.2	-0.76773
34050	70.6	70.8	70.7	70.7	41.4	-0.85555
43050	67.4	67.4	67.6	67.46667	34.93333	-1.02538
48450	65.3	66.3	65.9	65.83333	31.66667	-1.12356

**70 °C 2nd measurement**						
time [s]	1st Injection	2nd Injection	3rd Injection	Average	ee	$ln\left(\frac{[A]_t}{[A]_0}\right)$
0	98.7	98.9	98.8	98.8	97.6	0
900	97.9	97.8	97.6	97.76667	95.53333	-0.0214
3600	95	94.8	94.9	94.9	89.8	-0.08329
11700	87.1	86.7	87.3	87.03333	74.06667	-0.27591
21600	78.9	79.4	79.1	79.13333	58.26667	-0.51585
27000	75.6	75.4	75.6	75.53333	51.06667	-0.64775
30600	73.2	73.6	72.9	73.23333	46.46667	-0.74214
34200	71.5	71.4	71.1	71.33333	42.66667	-0.82746
43200	67.4	67.2	67.7	67.43333	34.86667	-1.02935
48600	65.4	65.6	65.5	65.5	31	-1.14689

**70 °C 3rd measurement**						
time [s]	1st Injection	2nd Injection	3rd Injection	Average	ee	$ln\left(\frac{[A]_t}{[A]_0}\right)$
0	98.8	98.9	99	98.9	97.8	0
900	97.4	97.3	97.6	97.43333	94.86667	-0.03045
3600	94.3	94.5	94.6	94.46667	88.93333	-0.09504

11700	86.4	86.2	86.1	86.23333	72.46667	-0.2998
21600	78.2	78.2	78.5	78.3	56.6	-0.54692
27000	74.7	75.1	74.5	74.76667	49.53333	-0.68028
30600	72.9	72.5	72.9	72.76667	45.53333	-0.76448
34200	70.8	70.8	70.8	70.8	41.6	-0.85482
43200	68.1	67.9	67.9	67.96667	35.93333	-1.00126
48600	65.5	65.6	65.5	65.53333	31.06667	-1.14679

Table SI 4: Racemization measurement at 80 °C

**80 °C 1st measurement**						
time [s]	1st Injection	2nd Injection	3rd Injection	Average	ee	$ln\left(\frac{[A]_t}{[A]_0}\right)$
0	94.3	94.6	94.4	94.43333	88.86667	0
360	92.5	92.3	92	92.26667	84.53333	-0.04999
720	90.3	90.6	90.3	90.4	80.8	-0.09516
1080	88.9	88.9	89.3	89.03333	78.06667	-0.12957
1440	87.4	87.1	87.1	87.2	74.4	-0.17768
1800	86.1	85.4	86.2	85.9	71.8	-0.21325
2160	84.6	84.5	84.7	84.6	69.2	-0.25014
2520	82.9	83.4	83.4	83.23333	66.46667	-0.29044
2880	82.3	82.5	82	82.26667	64.53333	-0.31996
3240	81	80.6	80.9	80.83333	61.66667	-0.36539
3600	79.8	80.3	79.8	79.96667	59.93333	-0.3939
3960	78.8	78.9	79.3	79	58	-0.42669
4320	77.4	77.5	77.4	77.43333	54.86667	-0.48223
4680	76.8	76.7	76.7	76.73333	53.46667	-0.50808
5040	75.6	75.9	76.2	75.9	51.8	-0.53975
5400	75.3	75.5	75.4	75.4	50.8	-0.55924
5760	73.9	73.7	74.1	73.9	47.8	-0.62011
6120	73.5	73.7	73.2	73.46667	46.93333	-0.63841
6480	71.9	72.6	72.8	72.43333	44.86667	-0.68344

**80 °C 2nd measurement**						
time [s]	1st Injection	2nd Injection	3rd Injection	Average	ee	$ln\left(\frac{[A]_t}{[A]_0}\right)$
0	93.8	93.8	94.2	93.93333	87.86667	0
360	91.4	92	91.9	91.76667	83.53333	-0.05057
720	90.3	90.3	90	90.2	80.4	-0.08881
1080	88.9	88.6	88.9	88.8	77.6	-0.12425
1440	87.2	87.1	87.1	87.13333	74.26667	-0.16816
1800	85.6	85.3	85.9	85.6	71.2	-0.21033

2160	84.5	84.8	84.7	84.66667	69.33333	-0.23689
2520	83.1	83.2	84	83.43333	66.86667	-0.27312
2880	81.7	82.1	81.2	81.66667	63.33333	-0.32741
3240	80.3	80.5	80.3	80.36667	60.73333	-0.36933
3600	79.4	79.1	79.2	79.23333	58.46667	-0.40736
3960	78.2	78.4	79	78.53333	57.06667	-0.4316
4320	77	77	77	77	54	-0.48684
4680	75.7	76.5	76.3	76.16667	52.33333	-0.51819
5040	74.6	75.1	75.2	74.96667	49.93333	-0.56513
5400	74.6	74.6	74.7	74.63333	49.26667	-0.57857
5760	73.6	73.1	73.4	73.36667	46.73333	-0.63136
6120	72.2	72.5	72.7	72.46667	44.93333	-0.67064
6480	71.6	71.6	71.8	71.66667	43.33333	-0.7069

**80 °C 3rd measurement**						
time [s]	1st Injection	2nd Injection	3rd Injection	Average	ee	$ln\left(\frac{[A]_t}{[A]_0}\right)$
0	93.7	94	93.8	93.83333	87.66667	0
360	91.9	92.1	92.1	92.03333	84.06667	-0.04193
720	90.2	90.7	90.7	90.53333	81.06667	-0.07827
1080	88.8	88.7	89	88.83333	77.66667	-0.12112
1440	87	87.5	87.3	87.26667	74.53333	-0.1623
1800	86	85.9	85.5	85.8	71.6	-0.20245
2160	84.6	85.1	84.4	84.7	69.4	-0.23365
2520	83.5	83.6	84.1	83.73333	67.46667	-0.26191
2880	82.5	82	82.3	82.26667	64.53333	-0.30636
3240	81.1	81.2	81	81.1	62.2	-0.34319
3600	79.6	79.9	79.9	79.8	59.6	-0.38589
3960	78.3	78.3	78.8	78.46667	56.93333	-0.43166
4320	77.4	77.7	77.4	77.5	55	-0.46621
4680	77.2	75.9	76.4	76.5	53	-0.50325
5040	75.9	75.3	75.8	75.66667	51.33333	-0.5352
5400	75.6	75.1	74.7	75.13333	50.26667	-0.5562
5760	74.1	74	73.9	74	48	-0.60234
6120	72.9	72.8	72.6	72.76667	45.53333	-0.6551
6480	72.1	72	72	72.03333	44.06667	-0.68784

Table SI 5: Racemization measurement at 90 °C

**90 °C 1st measurement**						
time [s]	1st Injection	2nd Injection	3rd Injection	Average	ee	$ln\left(\frac{[A]_t}{[A]_0}\right)$

0	86.5	86.6	86.1	86.4	72.8	0
360	82.1	82.2	82.8	82.36667	64.73333	-0.11744
720	79.5	79.3	78.6	79.13333	58.26667	-0.22269
1080	76.1	75.9	76.3	76.1	52.2	-0.33263
1440	73.4	74	73.2	73.53333	47.06667	-0.43615
1800	72.2	71.5	71.5	71.73333	43.46667	-0.51572
2160	69.7	69.2	69.9	69.6	39.2	-0.61904
2520	67.7	68.1	67.6	67.8	35.6	-0.71537
2880	66.1	66.5	65.9	66.16667	32.33333	-0.81162
3240	64.9	64.2	65.1	64.73333	29.46667	-0.90446
3600	63.7	63	63.3	63.33333	26.66667	-1.0043

**90 °C 2nd measurement**						
time [s]	1st Injection	2nd Injection	3rd Injection	Average	ee	$ln\left(\frac{[A]_t}{[A]_0}\right)$
0	85.7	85.8	86	85.83333	71.66667	0
360	81.9	82.2	81.4	81.83333	63.66667	-0.1203
720	78.5	78.2	78.7	78.46667	56.93333	-0.23177
1080	76.3	76.3	75.7	76.1	52.2	-0.32074
1440	72.9	73.7	73.5	73.36667	46.73333	-0.42217
1800	71.3	71.5	71.5	71.43333	42.86667	-0.5131
2160	68.8	70.1	69.5	69.46667	38.93333	-0.60006
2520	67.7	67.3	67.3	67.43333	34.86667	-0.72685
2880	66.5	66.3	65.6	66.13333	32.26667	-0.80684
3240	64.4	64.5	64.9	64.6	29.2	-0.89454
3600	63.2	62.8	63.6	63.2	26.4	-0.9999

**90 °C 3rd measurement**						
time [s]	1st Injection	2nd Injection	3rd Injection	Average	ee	$ln\left(\frac{[A]_t}{[A]_0}\right)$
0	86.6	86	86.6	86.4	72.66667	0
360	82	82.4	82.4	82.26667	64.71111	-0.11595
720	79.2	79.2	79	79.13333	58.22222	-0.22162
1080	76.4	76.5	76.4	76.43333	52.88889	-0.31769
1440	73.9	74	73.8	73.9	47.8	-0.41886
1800	71.8	71.8	71.5	71.7	43.33333	-0.51696
2160	69.4	69.8	69.7	69.63333	39.42222	-0.61155
2520	68	68	67.5	67.83333	35.55556	-0.71479
2880	66.1	66.7	66.3	66.36667	32.91111	-0.79207
3240	64.8	65.3	64.4	64.83333	29.68889	-0.89511
3600	63.5	63.6	64	63.7	27.53333	-0.97049

Plotting t against $ln\left(\frac{[A]_t}{[A]_0}\right)$ gives a linear relation with the slope that corresponds to k_{rac} directly:

Table SI 6: Rate constants for the different temperatures

Temperature		1st measurement	2nd measurement	3rd measurement	Average	Standard deviation
60 °C	k=	7.99E-06	8.5E-06	8.83E-06	8.4395E-06	4.25891E-07
70 °C	k=	2.37E-05	2.39E-05	2.36E-05	2.3732E-05	1.08805E-07
80 °C	k=	0.000103	0.000108	0.000105	0.00010545	2.6183E-06
90 °C	k=	0.000275	0.000273	0.000269	0.00027242	2.79383E-06

The k values can be used to determine the free Gibbs energy of racemization $\Delta G^{\neq}$ by the rearranged Eyring equation:

$$\Delta G^{\neq}_{(T)} = RT\left[ln\left(\frac{\kappa k_B T}{h}\right) - \ln\left(\frac{k_{rac}}{2}\right)\right] \qquad \textbf{(2)}$$

Where k_{rac} is the obtained kinetic rate constant, κ the transition factor ($\kappa = 0.5$), k_B the Boltzmann constant (k_B = 1.380662 x 10^{-23} J K^{-1}), h the Planck's constant (h = 6.626176 x 10^{-34} J s), R the universal gas constant (R = 8.31446 J K^{-1} mol^{-1}) and T the temperature in Kelvin.

Table SI 7: Calculated values for the free Gibbs energy in kJ/mol

$\Delta G^{\neq}_{(K)}$[kJ/mol]	1st measurement	2nd measurement	3rd measurement	Average	Standard deviation
$\Delta G^{\neq}_{(353.15K)}$	121.5	121.3	121.2	121.3	0.15
$\Delta G^{\neq}_{(363.15K)}$	118.3	118.2	118.3	118.3	0.01
$\Delta G^{\neq}_{(373.15K)}$	113.9	113.8	113.9	113.9	0.07
$\Delta G^{\neq}_{(383.15K)}$	111.1	111.1	111.1	111.1	0.03

Using the Eyring equation

$$k = \frac{\kappa k_B T}{h} e^{-\frac{\Delta G^{\neq}}{RT}} \qquad \textbf{(3)}$$

And substituting $\Delta G^{\neq}$ by

$$\Delta G^{\neq}_{(T)} = \Delta H^{\neq} - T\Delta S^{\neq} \qquad \textbf{(4)}$$

gives

$$k = \frac{\kappa k_B T}{h} e^{\frac{-\Delta H^{\neq} + T\Delta S^{\neq}}{RT}} \qquad \textbf{(5)}$$

which can be brought in a linear form:

$$ln\left(\frac{k}{T}\right) = -\left(\frac{\Delta H^{\neq}}{R}\right)\left(\frac{1}{T}\right) + \frac{\Delta S^{\neq}}{R} + ln\frac{\kappa k_B}{h} \quad \textbf{(6)}$$

This equation states that plotting $ln\left(\frac{k}{T}\right)$ vs. $\frac{1}{T}$ yield a straight line with slope = $-\left(\frac{\Delta H^{\neq}}{R}\right)$ and intersect $= \frac{\Delta S^{\neq}}{R} + ln\frac{\kappa k_B}{h}$.

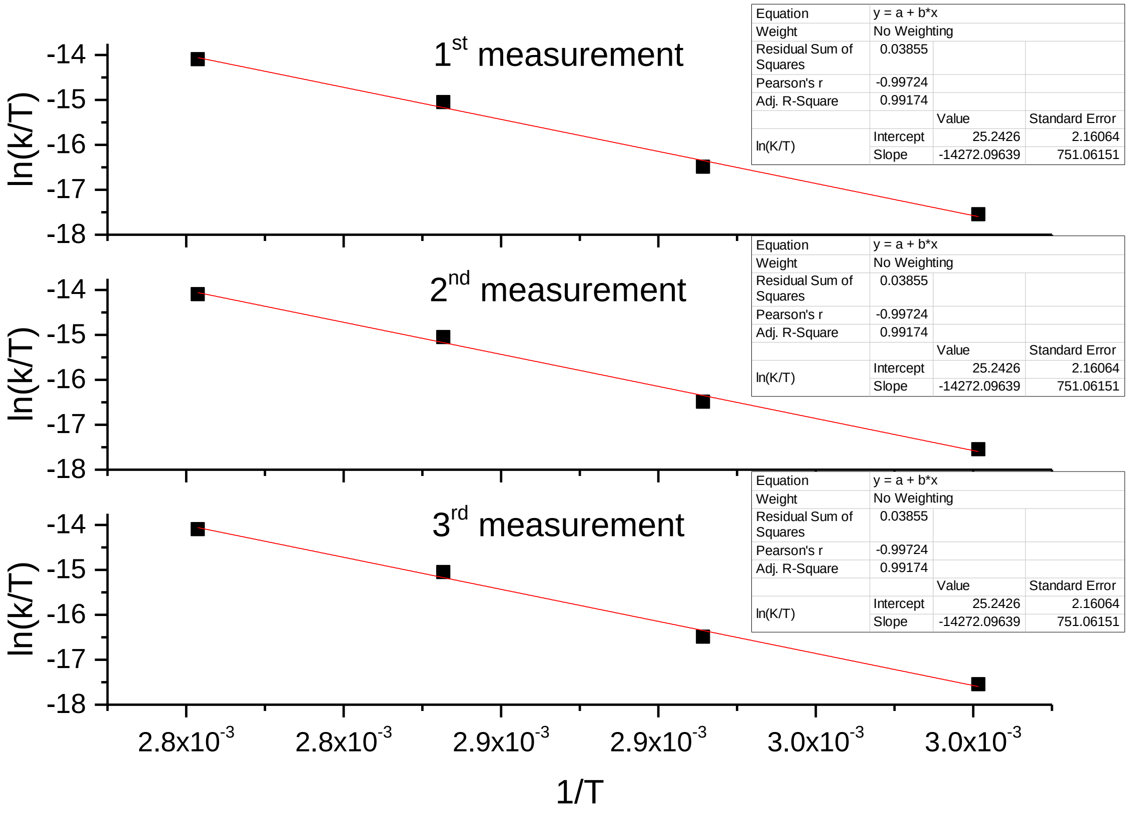

Figure SI 4: Eyring plot

Table SI 8: The obtained values for $\Delta H^{\neq}$and $\Delta S^{\neq}$ are:

	1st measurement	2nd measurement	3rd measurement	Average	Standard deviation
$\Delta H^{\neq}$ [kJ/mol]	118.7	117.0	115.2	117.0	1.73
$\Delta S^{\neq}$ [J/(mol*K)]	18.1	13.6	8.3	13.4	4.9

With the Arrhenius equation $k = Ae^{-\frac{E_a}{RT}}$ it is possible to plot $ln(k)$ vs. $\frac{1}{T}$ to get the activation energy:

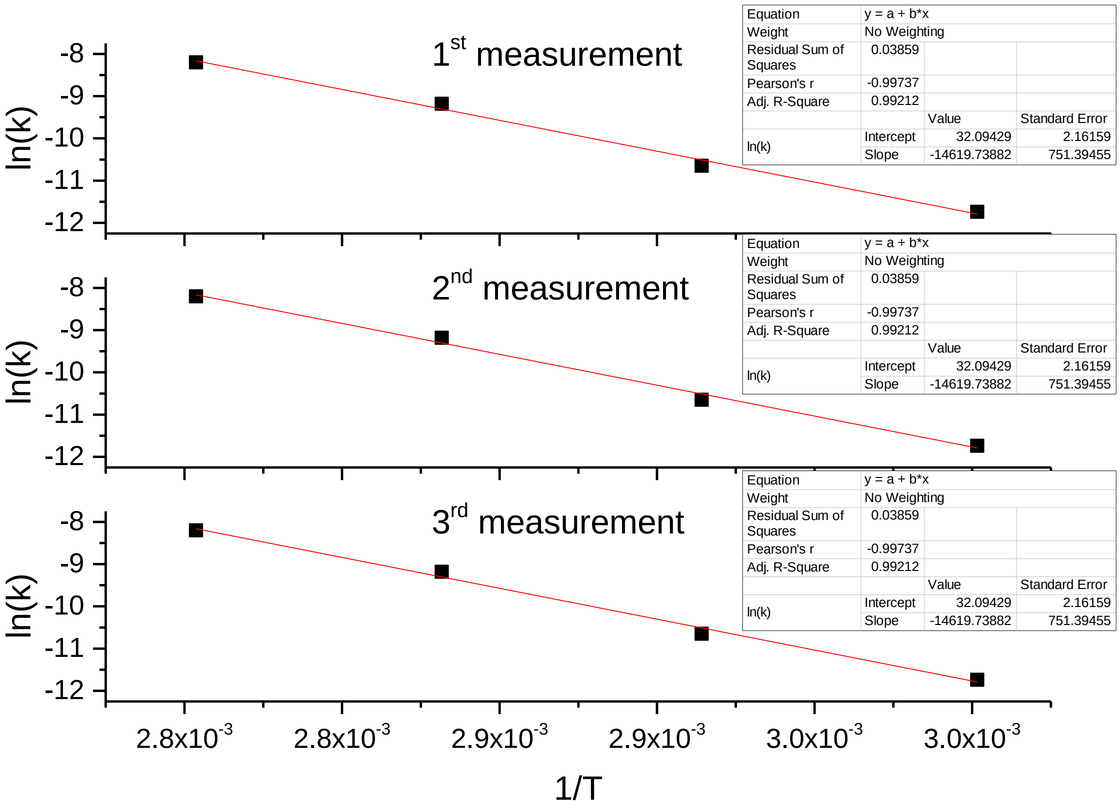

Figure SI 5: Arrhenius plot

Table SI 9: The obtained values for E_a are:

	1st measurement	2nd measurement	3rd measurement	Average	Standard deviation
E_a [kJ/mol]	121.6	119.9	118.1	119.9	1.73

Furthermore, by using the Arrhenius plot, it is possible to recalculate $\Delta G^{\neq}_{(298.15K)}$ and $k_{298.15K}$ in order to get the $t_{1/2}$ at these temperatures:

Table SI 10: Recalculated values at room temperature:

	1st measurement	2nd measurement	3rd measurement	Average	Standard deviation
$\Delta G^{\neq}_{(298.15K)}$ [kJ/mol]	115.0	114.7	114.5	114.7	0.28
$k_{298.15K}$	4.38838E-08	4.97E-08	5.49E-08	4.95E-08	5.52E-09
$t_{1/2}$ in days	182.8	161.5	146.1	163.5	18.4

Crystal data for Ru(L1)$_2$-c and Fe(L1)$_2$-c:

Compound **Ru(L1)$_2$-c** crystallizes in the tetragonal space group $P\overline{4}2_1c$ with four formula units and four additional molecules of diethyl ether per unit cell. The Ru atom of the dication $[Ru(N_6C_{62}H_{34})]^{2+}$ (figure SI 5) is located on a crystallographic 2-fold axis; and it is coordinated by the six nitrogen atoms of the macrocyclic ligand, which are forming a highly distorted octahedron (*cis*-N-Ru-N angles 78.7°–103.1°; trans-N-Ru-N angles: 157.3° and 177.3°, figure SI 7).

Compound **Fe(L1)$_2$-c** crystallizes in the orthorhombic space group Fdd2 with eight formula units per unit cell. Also in this structure the metal atom is located on a twofold axes of the space group Fdd2. Figure SI 9 illustrates the similarity of the two compounds by overlaying them on top of each other. The bond angles around the Fe atom differ for this reason only slightly (*cis*-N-Fe-N angles 80.5°–99.7°; trans-N-Fe-N angles: 161.3° and 179.7°).

For both structures the diffraction data were collected on a Stoe StadiVari diffractometer attached to a Ga Metaljet X-ray source at low temperature.

Compound **Ru(L1)$_2$-c**: Using Olex2[6], the structure was solved with the ShelXS[7] structure solution program using Direct Methods and refined with the ShelXL[8] refinement package using Least Squares minimization. Non-hydrogen atoms were refined anisotropically (atoms of the disordered solvent molecule were refined isotropically); hydrogen atoms were modeled on idealized positions. Molecular drawings were generated using Diamond3.2[9].

Compound **Fe(L1)$_2$-c**: The structure was solved with the program Superflip[10] using the charge flipping method and refined with CRYSTALS[11] using Least Squares minimization. Non-hydrogen atoms were refined anisotropically. About 37% of the volume of the structure is occupied by disordered solvent molecules. As interpretation of the electron density map in terms of molecules was not possible SQUEEZE[12] has been used in order to complete the refinement. The electron

[6] O.V. Dolomanov, L.J. Bourhis, R.J. Gildea, J.A.K. Howard, H. Puschmann, *J. Appl. Cryst.* **2009**, *42*, 339–341.
[7] G.M. Sheldrick, *Acta Cryst.* **2008**, *A64*, 112–122.
[8] G.M. Sheldrick, *Acta Cryst.* **2015**, *C71*, 3–8.
[9] K. Brandenburg, Crystal Impact GbR, Bonn, Germany, 1997-2016.
[10] L. Palatinus, G. Chapuis, *J. Appl. Cryst.* **2007**, 40, 786-790.
[11] P.W. Betteridge, J.R. Carruthers, R.I. Cooper, K. Prout, D.J. Watkin, *J. Appl. Cryst.* **2003**, 36, 1487.
[12] A.L.Spek, *Acta Cryst.* **2009**, D65, 148-155.

density removed by SQUEEZE corresponds to about 230 electrons per formula unit. Hydrogen atoms were included in the refinement as fixed contributions. Molecular drawings were generated using Mercury[13].

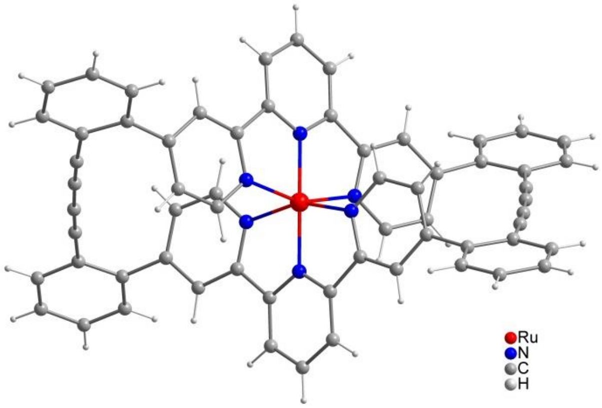

Figure SI 6: Molecular structure of the dication $[Ru(N_6C_{62}H_{34})]^{2+}$ in **1**.

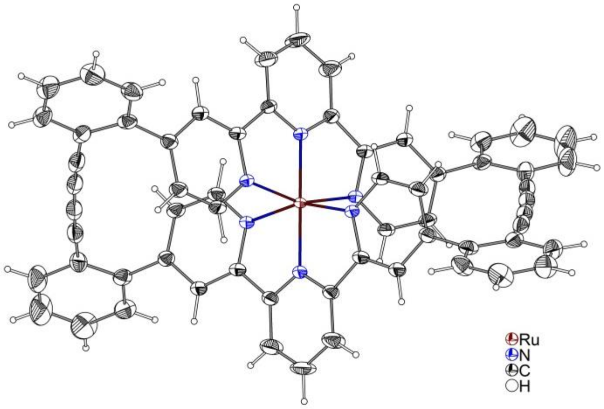

Figure SI 7: Ortep Plot (50 % probability) of the dication $[Ru(N_6C_{62}H_{34})]^{2+}$ in **1**.

[13] C. F. Macrae, I. J. Bruno, J. A. Chisholm, P. R. Edgington, P. McCabe, E. Pidcock, L. Rodriguez-Monge, R. Taylor, J. van de Streek and P. A. Wood, *J. Appl. Cryst.* **2008**, 41, 466-470.

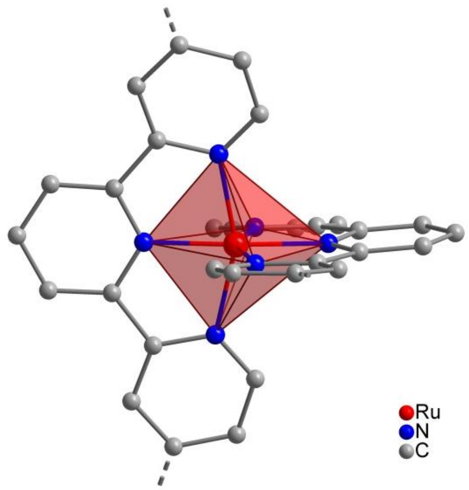

Figure SI 8: The coordination sphere around the Ru atom in the dication $[Ru(N_6C_{62}H_{34})]^{2+}$ in **1**.

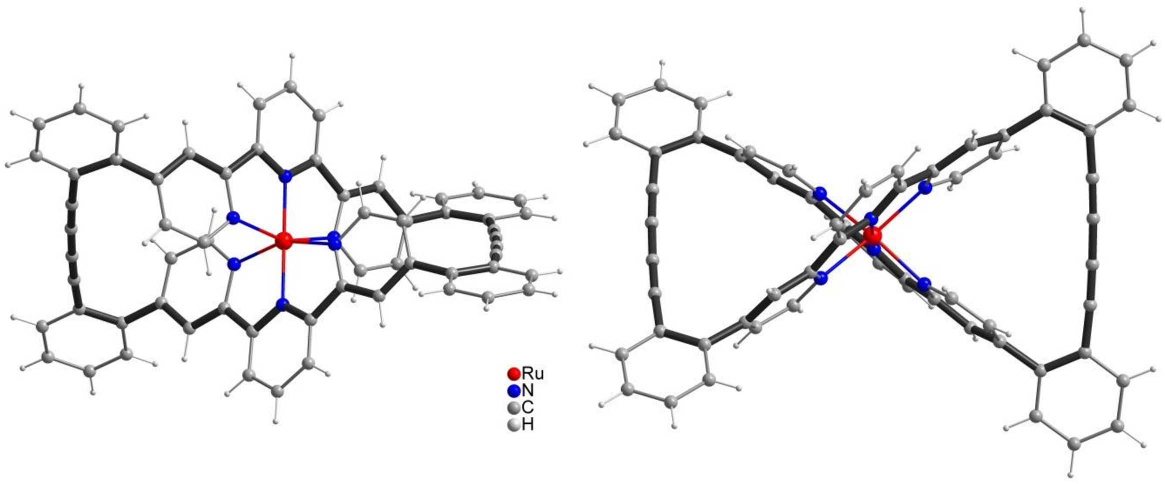

Figure SI 9: Two representations of the dication $[Ru(N_6C_{62}H_{34})]^{2+}$ in **1** (rotated by 90°) demonstrating the conformation of the macro-cyclic ligand around the Ru atom.

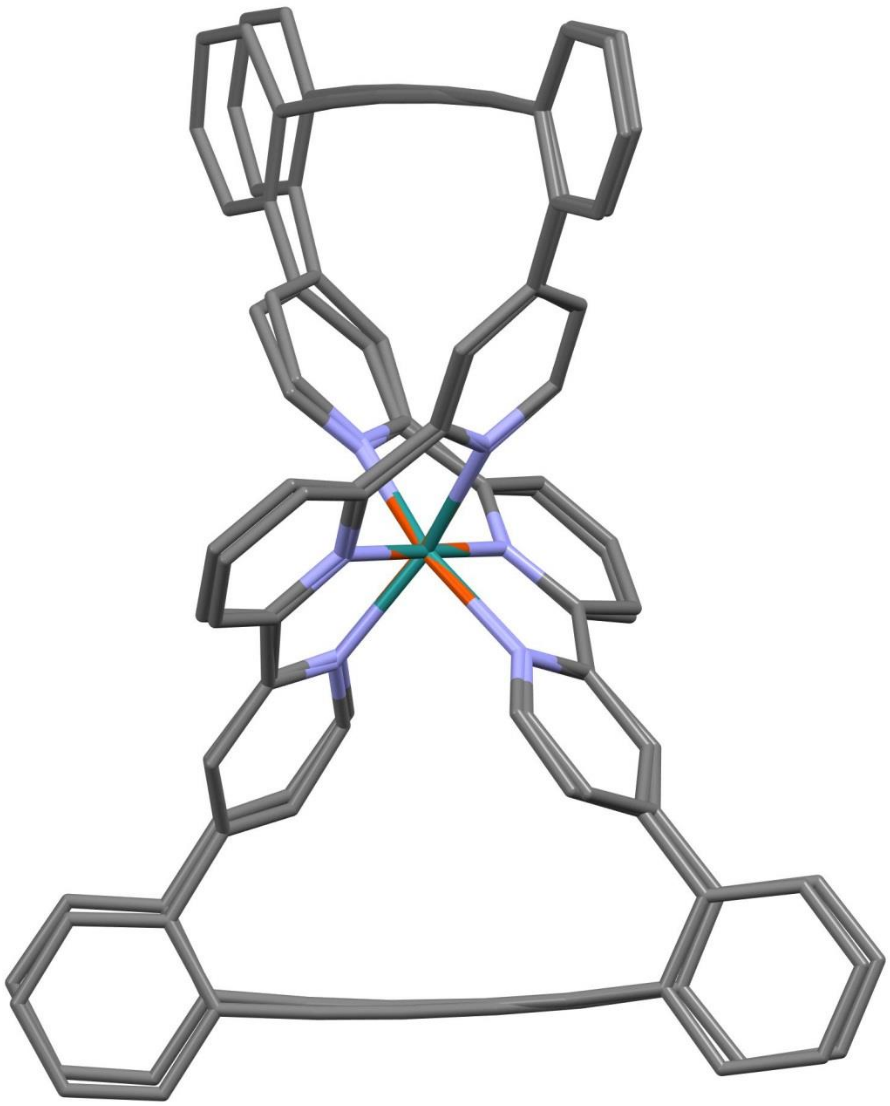

Figure SI 10: Overlay of the two compounds, the iron atom is displayed in orange, the ruthenium atom in green.

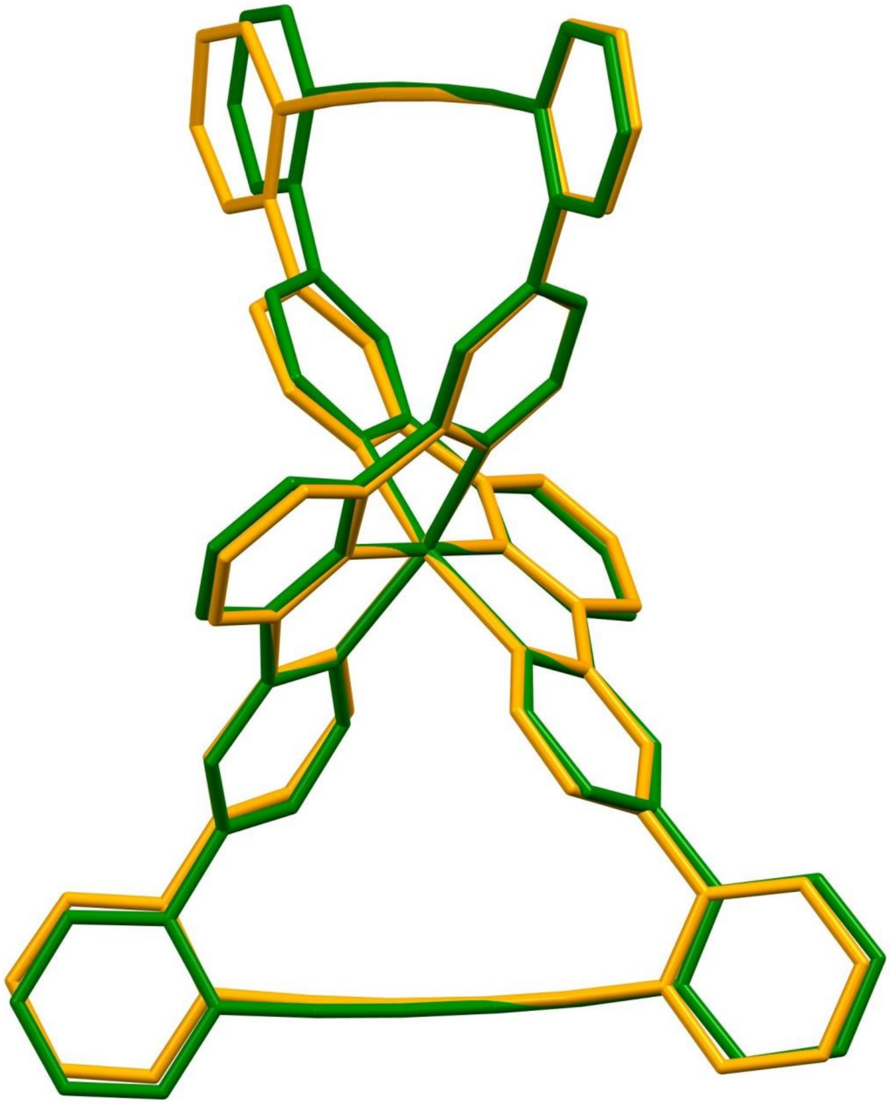

Figure SI 11: Overlay of the two compounds, the iron complex is displayed in yellow, the ruthenium complex in green.

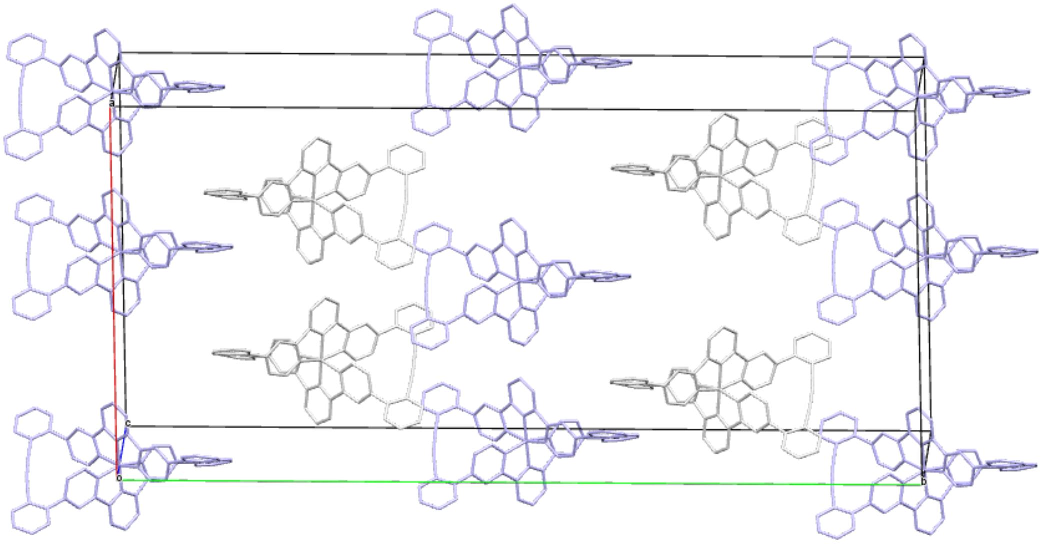

Figure SI 12: X-Ray structure of racemic **Fe(L1)$_2$-c** The cell contains 8 molecules, four of each enantiomer. The hydrogen atoms and the PF_6^- counter ion were omitted for clarity reasons. Colour code: bridge: green, carbon: grey, nitrogen: blue, iron: orange (a,b) ; enantiomers: light blue, light grey (c).

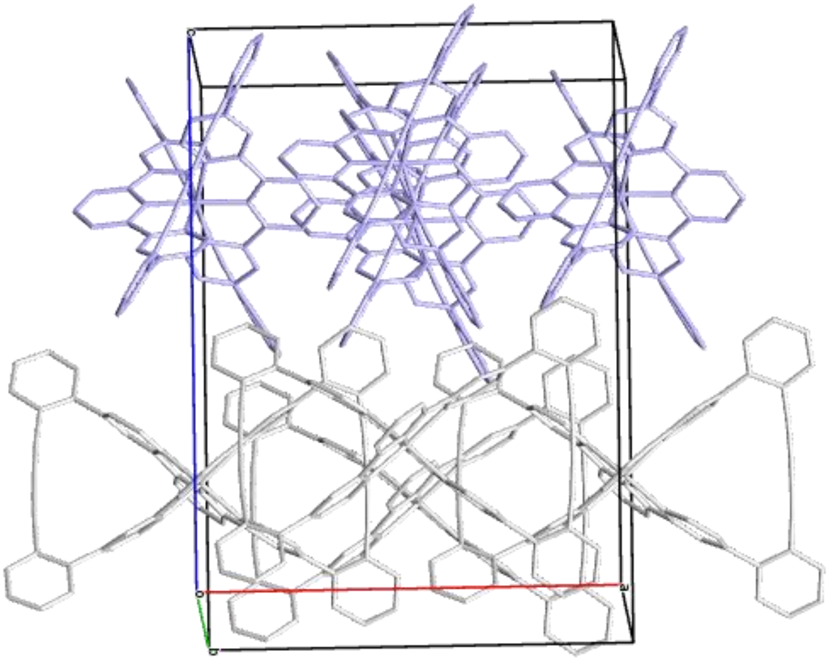

Figure SI 13: X-Ray structure of racemic **Ru(L1)$_2$-c**. The cell contains 4 molecules, two of each enantiomer. The hydrogen atoms and the PF_6^- counter ions were omitted for clarity reasons. Colour code: enantiomers: light blue, light grey.

Table SI 11: Crystal data and structure refinement for Ru(L1)$_2$-c and Fe(L1)$_2$-c

Compound	**Ru(L1)$_2$-c** · $C_4H_{10}O$	**Fe(L1)$_2$-c**
Empirical formula	$C_{66}H_{44}F_{12}N_6OP_2Ru$<br>$[Ru(N_6C_{62}H_{34})](PF_6)_2 \cdot C_4H_{10}O$	$C_{62}H_{34}F_{12}FeN_6P_2$<br>$[Fe(N_6C_{62}H_{34})](PF_6)_2$
Formula weight	1328.08	1208.76
Temperature/K	123.0	123.0
Crystal system	tetragonal	orthorhombic
Space group	$P\overline{4}2_1c$	Fdd2
a/Å	16.39200(10)	27.5449(3)
b/Å	16.39200(10)	55.4159(8)
c/Å	21.9038(2)	10.2412(15)
α/°	90	90
β/°	90	90
γ/°	90	90
Volume/Å^3	5885.50(9)	15632(2)
Z	4	8
ρ_{calc}g/cm^3	1.499	1.027
μ/mm^{-1}	2.263	1.669
F(000)	2688.0	4896.0
Crystal size/mm^3	0.21 × 0.14 × 0.07	0.14 × 0.11 × 0.03
Radiation	GaKα (λ = 1.34143)	GaKα (λ = 1.34143)
2Θ range for data collection/°	5.858 to 114.876	5.55 to 120.062
Index ranges	–15 ≤ h ≤ 20<br>–20 ≤ k ≤ 18<br>–27 ≤ l ≤ 27	–33 ≤ h ≤ 35<br>–71 ≤ k ≤ 68<br>–7 ≤ l ≤ 13
Reflections collected	119994	60208
Independent reflections	6077 [R_{int} = 0.0278, R_σ = 0.0061]	6558 [R_{int} = 0.025]
Data/restraints/parameters	6077/6/397	5455/1/376
Goodness-of-fit on F^2	1.027	1.049
Final R indexes [I≥2σ(I)]	R_1 = 0.0335, wR_2 = 0.0967	R_1 = 0.0366, wR_2 = 0.0428
Final R indexes [all data]	R_1 = 0.0341, wR_2 = 0.0974	R_1 = 0.0399, wR_2 = 0.0455
Largest diff. peak/hole / e Å^{-3}	0.54/–0.79	0.23/–0.27
Flack parameter	–0.018(2)	0.488(5)
CCDC number	1533848	1533847

HPLC Chromatograms:

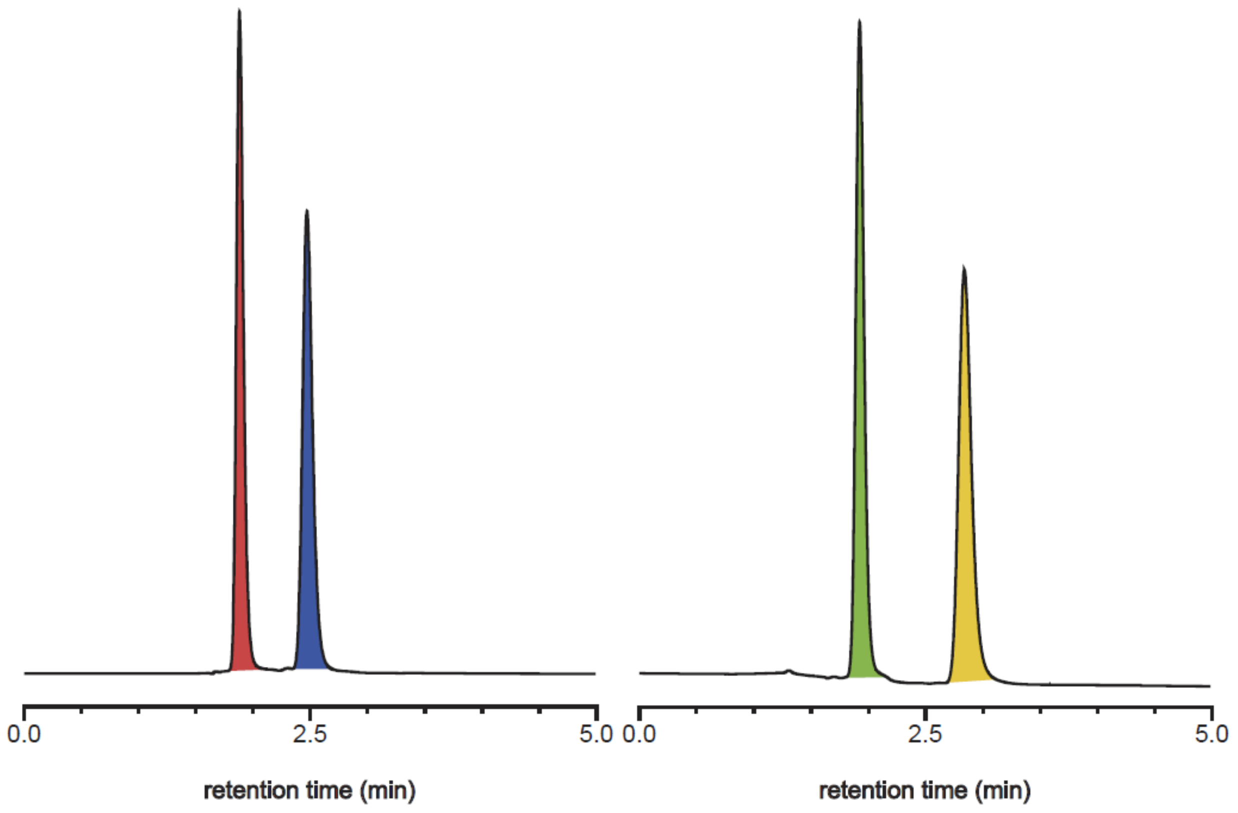

Figure SI 14: HPLC chromatograms of the racemates, showing the enantiomeric resolution for **Fe(L1)$_2$-c** (left) and **Ru(L1)$_2$-c** (right). Chiralpak IB column, eluent: EtOH/MeOH/TEA/TFA 50:50:0.5:0.3, 2 mL min^{-1}, T = 40 °C.

High dilution macrocyclization experiment of the free ligand L1:

An interesting aspect is the importance of the templating in a M(II) complex for the success of the macrocyclization. In order to shine light on this issue, high dilution macrocyclization experiments were performed with the ligand **L1**. Based on the experience we have in the lab with high dilution experiments, the active concentration of **L1** was further reduced by adding it with a syringe pump. In spite of several approaches, the closed macrocycle **(L1)$_2$** could neither be detected in reasonable yields nor be isolated. Particular challenging for the high dilution reaction is that the copper ions required for the oxidative coupling form complexes with the pyridiyl-subunits of **L1** as well. Furthermore is seems that metal free oligomers of **L1** have only limited solubility. However, in the most successful attempt traces of the dimers of **L1** have been observed by MALDI-ToF mass spectrometry. In this attempt CuCl (4.71 mg, 46.1 μmol, 10 eq.) and TMEDA (6.99 μL, 46.1 μmol, 10 eq.) were dissolved in DCM (50 mL). The reaction mixture was saturated with oxygen before the ligand **L1** (2.00 mg, 4.61 μmol, 1.0 eq) dissolved in DCM (20 mL) was added over a period of 4 hours. The procedure results in a maximum concentrations of the ligand **L1** of 6.6 $\cdot 10^{-8}$ mol/L. The reaction was monitored by MALDI-ToF mass spectrometry (see figure SI 15).

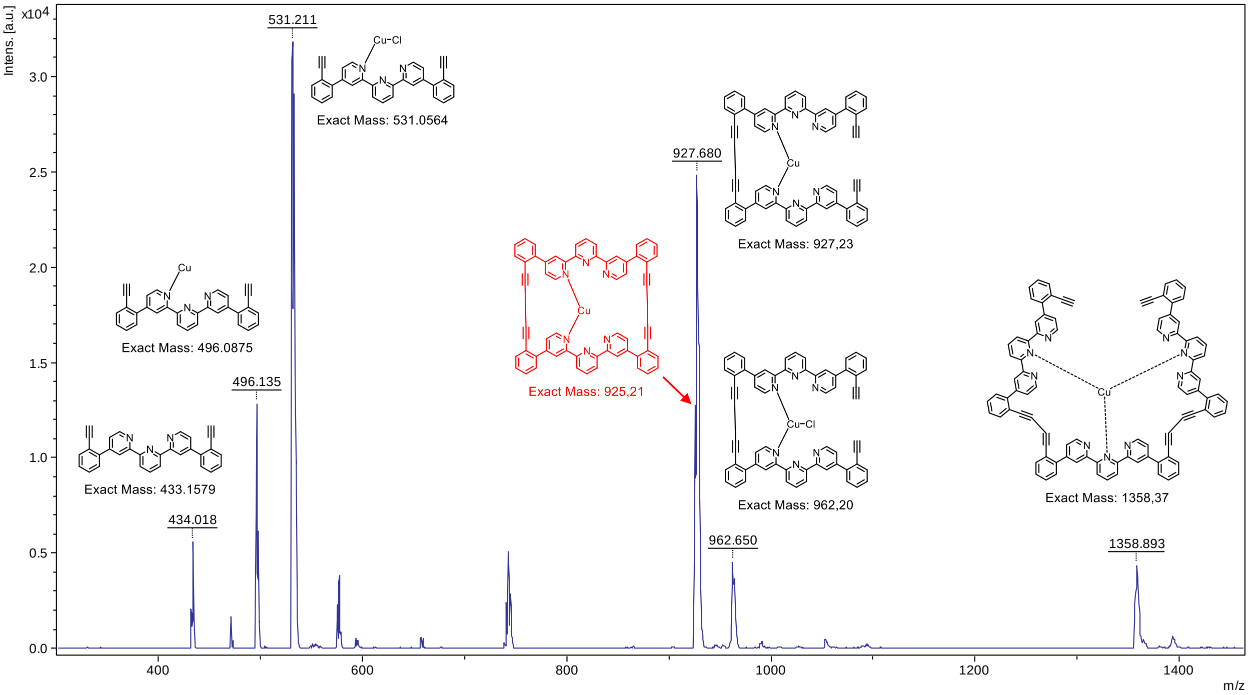

Figure SI 15: MALDI-ToF MS spectrum of the high dilution macrocyclization of ligand **L1**. The dimer and trimer seem to be a mixture between linked and open adducts.

The most prominent signals in the MS are the ones of the ligand **L1** and its copper adducts. But there are also signals that can be assigned to the copper adducts of the singly closed dimer of **L1** and even a weak signal that can be assigned to the copper complex of the doubly closed dimer of **L1**, which is the desired macrocycle **(L1)$_2$** (red arrow in Fig. SI 15). Interestingly, also the signal of the copper adduct of the next higher oligomer **L1$_3$** can be detected. However, all attempts to isolate the macrocycle **(L1)$_2$** failed. After work up and extraction of the copper ions, exclusively the monomer **L1** was detected by analyzing the reaction mixture by HPLC. Whether the traces of

the macrocycle were too little for the detection threshold of the HPLC-MS device or its detection was hampered by its solubility features we are not able to distinguish.

Closer inspection of the MALDI-ToF MS signal of the **Cu-L1$_2$** adducts (Fig. SI 16) suggest that the signal corresponds to about a 4:5 mixture of the copper adducts of the doubly closed macrocycle and the single closed open dimer.

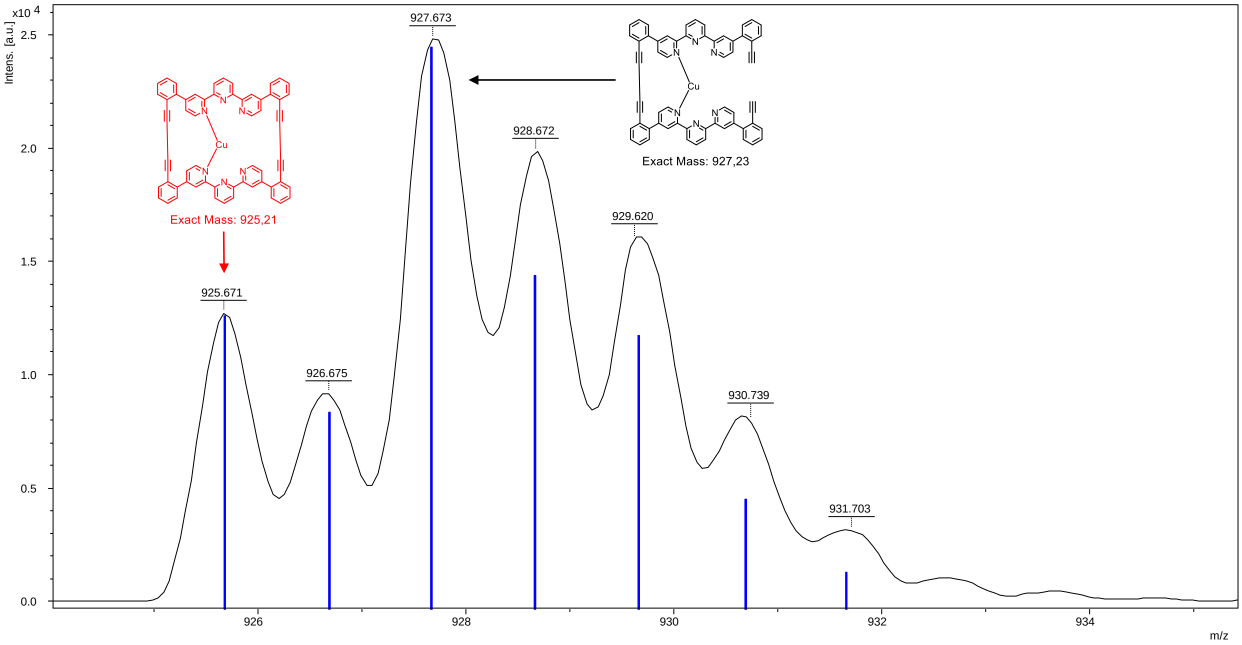

Figure SI 16: MALDI-ToF MS signal of the **Cu-L1$_2$** adducts region. The solid black line is the recorded MALDI-ToF MS signal and the blue lines is the expected isotope pattern for a 4/5 mixture of the doubly closed macrocyclic copper adduct (sketched in red) and the copper adduct of the one-fold closed dimer of **L1** (displayed in black).

It seems that in spite of the intramolecular nature of the second oxidative acetylene coupling, the macrocyclization is not particularly favored. The working hypothesis is that the copper complex of the one-fold closed dimer does not arrange the remaining terminal acetylenes in a favorable position for the second ring closing reaction.

All together the unsuccessful template-free macrocyclization attempts contrast the high yields in the macrocyclization reactions of the M(II) complexes and thus underscore the importance of the template approach.
